# Diversity and taxonomy of endophytic *Chaetomiaceae* (*Sordariales*) associated with roots of wild *Orchidaceae* in Brazil

**DOI:** 10.3897/imafungus.17.192047

**Published:** 2026-09-18

**Authors:** Danilo Oliveira Ramos, Pedro Thiago Santos Nogueira, Thiago Oliveira Condé, Jaciara dos Santos Santana, Olinto Liparini Pereira

**Affiliations:** 1 Departamento de Fitopatologia, Universidade Federal de Viçosa, 36570-900, Viçosa, Minas Gerais, Brazil Departamento de Fitopatologia, Universidade Federal de Viçosa Viçosa Brazil; 2 Departamento de Microbiologia, Universidade Federal de Viçosa, 36570-900, Viçosa, Minas Gerais, Brazil Departamento de Microbiologia, Universidade Federal de Viçosa Viçosa Brazil

**Keywords:** *
Arcopilus
*, *
Chaetomium
*, endophytes, new taxa, phylogeny, taxonomy

## Abstract

Fungi in the family *Chaetomiaceae* are ubiquitous, colonizing diverse substrates and frequently occurring as endophytes in various plant species. Despite their prevalence, knowledge of their association with the *Orchidaceae* remains limited. In this study, we investigated the diversity of endophytic fungi associated with the roots of wild orchids. Based on comprehensive morphological and multigene phylogenetic analysis (ITS, LSU, *tub2*, and *rpb2*), 51 isolates were identified as belonging to six *Chaetomiaceae* genera: *Arcopilus*, *Chaetomium*, *Collariella*, *Dichotomopilus*, *Humicola*, and *Pseudohumicola*. Among these, 19 isolates represented ten new species, and 32 isolates were identified as belonging to 12 described species: *Arcopilus amazonicus*, *A. aureus*, and *A. cupreus* were recorded, and four new *Arcopilus* species were described: *A. abbreviatus***sp. nov**., *A. endophyticus***sp. nov**., *A. kasuyae***sp. nov**., and *A. singularis***sp. nov**., for which a description of the asexual morph is provided, offering new insights into the morphological diversity of the genus. Five *Chaetomium* species were identified: *C. coarctatum*, *C. cochliodes*, *C. globosum*, *C. pseudocochliodes*, and *C. tenue*. Furthermore, *Collariella fluminensis***sp. nov**. and *Dichotomopilus brasiliensis***sp. nov**. were proposed, while *D. variostiolatus* was reported for the first time as an orchid root endophyte. Three new species of *Humicola* were discovered: *H. capillata***sp. nov**., *H. paraisoensis***sp. nov**., and *H. purpurea***sp. nov**. Furthermore, *Pseudohumicola crispata***sp. nov**. is described, alongside descriptions of the sexual morph of *Ps. pulvericola* and the acremonium-like synanamorph of *Ps. cinnamomeobrunnea*. Based on morphological analyses and the genealogical concordance phylogenetic species recognition (GCPSR) we propose the synonymization of *A. albae* under *A. cupreus*, and *C. albiziae* under *C. cucumericola*. These findings significantly expand the known diversity of *Chaetomiaceae* and highlight the complex fungal communities associated with wild orchids.

## Introduction

The *Orchidaceae* family is considered one of the most species-rich groups of flowering plants, with 29,524 species distributed across all continents except Antarctica (Chase et al. 2015; Christenhusz and Byng 2016; Govaerts et al. 2021). Among the flowering plants in Brazil, the family *Orchidaceae* is the second most diverse group, behind *Fabaceae*, with terrestrial, lithophytic, and epiphytic habits. It is estimated that around 2,692 native species occur throughout the Brazilian biomes, with approximately 1,490 endemic species that remain poorly studied by science (The Brazil Flora Group et al. 2021). Nevertheless, habitat reduction and the collection of orchids for trade have been the main factors associated with species loss in nature (Mittermeier et al. 2004; Stehmann 2009; Martinelli and Moraes 2013; Fay 2018).

Owing to insufficient nutrients for complete seed germination, symbiotic associations between orchids and fungi are mandatory during their life cycle in the wild (Selosse and Martos 2014). Non-mycorrhizal fungi, mainly *Ascomycota*, often colonize the root tissues of orchids asymptomatically (Ma et al. 2015; Condé et al. 2025; Silva et al. 2026). Although their taxonomic diversity remains poorly studied, this ecological relationship can provide physiological benefits to the host plant, such as plant growth promotion and resistance to pathogens and abiotic stresses (Rodriguez et al. 2009; Ma et al. 2015; Pietro-Souza et al. 2019). In this interaction, the fungus benefits from the plant by obtaining nutrients for its survival and shelter within the tissues of the host.

Orchid roots harbor a highly diverse community of endophytic fungi. However, studies focused on identifying these fungi have mainly relied on the ITS (internal transcribed spacer) region of the rDNA, which is insufficient for accurate species identification (Miranda et al. 2021; Pereira et al. 2024). Several economically important genera have been reported as endophytes in orchids, such as *Chaetomium*, *Penicillium*, *Talaromyces*, and *Trichoderma* (Ma et al. 2015; Condé et al. 2025). Although the genus *Chaetomium* can be found as an endophyte in orchids, most studies have not clarified which specific taxa are involved.

The taxonomy of the *Chaetomiaceae* family has undergone significant changes in recent years with the use of molecular tools. The application of multi-gene molecular phylogeny enabled the correct delimitation of genera within the family that were previously assigned to *Chaetomium*. Furthermore, new genera and species have been described in indoor environments, soil, plant remains, and endophytic associations with different plant families (Wang et al. 2016a, 2019a, 2019b, 2022a). The morphology of sexual and asexual morphs is still an important tool in the taxonomy of chaetomium-like fungi; however, phylogenetic analyses using DNA sequences of the RNA polymerase II second-largest subunit (*rpb2*), β-tubulin (*tub2*), ITS, and 28S large subunit (LSU) regions are paramount for correctly delimiting genera and species in *Chaetomiaceae* (Wang et al. 2016a, 2019b, 2022b).

Although scarce in Brazil, studies involving the taxonomy and description of new *Chaetomiaceae* species have recently been conducted. New species in the genera *Arcopilus* and *Achaetomium* have been described from native Brazilian plants, such as *A. eremanthi*, an endophyte of *Eremanthus erythropappus* (Tavares et al. 2022), *A. amazonicus* associated with *Paullinia cupana* (Sousa et al. 2020), and *Ach. lippiae*, as an endophyte of *Lippia gracilis* (Crous et al. 2017). Other reports on *Chaetomiaceae* in Brazil include studies on coprophilous and saprobic fungi, such as *Chaetomium* species, which are most frequently found in animal dung, whereas *Dichotomopilus*, *Humicola*, and *Staphylotrichum* species can be found in plant debris (Barbosa et al. 2012; Melo et al. 2012, 2019). In addition, Brazilian caves have been shown to be a source of taxonomic discoveries within *Chaetomiaceae*, including new species in the genera *Chaetomium* (*C. meridionalense*), *Humicola* (*H. cerradensis*), and *Pseudohumicola* (*Ps. alba*, *Ps. cecavii*, and *Ps. lutea*), as well as the new genus *Parahumicola* (*Pa. guana* and *Pa. amazonica*) associated with bat dung (Alves et al. 2022; Condé et al. 2023; Nogueira et al. 2025).

As the taxonomic diversity of endophytic *Chaetomiaceae* is still poorly explored, only a few species in the genera *Arcopilus*, *Dichotomopilus*, *Chaetomium*, and *Humicola* have been reported to be endophytic in orchid roots (Currah et al. 1997; Ma et al. 2015). However, there is potential for the discovery of taxonomic and biotechnological novelties among endophytic fungi associated with *Orchidaceae*, as these plants harbor a diversity of fungi associated with their root tissues (Bhatti and Thakur 2022). Therefore, this study aimed to investigate *Chaetomiaceae* isolates obtained from healthy roots of wild orchids exhibiting epiphytic, lithophytic, and terrestrial habits. Using a polyphasic approach combining morphological data and multi-gene phylogenetic analyses of ITS, LSU, *rpb2*, and *tub2* markers, 19 root endophytic isolates were identified as ten new taxa, and new reports of endophytic *Chaetomiaceae* associated with wild Brazilian orchids were provided.

## Material and methods

This study was conducted as part of the PROTAX research project (CNPq-PROTAX 441384/2020-0), which aimed to explore the taxonomic diversity of root endophytic fungi in wild Brazilian orchids and describe new taxa associated with these plants. During surveys across diverse habitats, fungal isolates exhibiting morphological characteristics typical of the *Chaetomiaceae* family were frequently encountered.

### Sample collection and fungal isolation

**Figure 1.**
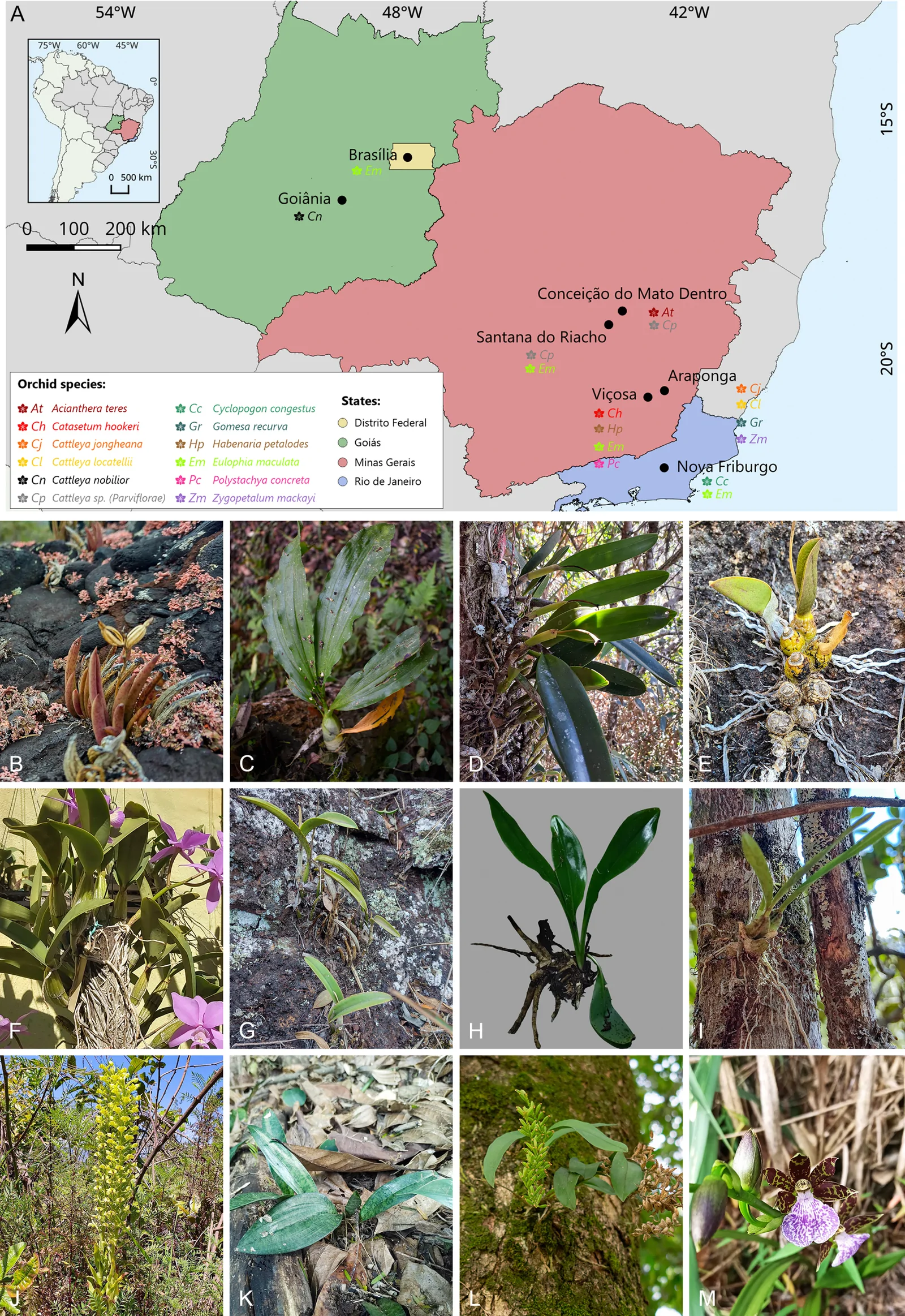
Sampling sites and orchids sampled. **A**. Sampling sites where orchids were collected; **B**. *Acianthera teres*; **C**. *Catasetum hookeri*; **D**. *Cattleya jongheana*; **E**. *Cattleya locatellii*; **F**. *Cattleya nobilior*; **G**. *Cattleya* sp. (*Parviflorae*) **H**. *Cyclopogon congestus*; **I**. *Gomesa recurva*; **J**. *Habenaria petalodes*; **K**. *Eulophia maculata*; **L**. *Polystachya concreta*; **M**. *Zygopetalum mackayi*.

Sample collections of wild orchid roots were performed in two Brazilian biomes, Atlantic Forest and Cerrado (Brazilian savanna), in four states: Distrito Federal, Goiás, Minas Gerais, and Rio de Janeiro (Fig. 1A). Endophytic *Chaetomiaceae* isolates were obtained from healthy wild orchid roots of *Acianthera teres* (Lindl.) Borba, *Catasetum hookeri* Lindl., *Cattleya jongheana* (Rchb.f.) Van den Berg, *Cattleya locatellii* (F.E.L.Miranda) Van den Berg, *Cattleya nobilior* Rchb.f., *Cattleya* sp. (*Parviflorae*) Lindl., *Cyclopogon congestus* (Vell.) Hoehne, *Eulophia maculata* (Lindl.) Rchb.f., *Habenaria petalodes* Lindl., *Gomesa recurva* R.Br., *Polystachya concreta* (Jacq.) Garay & Sweet, and *Zygopetalum mackayi* Hook. (Fig. 1B–M). Orchid species were identified by a specialist in this taxonomic group. Two *Cattleya* species could not be identified at the species level, but they were identified as belonging to the series *Parviflorae* based on morphological analysis of the plant vegetative parts (van Den Berg 2014).

Root endophytic fungi were isolated using the indirect isolation method described by Pereira and Zambolim (2012) and the dilution-to-extinction cultivation method described by Condé et al. (2025) and Oliveira et al. (2024). Additionally, direct isolation of *Chaetomiaceae* structures was performed for saprophytic isolates that were growing on the coconut shell substrate used for the cultivation of *Cattleya aclandiae* that exhibited fungal structures similar to those of members of the *Chaetomiaceae* family. Endophytic fungi with morphological characteristics of *Chaetomiaceae* were subcultured on Oatmeal Agar (OA), and monosporic cultures were obtained. Forty-four isolates were obtained as root endophytes of orchids, and three saprophytic isolates were obtained from the coconut shell substrate used for orchid cultivation. Although these isolates were not endophytic, they were included due to their proximity to orchid roots and their morphological similarity to *Chaetomiaceae*.

In addition, four other isolates were included in this study: two endophytic isolates previously identified as *Chaetomium* sp. from the roots of an unidentified *Cattleya* species collected in 2012 and deposited in the working fungal collection of the Laboratório de Micologia e Etiologia de Doenças Fúngicas at the Universidade Federal de Viçosa (UFV), and two root endophytic isolates obtained from *Musa* sp. and *Theobroma cacao* were included in the analysis because of their high similarity with the orchid endophytic isolates. In total, 51 isolates were analyzed in this study. Representative isolates and ex-type cultures obtained in this study were deposited in the Coleção Octávio Almeida Drummond (COAD), and dried cultures representing the holotypes were deposited in Herbarium VIC, both hosted at the UFV.

### DNA extraction, PCR amplification, and sequencing

Genomic DNA was extracted from 4–7-day-old fungal cultures grown on OA, covered with a sterile cellophane membrane, at 25 °C in the dark, using the Wizard Genomic DNA Purification Kit (Promega) protocol, with modifications according to Pinho et al. (2013). PCR amplifications of the protein-coding genes β-tubulin (*tub2*) and RNA polymerase II (*rpb2*) were performed using the primers T1 (O’Donnell and Cigelnik 1997) and Bt2b (Glass and Donaldson 1995) or Tub4RD (Groenewald et al. 2013) for *tub2*, and fRPB2-5F2 (Sung et al. 2007) and fRPB2-7cR (Liu et al. 1999) for *rpb2*. Initially, the protein-coding genes *tub2* and *rpb2* were amplified for all isolates, as they provide sufficient resolution to identify species in *Chaetomiaceae* (Wang et al. 2016b). Because the internal transcribed spacers (ITS) and 28S large subunit (LSU) regions have limited species-level resolution in the family, they were amplified using the primers ITS5 and LR6 (Vilgalys and Hester 1990; White et al. 1990) only for isolates representing potential novel species. This strategy allowed us to evaluate genealogical concordance across all four loci for candidate new taxa without unnecessary sequencing of known species. The PCR conditions were set as described by Condé et al. (2023). PCR products were visualized on a 0.8% (w/v) agarose gel stained with GelRed™ (Biotium) to verify purity and fragment size. PCR products were purified and sequenced by Macrogen Inc. (South Korea).

### Phylogenetic analyses

The nucleotide sequences obtained were visualized and edited in FinchTV v.1.4.0 (Geospiza Inc.). DNA sequences were compared with those available in the GenBank database (www.ncbi.nlm.nih.gov/Genbank) using megaBLAST. Subsequently, sequence datasets of related genera were constructed using sequences of ex-type and representative isolates retrieved from GenBank (Suppl. material 1). First, individual datasets of *tub2* and *rpb2* were constructed, after which ITS and LSU were added for the new species. Datasets were concatenated using Sequence Matrix 1.8 (Vaidya et al. 2011). Individual and concatenated datasets were aligned using MAFFT v.7 (Katoh and Standley 2013), and manual adjustments were made in MEGA v.7 (Kumar et al. 2016). Bayesian Inference (BI) and maximum likelihood (ML) were performed for phylogenetic analyses. BI analyses were carried out using MrBayes v.3.2.7 (Ronquist et al. 2012) within the CIPRES Science Gateway Portal (Miller et al. 2010). The best-fit nucleotide substitution model was selected using jModelTest v. 2.1.10 (Darriba et al. 2012) based on the Akaike Information Criterion corrected (AICc). Four MCMC chains were conducted simultaneously, starting from random trees and running for up to 10,000,000 generations. Trees were sampled every 1,000 generations, resulting in 10,000 trees. The first 2,500 trees were discarded from the analyses. Posterior probability (pp) values (Rannala and Yang 1996) were determined from the consensus tree using the remaining 7,500 trees. Maximum Likelihood (ML) analyses were performed using IQ-TREE v. 2.2.0 (Minh et al. 2020) with 10,000 ultrafast bootstrap (UFB) replicates (Hoang et al. 2018). Best-fit nucleotide substitution models for each region were determined using ModelFinder (Kalyaanamoorthy et al. 2017); these selected models for both BI and ML analyses are listed in Table 2. The BI phylogenetic trees were chosen for final presentation, with support values (bs ≥ 80% and pp ≥ 0.8) mapped onto the nodes. Trees were visualized in FigTree v. 1.4.3 (Rambaut 2018) and further refined in Adobe Illustrator. All alignments and resulting phylogenetic trees were deposited in Figshare (doi: 10.6084/m9.figshare.31680244).

**Table 1.** *Chaetomiaceae* cultures, GenBank accession numbers, orchid species and collection details obtained in this study.

**Species**	**Culture accession**	**Orchid species/Substrate**	**Orchid habit**	**Location**	**Genbank accession number**			
					**ITS**	**LSU**	***tub2***	***rpb2***
***Arcopilus abbreviatus***	**COAD 3727^T^**	***Habenaria petalodes***	**Terrestrial**	**Viçosa-MG**	**PX712034**	**PX712048**	**PX699208**	**PX705484**
*Arcopilus amazonicus*	COAD 3710	*Cattleya* sp. (*Parviflorae*)	Lithophytic	Conceição do Mato Dentro-MG	–	–	PX699190	PX705486
*Arcopilus amazonicus*	COAD 3711	*Cattleya* sp. (*Parviflorae*)	Lithophytic	Conceição do Mato Dentro-MG	–	–	PX699191	PX705487
*Arcopilus amazonicus*	COAD 3712	*Cattleya* sp. (*Parviflorae*)	Lithophytic	Conceição do Mato Dentro-MG	–	–	PX699192	PX705488
*Arcopilus amazonicus*	COAD 3713	*Cattleya* sp. (*Parviflorae*)	Lithophytic	Conceição do Mato Dentro-MG	–	–	PX699193	PX705489
*Arcopilus amazonicus*	COAD 3714	*Cattleya* sp. (*Parviflorae*)	Lithophytic	Conceição do Mato Dentro-MG	–	–	PX699194	PX705490
*Arcopilus amazonicus*	COAD 3715	*Cattleya* sp. (*Parviflorae*)	Lithophytic	Conceição do Mato Dentro-MG	–	–	PX699195	PX705479
*Arcopilus amazonicus*	COAD 3716	*Cattleya* sp. (*Parviflorae*)	Lithophytic	Conceição do Mato Dentro-MG	–	–	PX699196	PX705491
*Arcopilus amazonicus*	COAD 3717	*Cattleya jongheana*	Epiphytic	Araponga-MG	–	–	PX699197	PX705492
*Arcopilus aureus*	COAD 3718	*Cattleya* sp. (*Parviflorae*)	Lithophytic	Conceição do Mato Dentro-MG	–	–	–	PX705493
*Arcopilus aureus*	COAD 3719	*Cattleya* sp. (*Parviflorae*)	Lithophytic	Conceição do Mato Dentro-MG	–	–	PX699198	PX705494
*Arcopilus aureus*	COAD 3720	*Habenaria petalodes*	Terrestrial	Viçosa-MG	–	–	PX699199	PX705480
*Arcopilus aureus*	COAD 3721	*Habenaria petalodes*	Terrestrial	Viçosa-MG	–	–	PX699200	PX705481
*Arcopilus cupreus*	COAD 3722	*Cattleya* sp.	Unknown	Viçosa-MG	–	–	PX699201	PX705482
***Arcopilus endophyticus***	**COAD 3728^T^**	***Habenaria petalodes***	**Terrestrial**	**Viçosa-MG**	**PX712036**	**PX712049**	**PX699209**	**PX705485**
***Arcopilus kasuyae***	**COAD 3724^T^**	***Acianthera teres***	**Lithophytic**	**Conceição do Mato Dentro-MG**	**PX712037**	**PX712045**	**PX699205**	**PX705497**
***Arcopilus kasuyae***	**COAD 3725**	***Acianthera teres***	**Lithophytic**	**Conceição do Mato Dentro-MG**	**PX712038**	**PX712046**	**PX699206**	**PX705498**
***Arcopilus kasuyae***	**COAD 3726**	***Acianthera teres***	**Lithophytic**	**Conceição do Mato Dentro-MG**	**PX712039**	**PX712047**	**PX699207**	**PX705499**
*Arcopilus macrostiolatus*	COAD 4168	*Eulophia maculata*	Terrestrial	Santana do Riacho-MG	PX712040	–	PX699202	PX705483
*Arcopilus macrostiolatus*	COAD 4169	*Eulophia maculata*	Terrestrial	Santana do Riacho-MG	PX712041	–	PX699203	PX705495
***Arcopilus singularis***	**COAD 3723^T^**	***Cattleya locatellii***	**Lithophytic**	**Araponga-MG**	**PX712035**	**PX712050**	**PX699204**	**PX705496**
*Chaetomium coarctatum*	COAD 3729	*Cyclopogon congestus*	Terrestrial	Nova Friburgo-RJ	–	–	PX692805	PX699189
*Chaetomium cochliodes*	COAD 3730	*Eulophia maculata*	Terrestrial	Nova Friburgo-RJ	–	–	PX692795	PX699182
*Chaetomium cochliodes*	COAD 3731	*Eulophia maculata*	Terrestrial	Nova Friburgo-RJ	–	–	PX692794	PX699183
*Chaetomium cochliodes*	COAD 3760	*Zygopetalum mackayi*	Terrestrial	Araponga-MG	–	–	PX692796	PX699184
*Chaetomium cochliodes*	COAD 3761	*Zygopetalum mackayi*	Terrestrial	Araponga-MG	–	–	PX692797	PX699185
*Chaetomium globosum*	COAD 3732	*Eulophia maculata*	Terrestrial	Viçosa-MG	–	–	PX692802	PX699179
*Chaetomium globosum*	COAD 3759	*Polystachya concreta*	Epiphytic	Viçosa-MG	–	–	PX692804	PX699178
*Chaetomium globosum*	COAD 3762	*Zygopetalum mackayi*	Terrestrial	Araponga-MG	–	–	PX692803	PX699180
*Chaetomium pseudocochliodes*	COAD 3733	*Polystachya concreta*	Epiphytic	Viçosa-MG	–	–	PX692798	PX699186
*Chaetomium pseudocochliodes*	COAD 3734	*Gomesa recurva*	Epiphytic	Araponga-MG	–	–	PX692799	PX699187
*Chaetomium pseudocochliodes*	COAD 3735	*Gomesa recurva*	Epiphytic	Araponga-MG	–	–	PX692800	PX699188
*Chaetomium tenue*	COAD 3736	*Eulophia maculata*	Terrestrial	Viçosa-MG	–	–	PX692801	PX699181
***Collariella fluminensis***	**COAD 3737^T^**	***Cyclopogon congestus***	**Terrestrial**	**Nova Friburgo-RJ**	**PX712032**	**PX712042**	**PX699210**	**PX705500**
***Collariella fluminensis***	**COAD 3738**	***Eulophia maculata***	**Terrestrial**	**Viçosa-MG**	**PX712033**	**PX712043**	**PX699211**	**PX705501**
***Collariella fluminensis***	**COAD 3739**	***Eulophia maculata***	**Terrestrial**	**Viçosa-MG**	**PX712031**	**PX712044**	**PX699212**	**PX705502**
***Dichotomopilus brasiliensis***	**COAD 3741^T^**	***Zygopetalum mackayi***	**Terrestrial**	**Araponga-MG**	**PX710921**	**PX715401**	**PX724932**	**PX724937**
***Dichotomopilus brasiliensis***	**COAD 4294**	***Theobroma cacao***	–	**Vale do Juliana-BA**	–	–	PX724933	PX724938
***Dichotomopilus brasiliensis***	**COAD 4293**	***Musa* sp**.	–	**Itinga do Maranhão-MA**	–	–	PX724934	PX724939
*Dichotomopilus variostiolatus*	COAD 3740	*Cattleya nobilior*	Epiphytic	Goiânia-GO	–	–	PX724935	PX724940
*Dichotomopilus variostiolatus*	COAD 3763	*Cattleya* sp.	Unknown	Viçosa-MG	PX710922	–	PX724936	PX724941
***Humicola capillata***	**COAD 3744^T^**	***Eulophia maculata***	**Terrestrial**	**Brasília-DF**	**PX712013**	**PX712021**	**PX705461**	**PX705473**
***Humicola paraisoensis***	**COAD 3743^T^**	***Catasetum hookeri***	**Terrestrial**	**Viçosa-MG**	**PX712012**	**PX712020**	**PX705460**	**PX705470**
***Humicola paraisoensis***	**COAD 3742**	***Catasetum hookeri***	**Terrestrial**	**Viçosa-MG**	**PX712011**	**PX712019**	**PX705459**	**PX705469**
***Humicola purpurea***	**COAD 3745^T^**	***Eulophia maculata***	**Terrestrial**	**Viçosa-MG**	**PX712014**	**PX712022**	**PX705462**	**PX705471**
***Humicola purpurea***	**COAD 3746**	***Eulophia maculata***	**Terrestrial**	**Viçosa-MG**	**PX712015**	**PX712023**	**PX705463**	**PX705472**
*Pseudohumicola cinnamomeobrunnea*	COAD 3747	Substrate of *Cattleya aclandiae*	–	Viçosa-MG	PX712016	PX712024	PX705466	PX705476
***Pseudohumicola crispata***	**COAD 4166^T^**	***Cattleya* sp**.	**Lithophytic**	**Santana do Riacho-MG**	**PX712018**	**PX712025**	**PX705465**	**PX705478**
***Pseudohumicola crispata***	**COAD 4167**	***Cattleya* sp**.	**Lithophytic**	**Santana do Riacho-MG**	**PX712017**	**PX712026**	**PX705464**	**PX705477**
*Pseudohumicola pulvericola*	COAD 3748	Substrate of *Cattleya aclandiae*	–	Viçosa-MG	–	–	PX705467	PX705475
*Pseudohumicola pulvericola*	COAD 3749	Substrate of *Cattleya aclandiae*	–	Viçosa-MG	–	–	PX705468	PX705474

“^T^” = ex-type strains. New species are indicated in bold.

**Table 2.** Alignment size and optimal nucleotide substitution models selected for Bayesian Inference (BI) and Maximum-Likelihood (ML) analyses.

**Partition**	***Arcopilus + Collariella***			*** Chaetomium ***			*** Dichotomopilus ***			***Humicola + Pseudohumicola***		
	**Length**	**Best model**		**Length**	**Best model**		**Length**	**Best model**		**Length**	**Best model**	
		**BI**	**ML**		**BI**	**ML**		**BI**	**ML**		**BI**	**ML**
ITS	639	GTR+I+G	GTR+F+I+G4	531	K80+I	TNe+G4	577	GTR+G	TIM3e+G4	549	GTR+I+G	TIM2e+I+G4
LSU	564	GTR+I+G	TNe+I+G4	565	HKY+I+G	TNe+I	561	GTR+I	K2P+I	532	GTR+G	TNe+I
*TUB*	822	HKY+I+G	HKY+F+I+G4	735	HKY+I+G	K2P+G4	728	HKY+G	K2P+G4	781	GTR+I+G	HKY+F+I+G4
*RPB2*	852	GTR+I+G	TIM2+F+I+G4	600	GTR+I+G	TIM3+F+I+G4	525	HKY+I+G	TN+F+I+G4	852	GTR+I+G	TIM2+F+I+G4
Concatenated	2877	–	–	2431	–	–	2391	–	–	2714	–	–

### Morphological characterization

The morphological characteristics of the *Chaetomiaceae* isolates were analyzed according to the recommendations of Wang et al. (2022b). Fungal isolates were inoculated at three equidistant points on 90 mm Petri dishes. Colony diameter was measured in Corn Meal Agar (CMA), Malt Extract Agar (MEA), OA and Potato Carrot Agar (PCA) at 25 °C for 7 days in the dark (Crous et al. 2009; Samson et al. 2010). Fungal characters such as the color of mycelium and color reverse, texture of aerial mycelium, presence or absence of ascomata and asexual morphs, and soluble pigment and exudate production were evaluated and described after 3–4 weeks at the same conditions in the culture media mentioned above. Color names were used according to Rayner (1970).

For the description of asexual morphs, the slide culture method (Riddell 1950) and the inclined coverslip method (Kawato and Shinobu 1959; Nugent et al. 2006) were used. Slides were mounted in lactoglycerol or Shear’s solution from structures grown on OA and sometimes PCA. Sexual structures were evaluated after maturation, between 2–4 weeks. Fungal structures were observed with an Olympus BX 53, and images were captured with a Q-color 5 digital camera (Olympus). The morphometry of structures such as ascomata, ascomatal hairs, asci, ascospores, and asexual morphs were obtained using CellSens Dimension 1.9 software. Photographs of the colonies were taken using a Nikon 3400 camera. Close-up photographs of ascomata were taken under a stereomicroscope with a Leica EZ4 camera and a microscope with a Leica DM500 camera using a spotlight. At least 30 measurements were performed for each of the structures mentioned above. Nomenclatural novelties and descriptions were deposited in MycoBank (www.mycobank.org).

### Species delimitation within *Chaetomiaceae*

The new species were delimited using a polyphasic approach that combined multi-locus phylogenetic evidence with detailed macro- and micromorphological characterization. For each putative new taxon, morphological features were compared with those of their closest phylogenetic relatives and descriptions of their respective ex-type strains. Macromorphological data included colony diameter, growth rate, and pigmentation after seven days of incubation on standardized media. Micromorphological analyses focused on the primary diagnostic characters of the *Chaetomiaceae* (Wang et al. 2022b), such as ascomata color; texture of ascomatal wall; morphology and arrangement of terminal and lateral hairs; and the precise shape and size of ascospores and conidia.

Species boundaries were determined following the Genealogical Concordance Phylogenetic Species Recognition (GCPSR) framework (Taylor et al. 2000). For this purpose, both concatenated and individual gene genealogies were evaluated and compared to identify topological congruence and detect potential phylogenetic conflicts. A clade was recognized as a distinct evolutionary lineage if it satisfied at least one of the following criteria: genealogical concordance, evidenced by the consistent recovery of a monophyletic group across multiple independent loci; or genealogical non-contradiction, where a clade is strongly supported in the concatenated analysis and one or more single-locus trees, without being significantly contradicted by any other individual locus. Branch lengths, lineage structure, and statistical support values (Bootstrap and Posterior Probability) were assessed to ensure the robustness of the delimited taxa. This integrative approach enabled the identification of cryptic diversity and ensured that species-level decisions were supported by evolutionary signals from multiple genomic regions.

## Results

### Root endophytic *Chaetomiaceae* isolates and phylogenetic analyses

**Figure 2.**
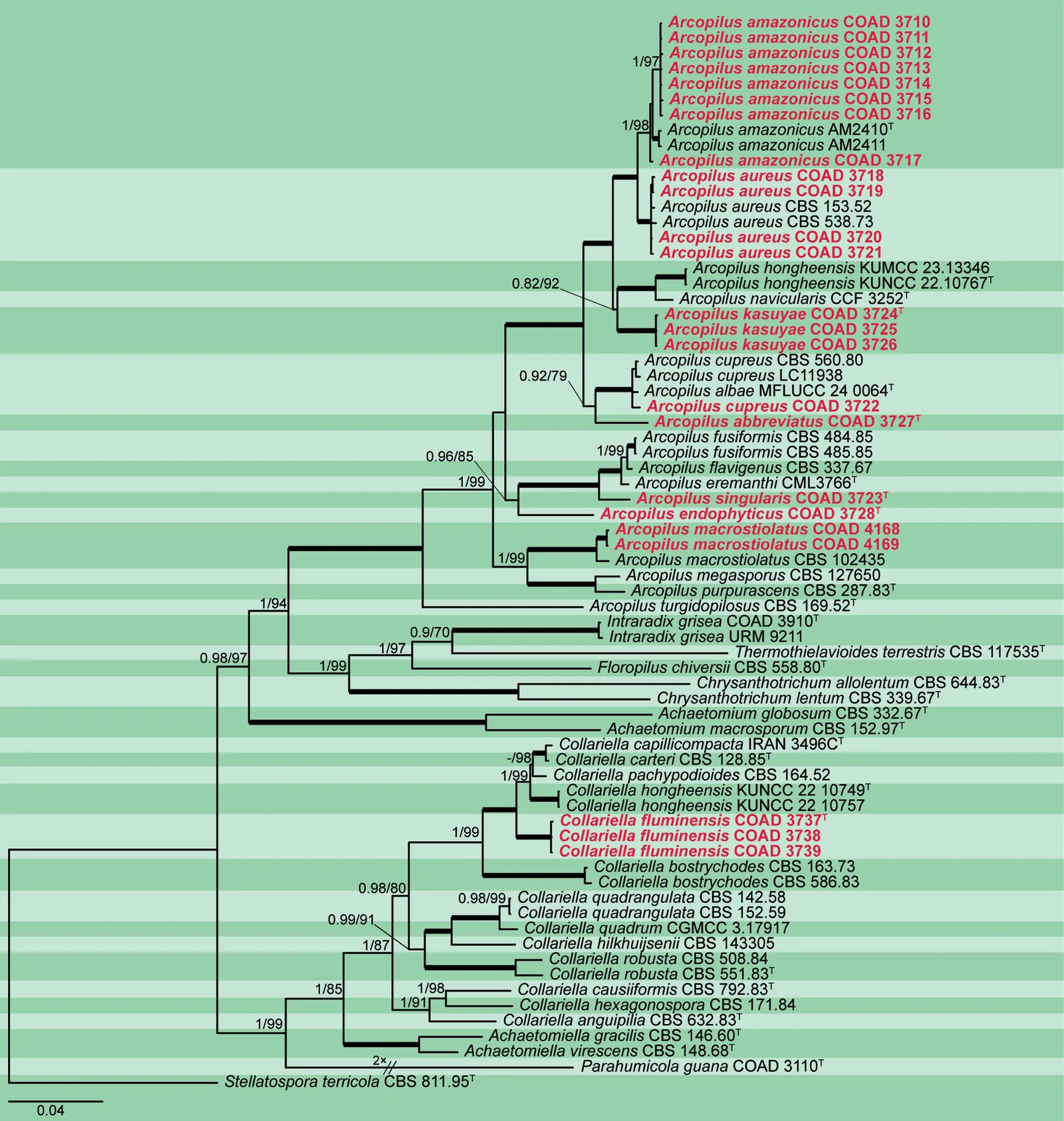
Bayesian phylogenetic tree of *Arcopilus*, *Collariella* and related genera based on a concatenated dataset of ITS, LSU, *rpb2*, and *tub2* sequences. The isolates obtained in this study are shown in bold and red. Ex-type isolates are indicated with” ^T^”. Only values above 80%, for bootstrap (bs), and above 0.80 for posterior probabilities (pp), are shown near nodes. The branches that presented bs = 100% and pp = 1 are thickened. The tree is rooted with *Stellatospora terricola* CBS 811.95.

**Figure 3.**
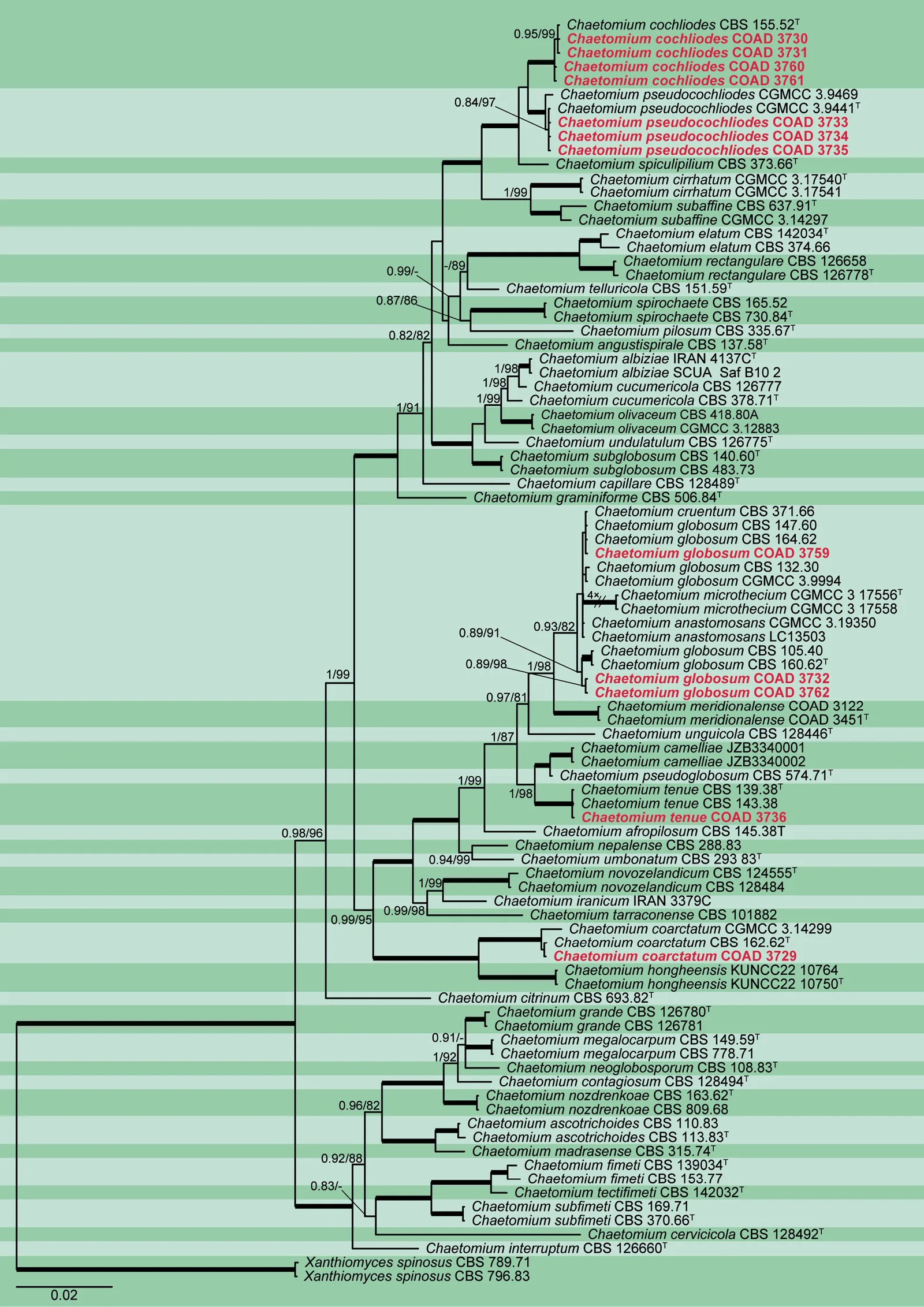
Bayesian phylogenetic tree of *Chaetomium* based on a concatenated dataset of ITS, LSU, *rpb2*, and *tub2* sequences. The isolates obtained in this study are highlighted in bold and red. Ex-type isolates are indicated with” ^T^”. Only values above 80%, for bootstrap (bs), and above 0.80 for posterior probabilities (pp), are shown near the nodes. The branches that presented bs = 100% and pp = 1 are thickened. The tree is rooted with *Xanthiomyces spinosus* CBS 789.71 and CBS 796.83.

**Figure 4.**
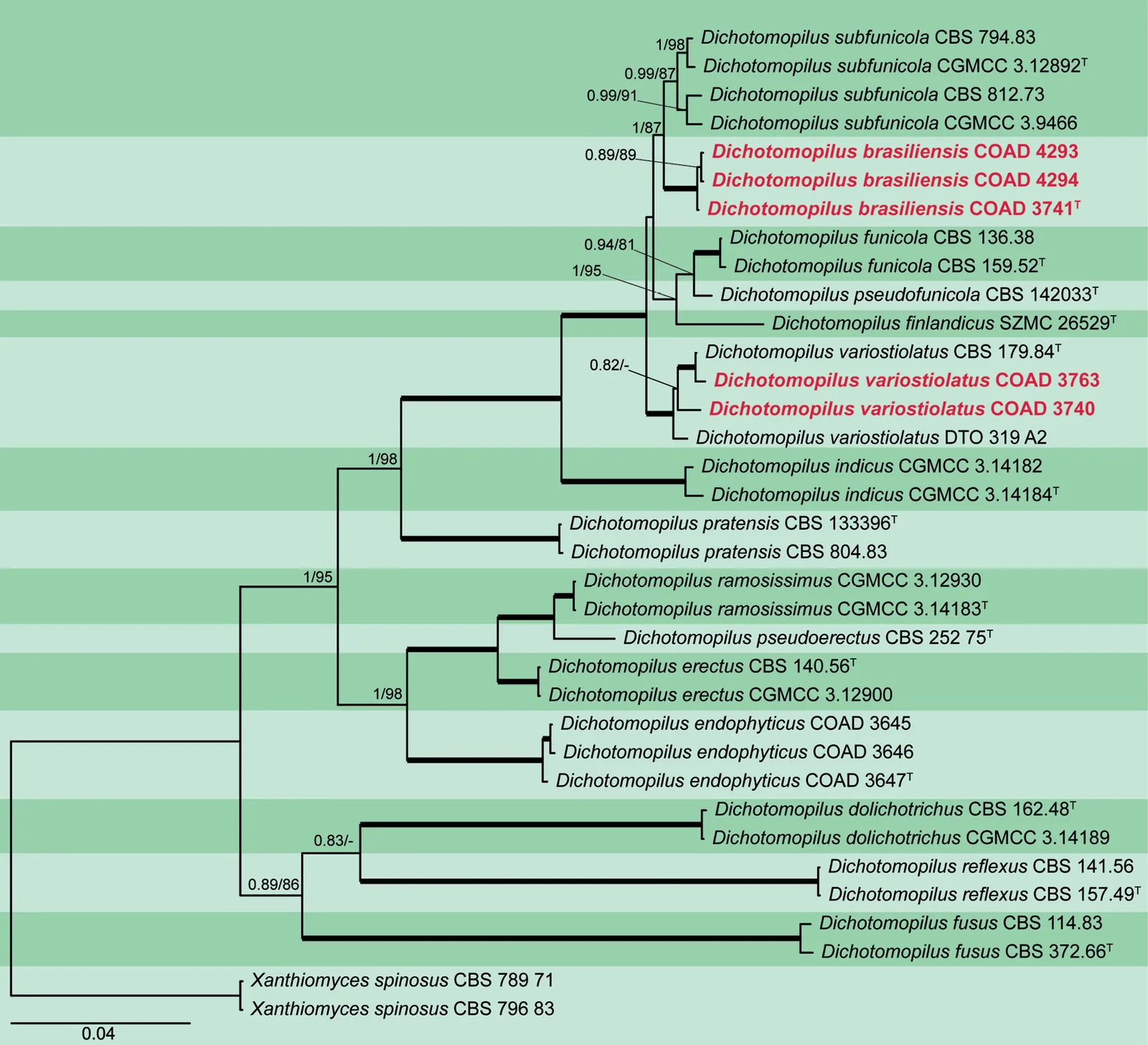
Bayesian phylogenetic tree of *Dichotomopilus* based on a concatenated dataset of ITS, *rpb2*, and *tub2* sequences. The isolates obtained in this study are shown in bold. Ex-type isolates are indicated with “^T^”. Only values above 80%, for bootstrap (bs), and above 0.80 for posterior probabilities (pp), are shown near nodes. The branches that presented bs = 100% and pp = 1 are thickened. The tree is rooted with *Xanthiomyces spinosus* CBS 789.71 and CBS 796.83.

**Figure 5.**
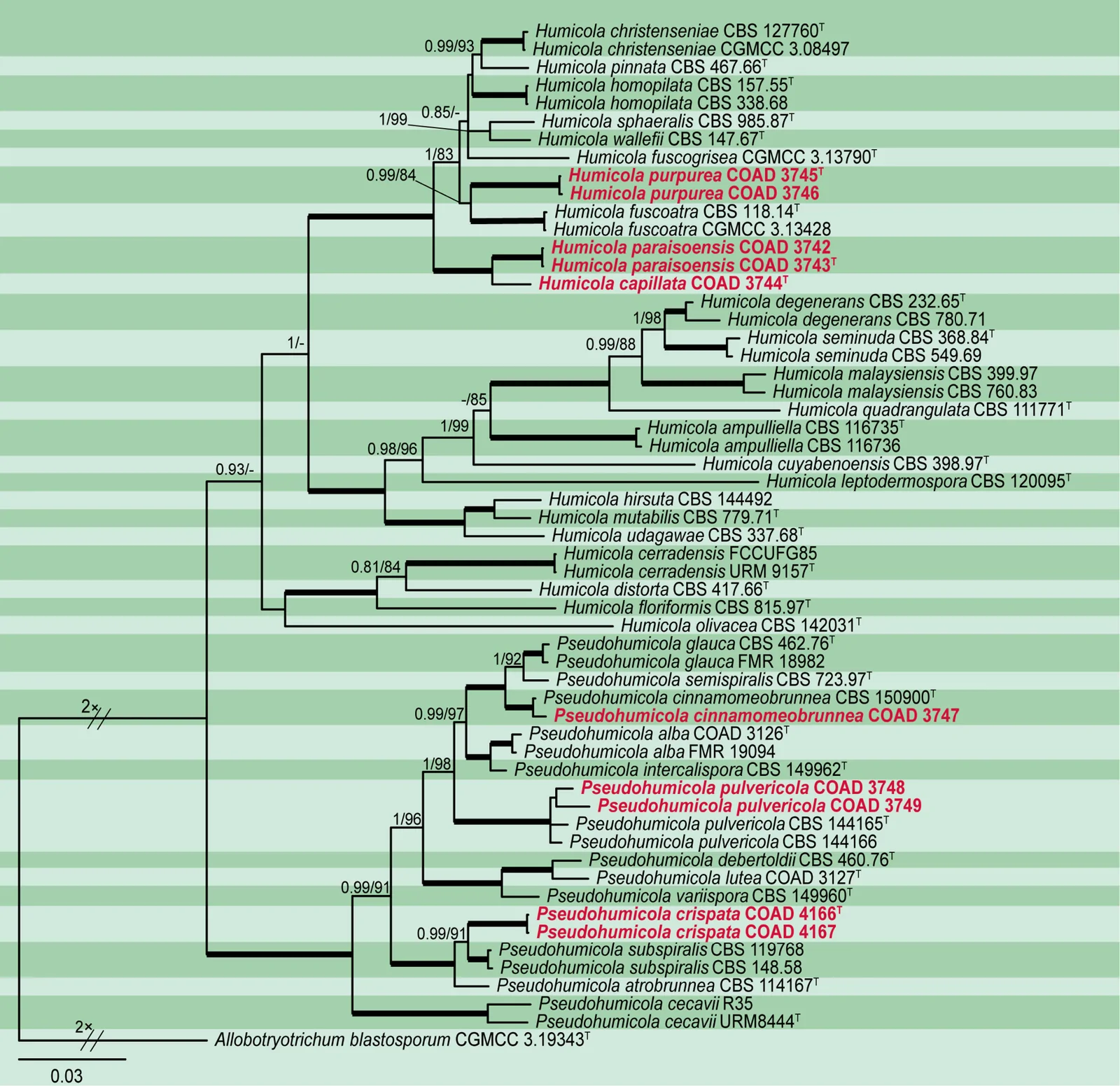
Bayesian phylogenetic tree of *Humicola* and *Pseudohumicola* based on a concatenated dataset of ITS, LSU, *rpb2*, and *tub2* sequences. The isolates obtained in this study are shown in bold. Ex-type isolates are indicated with “^T^”. Only values above 80%, for bootstrap (bs), and above 0.80 for posterior probabilities (pp), are shown near nodes. The branches that presented bs = 100% and pp = 1 are thickened. The tree is rooted with *Allobotryotrichum blastosporum* CGMCC 3.1943.

Forty-four endophytic *Chaetomiaceae* isolates were obtained from the roots of *Orchidaceae* plants, two root endophytic isolates were retrieved from a working fungal collection, and three non-endophytic isolates were obtained from the coconut shell substrate where orchids were grown. In addition, two endophytic isolates obtained from *Musa* sp. and *Theobroma cacao*, which clustered together with the orchid isolates, were added to this study, totaling 51 *Chaetomiaceae* isolates (Table 1). Phylogenetic analyses of *rpb2* and *tub2* sequences revealed that the isolates represented six genera: *Arcopilus*, *Chaetomium*, *Collariella*, *Dichotomopilus*, *Humicola*, and *Pseudohumicola* (Figs 2, 3, 4, 5). However, 19 isolates could not be identified, representing ten new species in the family.

In the genus *Arcopilus*, four previously described species were found as endophytes of orchids: *A. amazonicus* (eight isolates), *A. aureus* (four isolates), *A. cupreus* (one isolate), and *A. macrostiolatus* (two isolates). Six isolates did not cluster with any known species and represented four new species in the genus (Fig. 2). *Arcopilus singularis* sp. nov. was found in *Cattleya locatellii* and clustered in a well-supported lineage close to *A. eremanthi*, *A. flavigenus*, and *A. fusiformis*. *Arcopilus kasuyae* sp. nov. was isolated from *Acianthera teres* and formed a well-supported sister clade to *A. hongheensis* and *A. navicularis*. *Arcopilus abbreviatus* sp. nov. and *A. endophyticus* sp. nov. were isolated from *Habenaria petalodes* root samples. The first clustered in a sister lineage to *A. cupreus*, whereas the latter formed a basal lineage to *A. singularis*.

The phylogeny of *Chaetomium* revealed five species in endophytic associations with orchids (Fig. 3): *C. coarctatum* (one isolate), *C. cochliodes* (three isolates), *C. globosum* (three isolates), *C. pseudocochliodes* (three isolates), and *C. tenue* (one isolate). Additionally, three isolates belonging to the genus *Collariella* represent a novel species, described herein as *Col. fluminensis* sp. nov. This species was isolated from roots of *Cyclopogon congestus* and *Eulophia maculata* and formed a strongly supported clade with *Col. hongheensis*, *Col. capillicompacta*, *Col. carteri*, and *Col. pachypodioides* (Fig. 2). Moreover, five isolates were identified as belonging to *Dichotomopilus*. One isolate retrieved from the working fungal collection, and one obtained from *Cattleya nobilior* were identified as *D. variostiolatus. Dichotomopilus brasiliensis* sp. nov. was isolated from the roots of the orchid *Zygopetalum mackayi* and two other plants (*Musa* sp. and *Theobroma cacao*) and clustered in a well-supported sister lineage to *D. subfunicola* (Fig. 4).

In the phylogenetic tree containing *Humicola* and *Pseudohumicola*, six species were identified, of which four were novel (Fig. 5). *Humicola purpurea* sp. nov. was found in *E. maculata* and clustered in a clade sister to *H. fuscoatra*. *Humicola capillata* sp. nov. was isolated from *Catasetum hookeri* and *H. paraisoensis* sp. nov. from *E. maculata*, and both new species clustered together in a well-supported basal clade. Finally, *Pseudohumicola crispata* sp. nov. was obtained from the roots of *Cattleya* sp., and clustered as a sister species to *Ps. subspiralis* (Fig. 5). Three isolates obtained from the substrate used to cultivate *Cattleya aclandiae* were identified as *Ps. cinnamomeobrunnea* (one isolate) and *Ps. pulvericola* (two isolates).

Phylogenetic analyses of *Chaetomium* revealed two taxonomic inconsistencies. *Chaetomium albiziae* clustered with *C. cucumericola*, and *C. microthecium* was grouped within the *C. globosum* clade (Fig. 3). *Chaetomium albiziae* was originally described as an endophyte of *Albizia lebbeck* (Mehrabi-Koushki and Safi 2023). Pairwise sequence comparisons of ex-type specimens from *C. cucumericola* and *C. albiziae* exhibited a high sequence identity for ITS = 491/492(99%) with one gap, *tub2* = 516/522(99%) without gaps, and *rpb2* = 490/495(99%) without gaps. Furthermore, when we analyzed the two available sequences for each species, there were a negligible number of exclusive nucleotide differences (autapomorphies) across the three loci in our alignment: one transition in the *tub2* alignment (C to T at position 314) and two in *rpb2* (A to G at position 99; C to T at position 231). In all the individual phylogenies, *C. albiziae* was consistently nested within the *C. cucumericola* clade. Furthermore, the presence of sterile isolates and others producing a sexual phase in *Chaetomiaceae* is well documented e.g., *Chaetomium fimeti*, *C. grande*, *C. rectangulare*, *C. subglobosum*, *C. undulatulum* (Wang et al. 2016b), *Parachaetomium hispanicum* (Wang et al. 2022b), and *Pseudohumicola pulvericola* (this study). Therefore, as the original description of *C. cucumericola* (Wang et al. 2016b) was based on a sterile isolate, *C. albiziae* effectively provides a formal description of the sexual morph of this species. Given that *C. albiziae* was described after the most recent revision of accepted *Chaetomiaceae* species (Wang et al. 2022b), and considering the high genetic similarity observed, we propose *C. albiziae* as a heterotypic synonym of *C. cucumericola*.

A similar phylogenetic pattern was observed for *C. microthecium*, which nested within the *C. globosum* species complex in both concatenated and individual ITS and *rpb2* phylogenies (Suppl. material 2: figs S5, S7). However, despite this proximity, a significant number of unique polymorphisms were identified in the *tub2* and LSU alignments, distinguishing *C. microthecium* from the other clade members. According to the Genealogical Concordance Phylogenetic Species Recognition (GCPSR) framework, the lack of congruence across all analyzed loci suggests that these lineages may still be undergoing evolutionary divergence. Furthermore, given that *C. globosum* represents a broad species complex with high intra-clade variability, we consider it premature to propose a synonymization without a comprehensive taxonomic revision of the *C. globosum* species complex. Therefore, we chose to retain *C. microthecium* as a separate species, supported by the distinct molecular signal observed in the *tub2*, which is often more resolute for species delimitation in this genus.

Regarding the genus *Arcopilus*, our multi-locus phylogeny placed *A. albae* in a well-supported clade alongside *A. cupreus* (Fig. 2), with isolate COAD 3722 grouping specifically with the recently described *A. albae* (Xu et al. 2024). We consider that COAD 3722 and the ex-type MFLUCC 24–0064 represent intraspecific variation of *A. cupreus*, given the negligible phylogenetic distance between these lineages. When compared to the reference strain of *A. cupreus* (CBS 560.80), *A. albae* exhibited only two single-nucleotide mutations: a single apomorphy in the *tub2* sequence (G to C at position 101) and one in the ITS region (A to G at position 367), whereas the LSU sequence remained identical. Morphologically, *A. albae* is indistinguishable from *A. cupreus*. Across all individual gene trees, *A. albae* failed to resolve as a distinct lineage from *A. cupreus* (Suppl. material 2: figs S1, S2, S4). Furthermore, the original description of *A. albae* lacked *rpb2* sequence data, a highly informative marker essential for resolving species boundaries within this genus. Consequently, we conclude that *A. albae* was misinterpreted as a distinct taxon, and it is here formally synonymized under *A. cupreus*.

The best nucleotide substitution models for the BI and ML analyses are presented in Table 2.

### Taxonomy

#### Arcopilus

Taxon classificationFungiMonhysteridaChaetomiaceae

X. Wei Wang & Samson, 2016

010E4A01-5A51-53F3-B96D-8672A7386990

##### Type species.

*Arcopilus aureus* (Chivers) X. Wei Wang & Samson, 2016.

#### Arcopilus abbreviatus

Taxon classificationFungiMonhysteridaChaetomiaceae

D.O. Ramos, P.T.S. Nogueira & O.L. Pereira sp. nov.

7EEDC136-1EAD-5643-A308-48B0308BFB11

862440

Fig. 6

##### Etymology.

The epithet refers to the small size of the ascomata produced by this species.

**Figure 6.**
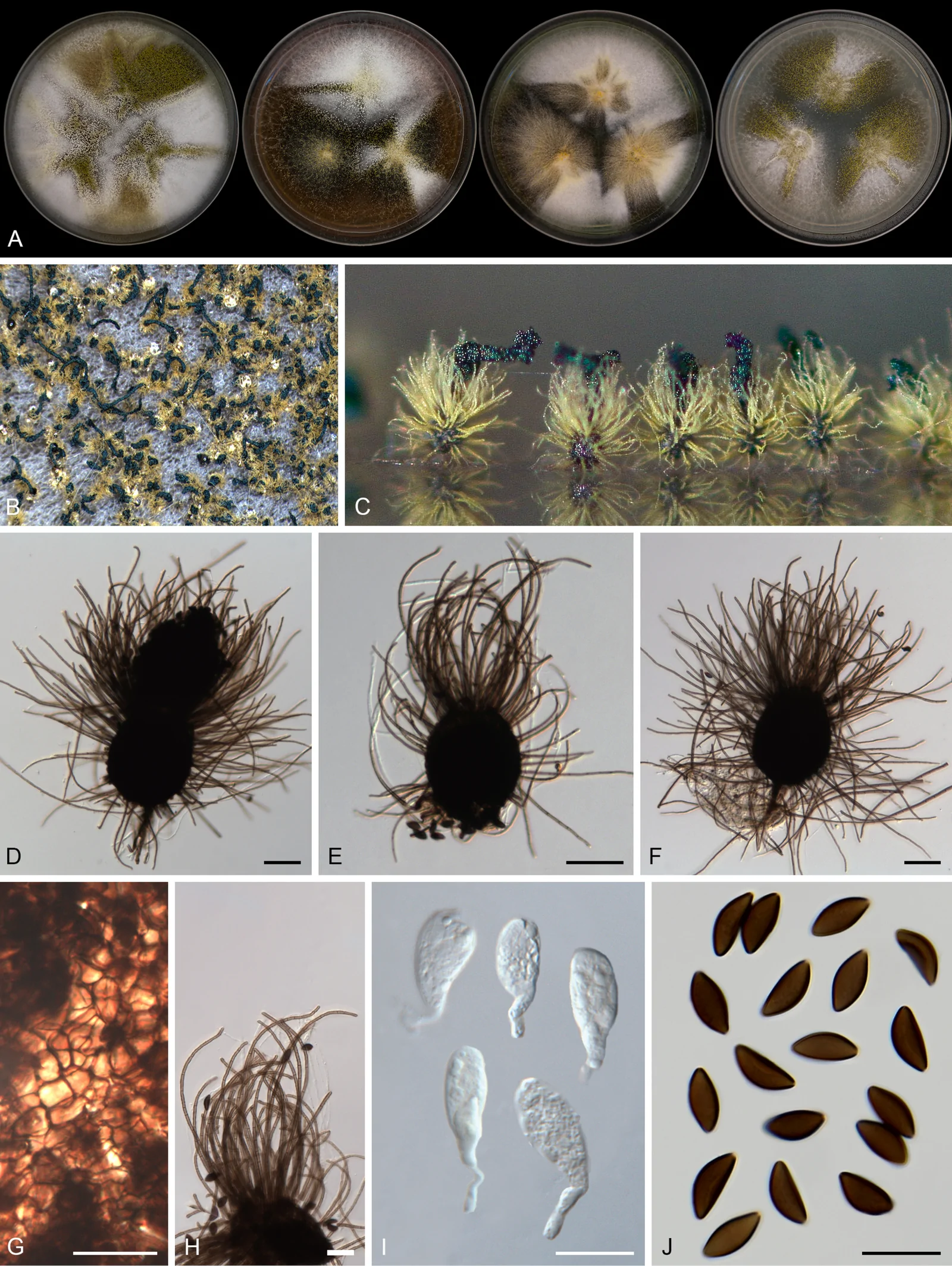
*Arcopilus abbreviatus*COAD 3727, ex-type. **A**. Colonies on OA, CMA, MEA and PCA from left to right after 3 weeks at 25 °C in the dark; **B**. Top view of mature ascomata on OA; **C**. Side view of mature ascomata. **D–F**. Ascomata; **G**. Surface of ascomatal wall; **H**. Terminal ascomatal hairs; **I**. Asci; **J**. Ascospores. Scale bars: 50 μm (**D–F**); 20 μm (**G, H, I**); 10 μm (**J**).

##### Typification.

BRAZIL • Minas Gerais: Viçosa, Zona Rural, from roots of *Habenaria petalodes*, April 2023, O.L. Pereira, D.O. Ramos, and P.T.S. Nogueira (***holotype*** VIC 49498 preserved as dried culture, culture ***ex-type***COAD 3727).

##### Description.

***Ascomata*** superficial, ostiolate, primrose in reflected light owing to ascomatal hairs, globose or subglobose, 108.5–140.5 μm high, 88.5–123 μm diam. ***Ascomatal wall*** brown with ***textura angularis***. ***Terminal hairs*** slightly arcuate sometimes straight, brown, septate. ***Lateral hairs*** recurved, sometimes straight, brown. ***Asci*** fasciculate, clavate, spore-bearing portion 22.5–30 × 10–15.5 μm, stalks 9–14.5 μm long, with 8 biseriate ascospores, evanescent. ***Ascospores*** dark brown, navicular in side view and fusiform in top view, smooth, bilaterally flattened, (12.5–)13.5–14.5(–15.5) × (6–)6.5–7.5(–8) μm in face view, with one apical germ pore. ***Asexual morph*** not observed.

***Culture characteristics***: Colonies on OA attaining 40–43 mm diameter after 7 days at 25 °C, edge entire, white aerial mycelium moderate, flat, circular shape; reverse gray olivaceous at the center, pale luteous at the margins of the colony and with vinaceous soluble pigment. Colonies on CMA attaining 38–40 mm diameter after 7 days at 25 °C, entire margin, white aerial mycelium moderate, raised, circular, reverse olivaceous gray at the center of colonies and vinaceous at the edges owing to the presence of soluble pigment. Colonies on MEA attaining 40–42 mm diameter after 7 days at 25 °C, edge entire, white aerial mycelium moderate to profuse, flat, circular; reverse dark brick at the center and rosy buff at the margins of colonies. Colonies on PCA attaining 43–46 mm diameter after 7 days at 25 °C, edge entire, white aerial mycelium moderate, raised, circular; reverse greenish olivaceous, pale luteous at the margins of the colony and with little production of rosy vinaceous soluble pigment.

##### Distribution.

Brazil (Seasonal Semideciduous Forest within the Atlantic Forest domain in Minas Gerais state).

##### Notes.

Based on phylogenetic analyses of a combined ITS-LSU-*tub2*-*rpb2* alignment, *Arcopilus abbreviatus* clustered in a unique lineage in a clade containing *A. cupreus*. Morphologically, compared to the description of *A. cupreus* by Ames (1949), *A. abbreviatus* has primrose ascomatal hairs and less arcuate terminal ascomatal hairs, which differ from the bright copper-colored ascomata produced by *A. cupreus*. Furthermore, *A. abbreviatus* has larger, navicular to fusiform ascospores, while *A. cupreus* produces smaller, globose to ovoid ascospores ((12.5–)13.5–14.5(–15.5) × (6–)6.5–7.5(–8) μm *vs*. 8.5–11.5 × 5–5.5 μm). Compared to other species in the genus, *A. abbreviatus* produces slightly smaller ascomata and terminal ascomatal hairs less arcuate.

#### Arcopilus endophyticus

Taxon classificationFungiMonhysteridaChaetomiaceae

D.O. Ramos, P.T.S. Nogueira & O.L. Pereira sp. nov.

1518CD4F-85EC-553C-961B-69184249E1A3

862441

Fig. 7

##### Etymology.

Name refers to the endophytic lifestyle of the fungus.

**Figure 7.**
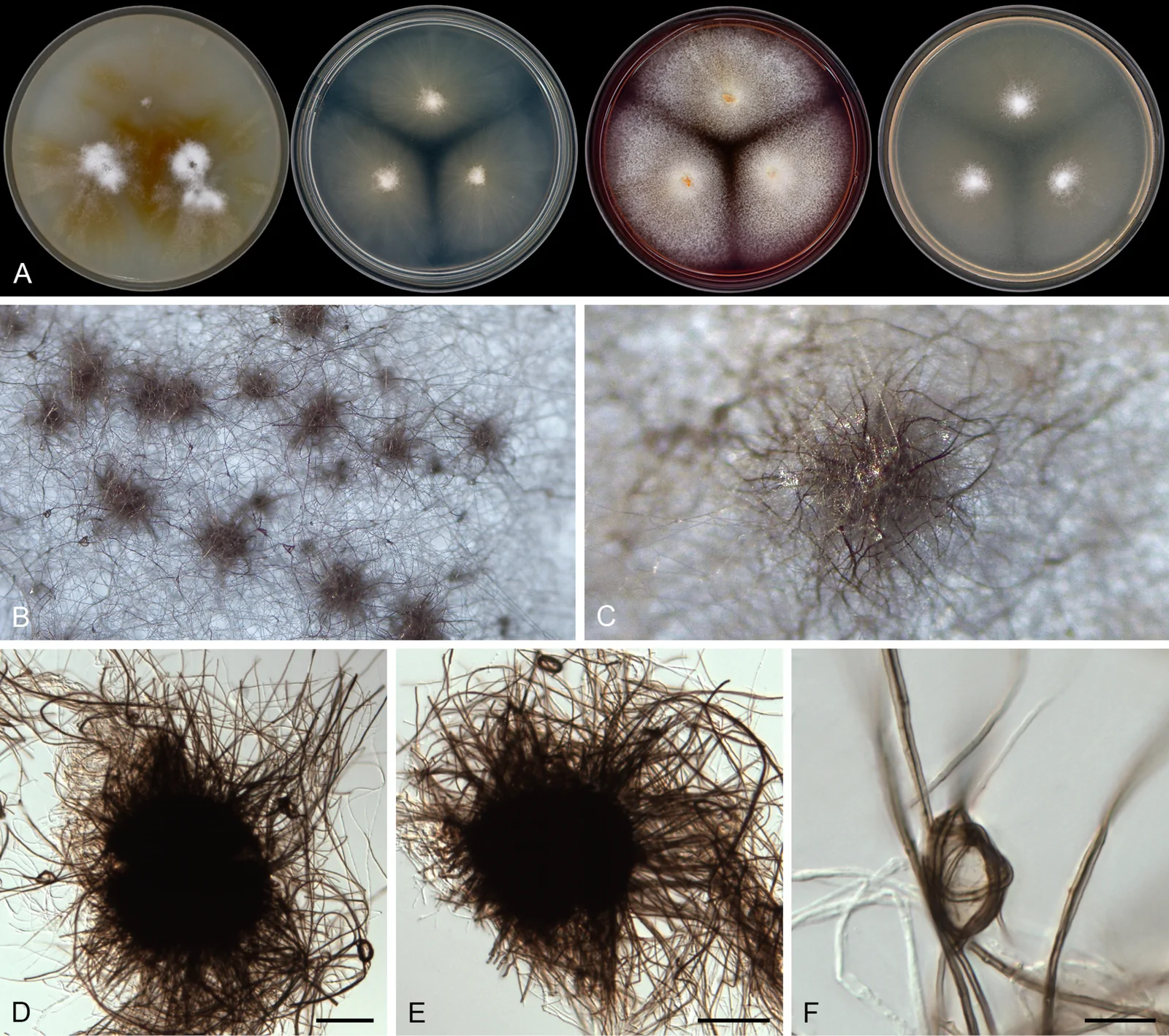
*Arcopilus endophyticus*COAD 3728, ex-type. **A**. Colonies on OA, CMA, MEA and PCA from left to right after 4 weeks at 25 °C in the dark; **B, C**. Top view of ascomata-like structures on OA; **D, E**. Ascomata-like structures; **F**. Coiled hyphae. Scale bars: 100 μm (**D, E**); 20 μm (**F**).

##### Typification.

BRAZIL • Minas Gerais: Viçosa, Zona Rural, from roots of *Habenaria petalodes*, April 2023, O.L. Pereira and D.O. Ramos (***holotype*** VIC 49499 preserved as dried culture, culture ***ex-type***COAD 3728).

##### Description.

*Mycelium sterile*. ***Somatic hyphae*** hyaline, with melanized hyphae forming coiled structures. Structures of tangled, melanized and compact mycelium similar to ascomata initials were observed.

***Culture characteristics***: Colonies on OA attaining 45–50 mm diameter after 7 days at 25 °C, edge entire, aerial mycelium absent, flat, circular, no ascomata production; reverse brick to cinnamon due to the production of soluble pigment. Colonies on CMA attaining 38–44 mm diameter after 7 days at 25 °C, entire margin, white aerial mycelium sparse, flat, circular, no ascomata production; reverse rosy buff; soluble pigments absent. Colonies on MEA attaining 40–47 mm diameter after 7 days at 25 °C, edge entire, white aerial mycelium moderate with exudates above, flat, circular, no ascomata production; reverse scarlet to rust due to the production of soluble pigments. Colonies on PCA attaining 34–42 mm diameter after 7 days at 25 °C, edge entire or slightly undulate, white aerial mycelium moderate at the center of colonies and sparse at the edges, flat, circular, no ascomata production; reverse buff; soluble pigments absent.

##### Distribution.

Brazil (Seasonal Semideciduous Forest within the Atlantic Forest domain in Minas Gerais state).

##### Notes.

In the concatenated phylogeny, *Arcopilus endophyticus* clustered as a basal lineage within a clade containing *A. singularis*, *A. eremanthi*, *A. flavigenus*, and *A. fusiformis* (Fig. 2). Morphologically, this species is distinguished from all other members of the genus by the lack of both asexual and sexual morphs under the tested conditions. Compared to *A. eremanthi*, *A. endophyticus* exhibited significantly higher radial growth on OA (45–50 mm vs 30–31 mm), MEA (40–47 mm vs 41 mm), and PCA (34–42 mm vs 35 mm) (Tavares et al. 2022). Regarding individual gene genealogies, *A. endophyticus* clustered as a sister species to *A. singularis* in the LSU phylogeny (Suppl. material 2: fig. S2). However, in the *tub2* tree, it formed a clade external to *A. macrostiolatus*, *A. megasporus*, and *A. purpurascens* (Suppl. material 2: fig. S4), whereas the *rpb2* phylogeny recovered the same topology observed in the concatenated analysis (Suppl. material 2: fig. S3). Further comparative details are provided in the taxonomic notes for *A. singularis*.

#### Arcopilus kasuyae

Taxon classificationFungiMonhysteridaChaetomiaceae

D.O. Ramos, P.T.S. Nogueira & O.L. Pereira sp. nov.

B3F21DB2-0811-5DA3-A41C-C0B238C9CB64

862442

Fig. 8

##### Etymology.

Named in honor of Maria Catarina Megumi Kasuya, a Brazilian microbiologist who pioneered studies on the interaction between orchids and fungi in Brazil.

**Figure 8.**
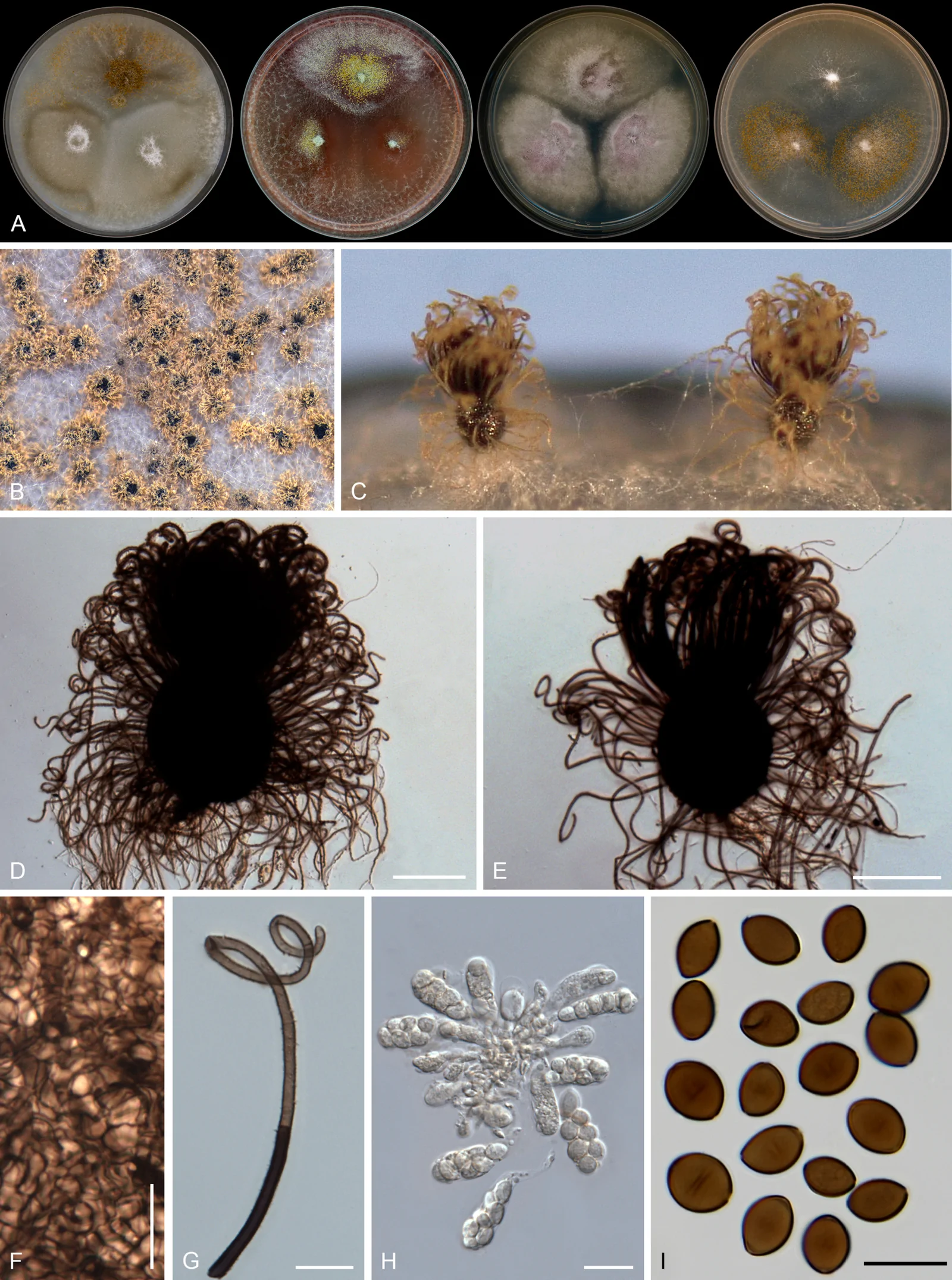
*Arcopilus kasuyae*COAD 3724, ex-type. **A**. Colonies on OA, CMA, MEA and PCA from left to right after 4 weeks at 25 °C in the dark; **B**. Top view of mature ascomata on OA; **C**. Side view of mature ascomata; **D, E**. Ascomata; **F**. Surface of ascomatal wall; **G**. Terminal ascomatal hair; **H**. Asci; **I**. Ascospores. Scale bars: 100 μm (**D, E**); 20 μm (**F, G, I**); 10 μm (**J**).

##### Typification.

BRAZIL • Minas Gerais: Conceição do Mato Dentro, Curral de Pedras, from roots of *Acianthera teres*, 10 April 2022, F.A. Custódio (***holotype*** VIC 49506 preserved as dried culture, culture ex-type COAD 3724).

##### Description.

***Ascomata*** superficial, ostiolate, saffron in reflected light owing to ascomatal hairs, globose to subglobose, 164–251.5 μm high, 128–214 μm diam., homothallic. ***Ascomatal wall surface*** brown with ***textura angularis***. ***Terminal hairs*** arcuate, apically coiled, brown, septate, verrucose, 3–5.5 μm wide near the base. ***Lateral hairs*** arcuate or inequilaterally undulated. ***Asci*** clavate, spore-bearing portion 26.5–40 × 10.5–14.5(–17.5) μm, stalks 13.5–23.5 μm long, with eight biseriate ascospores, evanescent. ***Ascospores*** dark brown, navicular in side view and fusiform in top view, smooth, 11–12 (–13.5) × (7.5–) 7–8 (–9) μm in face view, with two germ pores. ***Asexual morph*** unknown.

***Culture characteristics***: Colonies on OA attaining 33–39 mm diameter after 7 days at 25 °C, edge lobate, white aerial mycelium sparse to absent, flat, circular, no ascomata production; reverse honey to hazel due to the production soluble pigment. Colonies on CMA attaining 33–36 mm diameter after 7 days at 25 °C, entire margin, white aerial mycelium moderate, flat, circular; reverse vinaceous to rust due to the production of soluble pigments. Colonies on MEA attaining 33–35 mm diameter after 7 days at 25 °C, edge entire, white aerial mycelium moderate, with rosy vinaceous exudates above, flat, circular; reverse rosy buff to rosy vinaceous due to production of soluble pigments. Colonies on PCA attaining 38–40 mm diameter after 7 days at 25 °C, edge entire, white aerial mycelium moderate at the center of colonies and sparse at the margins, raised, circular; reverse buff; soluble pigments absent.

##### Additional materials examined.

BRAZIL • Minas Gerais: Conceição do Mato Dentro municipality, Curral de Pedras, from roots of *Acianthera teres*, 10 April 2022, F.A. Custódio (living cultures COAD 3725 and COAD 3726).

##### Distribution.

Brazil (Rupestrian Grasslands within the Atlantic Forest domain in Minas Gerais state).

##### Notes.

Phylogenetically, *Arcopilus kasuyae* is clustered in a unique lineage in a clade related to the species *A. hongheensis* and *A. navicularis* (Fig. 2). A distinctive feature of *A. kasuyae* is the absence of pigmented exudate production in almost all the tested culture media and conditions. Vinaceous soluble pigments were only observed on CMA after 3–4 weeks. The production of exudates, typically yellow, orange, or red, is a cultural characteristic commonly observed in *Arcopilus* species and is present in all related species. Furthermore, compared to the descriptions of *A. hongheensis* by Yang et al. (2023), *A. kasuyae* exhibits larger ascomata (118.5–209.5 × 103–191.5 μm *vs*.100–140 × 80–95 μm), and two apical germ pores compared with one of *A. hongheensis*. The ascomata of *A. kasuyae* are globose or subglobose, whereas *A. hongheensis* produces ellipsoid or subglobose ascomata. Moreover, *A. kasuyae* also possesses larger ascospores when compared to *A. navicularis* (11–12(–13.5) × 7–8(–9) μm *vs*.7.2–8.8 × 4.5–5.5 μm), and saffron ascomatal hairs compared to pale green or greenish olivaceous of *A. navicularis* (Crous et al. 2021).

#### Arcopilus singularis

Taxon classificationFungiMonhysteridaChaetomiaceae

D.O. Ramos, P.T.S. Nogueira & O.L. Pereira sp. nov.

B5E36B92-BDA0-5EA2-95AB-C591965DFB17

862443

Fig. 9

##### Etymology.

Name refers to the unique and characteristic morphology of this species, due to the production of the asexual morph, which is being described for the first time for the genus *Arcopilus*.

**Figure 9.**
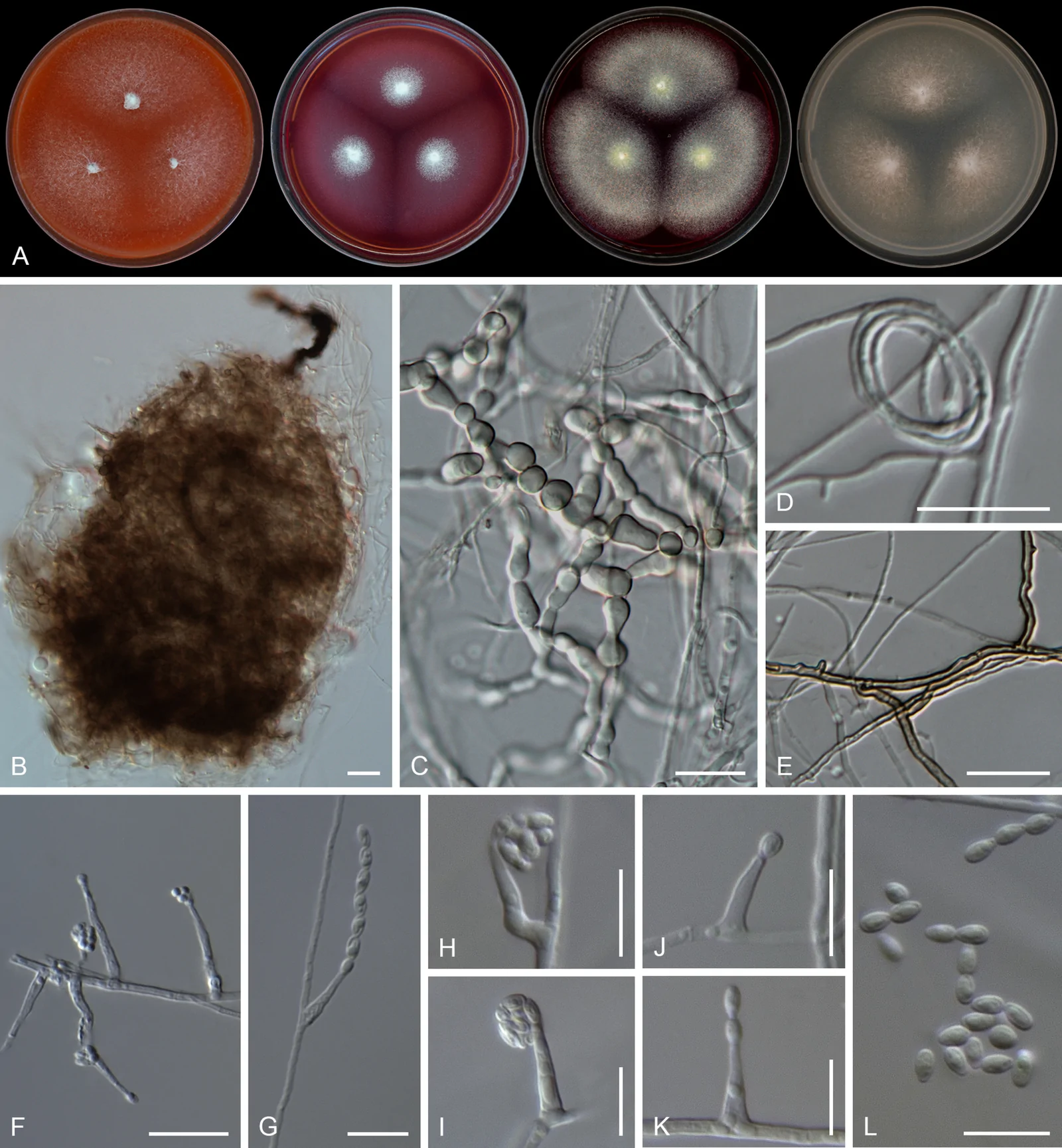
*Arcopilus singularis*COAD 3723, ex-type. **A**. Colonies on OA, CMA, MEA and PCA from left to right after 4 weeks at 25 °C in the dark; **B**. Ascomata initials; **C**. Chlamydospores; **D**. Hyaline coiled hyphae; **E**. Melanized hyphae; **F–K**. Acremonium-like conidiophores and conidia; **L**. Hyaline conidia. Scale bars: 20 μm (**B–E**); 10 μm (**F**); 5 μm (**G–L**).

##### Tyification.

BRAZIL • Minas Gerais: Araponga municipality, Pedra Redonda, from roots of *Cattleya locatellii*, 21 November 2021, O.L. Pereira, D.O. Ramos and P.T.S. Nogueira (***holotype*** VIC 49497 preserved as dried culture, culture ***ex-type***COAD 3723).

##### Description.

***Somatic hyphae*** hyaline, 1–5 μm wide. ***Chlamydospores*** produced intercalary or terminal on hyphae, occasionally solitary or most common moniliform shape or in clustering to form a microsclerotia, globose, subglobose, obovoid, pyriform, occasionally cylindrical or reniform, hyaline to dark brown when mature, smooth, thick-walled when mature, 5.5–15 × 3.5–8.5 μm. ***Acremonium-like conidiophores*** arising laterally from hyphae, reduced a phialidic conidiogenous cells, hyaline, smooth, (4–)7–20.5 × 2–2.5(–3.5) μm. ***Conidia*** borne in basipetal chains or held in false heads, aseptate, clavate, sometimes cylindrical, hyaline, smooth, 2.5–4 × 1.5–2 μm. ***Sexual morph*** unknown.

***Culture characteristics***: Colonies on OA attaining 34–37 mm diameter after 7 days at 25 °C, edge entire, white aerial mycelium sparse, flat, circular, no ascomata production; reverse orange to apricot due to the intense production of soluble pigment. Colonies on CMA attaining 48–50 mm diameter after 7 days at 25 °C, entire margin, white aerial mycelium sparse, flat, circular, no ascomata production; reverse scarlet due to the production of soluble pigment. Colonies on MEA attaining 46–48 mm diameter after 7 days at 25 °C, edge entire, white aerial mycelium moderate at the center of colonies and sparse at the margins, flat, circular, no ascomata production; reverse red to scarlet due to the production of soluble pigments. Colonies on PCA attaining 36–39 mm diameter after 7 days at 25 °C, edge entire, white aerial mycelium moderate at the center of colonies and sparse at the margins, flat, circular, no ascomata production; reverse rosy buff at the center of the colonies and buff at the margins; soluble pigments absent.

##### Distribution.

Brazil (Rupestrian Grasslands within the Atlantic Forest domain in Minas Gerais state).

##### Notes.

Phylogenetic analysis revealed that *Arcopilus singularis* clustered in a well-supported lineage related to *A. endophyticus*, *A. eremanthi*, *A. fusiformis*, and *A. flavigenus*. However, *A. singularis* and *A. endophyticus* are morphologically aberrant compared to the other species, as they are the first in the genus in which the sexual morph has not been observed. Notably, *A. singularis* is, as far as it is known, the first species in the genus to produce an acremonium-like anamorph. The original descriptions of *A. fusiformis* (≡ *Chaetomium fusiforme*) by Chivers (1912) and *A. flavigenus* (≡ *Chaetomium flavigenum*) by van Warmelo (1966) included only the sexual morph and lacked cultural descriptions, which prevents a thorough comparison with *A. singularis* and *A. endophyticus*. Additionaly, *A. singularis* differs from *A. eremanthi* by exhibiting greater growth on culture media, including OA (34–37 *vs*. 30–31 mm), MEA (46–48 *vs*. 41 mm), and PCA (36–39 *vs*. 35 mm) (Tavares et al. 2022). *Arcopilus singularis* differs from *A. endophyticus* by the production of an acremonium-like anamorph, chlamydospores, reduced growth on OA (34–37 *vs*. 45–50 mm) and PCA (36–39 *vs*. 34–42 mm), and greater growth on CMA (48–50 *vs*. 38–44 mm) and MEA (46–48 *vs*. 40–47 mm).

#### Arcopilus amazonicus

Taxon classificationFungiMonhysteridaChaetomiaceae

T.F. Sousa & G.F. Silva, 2020

BC2368AD-8B96-5BD2-9F12-CE4E8D6DA0CF

Fig. 10

##### Typification.

BRAZIL • Amazonas, in the leaves of *Paullinia cupana*, 24 December 2017, T.F. Sousa (***holotype*** INPA2410).

**Figure 10.**
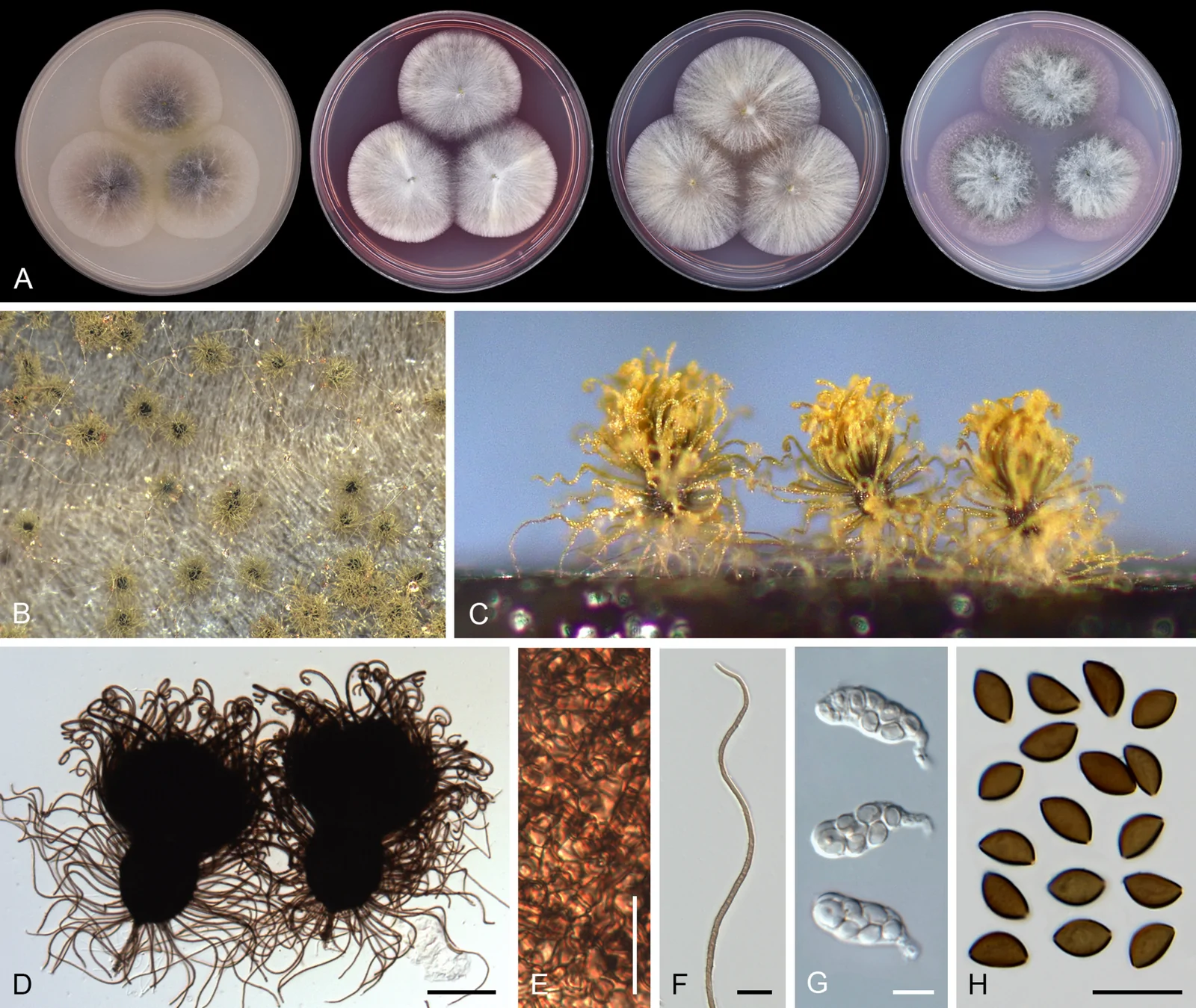
*Arcopilus amazonicus*COAD 3717. **A**. Colonies on OA, CMA, MEA and PCA from left to right after 7 days at 25 °C in the dark; **B**. Top view of ascomata on OA; **C**. Side view of mature ascomata; **D**. Ascomata; **E**. Asci; **F**. Ascospores; **G**. Terminal ascomatal hair; **H**. Surface of ascomatal wall. Scale bars: 100 μm (**D**); 20 μm (**E, F**); 10 μm (**G, H**).

##### Description.

See Sousa et al. (2020).

##### Specimens examined.

BRAZIL • Minas Gerais: Araponga, from roots of *Cattleya jongheana*, Febuary 2018, collected and isolated by E.F.S. Freitas (living culture COAD 3717); Conceição do Mato Dentro municipality, Serra da Ferrugem, isolated from roots of *Cattleya* sp. (*Parviflorae*), 10 April 2022, T.O. Condé and D.O. Ramos (living cultures COAD 3710, COAD 3711, COAD 3712, COAD 3713, COAD 3714, COAD 3715, and COAD 3716).

##### Distribution.

Brazil (Amazon rainforest, Rupestrian Grasslands within the Atlantic Forest domain in Minas Gerais state).

##### Notes.

*Arcopilus amazonicus* was described as an endophyte on the leaves of guarana (*Paullinia cupana*) plants in the Brazilian Amazon region (Sousa et al. 2020). This species is phylogenetically close to *A. aureus* and is distinct only by *rpb2* sequences. The isolate COAD 3717 differs from the ex-type of *A. amazonicus* INPA2410 by colonies on OA, exhibiting an olivaceous black color at the center of colonies and absence of purple exudate diffused in the medium, ascomata subglobose, undulate lateral hairs, and undulate and apically coiled terminal hairs. In this study, *A. amazonicus* was identified in the roots of two orchids from the Atlantic Forest and Cerrado: *Cattleya jongheana* and *Cattleya* sp. (*Parviflorae*). We expanded the known distribution of this species in Brazil, which indicates that *A. amazonicus* may be widely distributed throughout the country. To the best of our knowledge, this is the first report of this species as a root endophyte in plants of the *Orchidaceae* family.

#### Arcopilus aureus

Taxon classificationFungiMonhysteridaChaetomiaceae

(Chivers) X. Wei Wang & Samson, 2016

DEBB1D6A-87DC-510E-BD33-5FBA965230D1

Fig. 11

##### Synonyms.

*Chaetomium aureum* Chivers, Proc. Amer. Acad. Arts 48 (4): 86 (1912).

**Figure 11.**
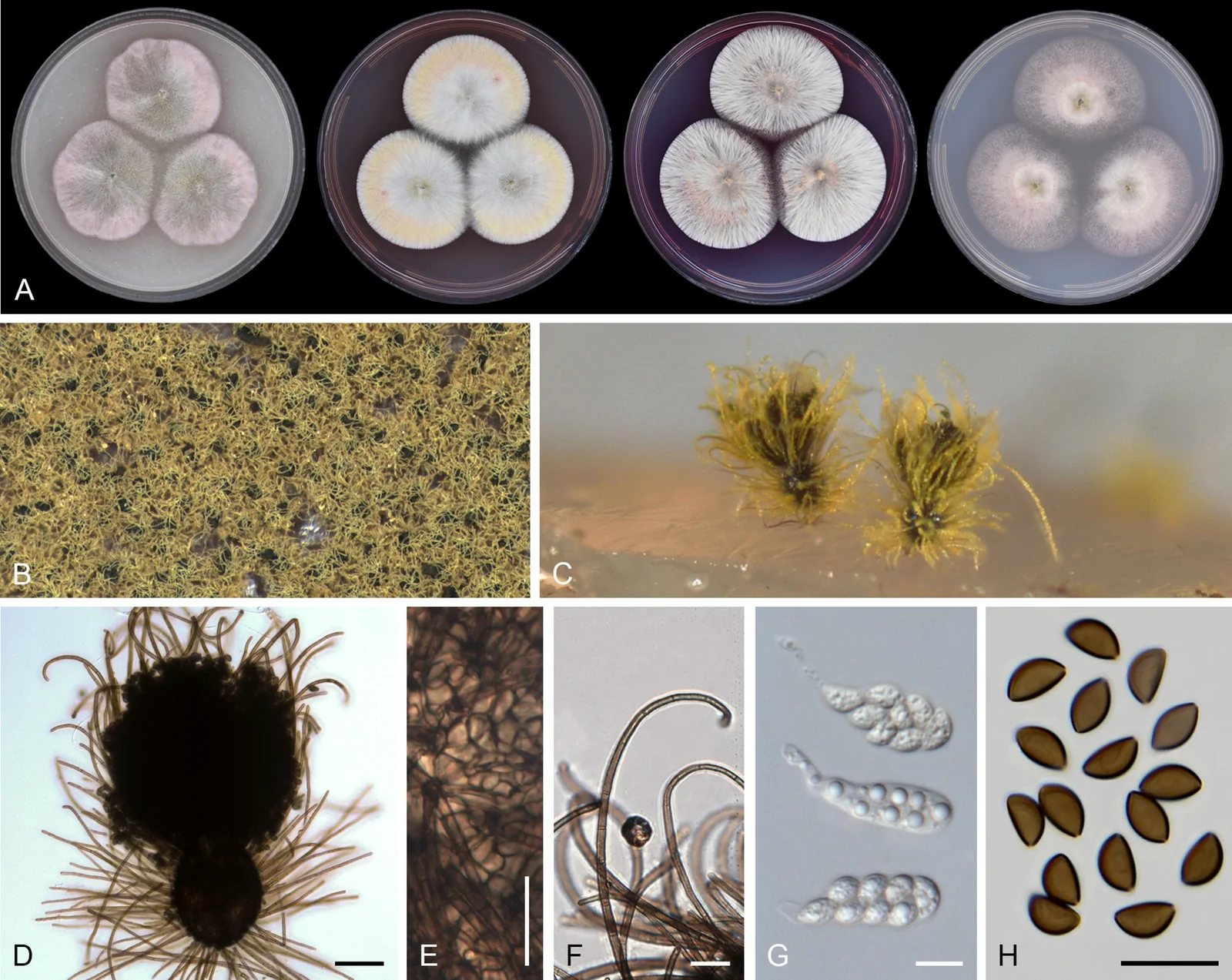
*Arcopilus aureus*COAD 3721. **A**. Colonies on OA, CMA, MEA and PCA from left to right after 7 days at 25 °C in the dark; **B**. Top view of mature ascomata on OA; **C**. Side view of mature ascomata; **D**. Ascomata; **E**. Surface of ascomatal wall; **F**. Terminal ascomatal hairs; **G**. Asci; **H**. Ascospores. Scale bars: 50 μm (**D**); 10 μm (**E, F**); 20 μm (**G, H**).

*Chaetomium confusum* Van Warmelo, Mycologia 58 (6): 846 (1966).

*Chaetomium humicola* Van Warmelo, Mycologia 58 (6): 849 (1966).

*Chaetomium rubrogenum* Van Warmelo, Mycologia 58 (6): 852 (1966).

*Arcopilus globulus* Raza & L. Cai, Fungal Diversity 99: 75 (2019).

##### Typification.

USA • New England, from paper, 1912, Thaxter (***holotype*** BPI 1100494). Representative strain (CBS 153.52) designated by Wang et al. (2016a).

##### Description.

See Wang et al. (2016a).

##### Specimens examined.

BRAZIL • Minas Gerais: Viçosa, Zona Rural, from roots of *Habenaria petalodes*, April 2023, O.L. Pereira, D.O. Ramos, and P.T.S. Nogueira (living cultures COAD 3720 and COAD 3721); Conceição do Mato Dentro, Serra da Ferrugem, from roots of *Cattleya* sp., 10 April 2022, F.A. Custódio and D.O. Ramos (living cultures COAD 3718 and COAD 3719).

##### Distribution.

Brazil, Canada, China, Democratic Republic of the Congo, Europe, East Africa, India, Japan, Korea, USA.

##### Notes.

*Arcopilus aureus* is the type species of the genus and was originally described as *Chaetomium aureum* until Wang et al. (2016a) established the genus *Arcopilus*. This species presents superficial, ostiolate, and subglobose ascomata with arcuate terminal hairs and brown ascospores. Isolate COAD 3721 presented morphological characters similar to those of the type species. *Arcopilus aureus* has been reported as an endophyte in various plants, including *Ardisia mamillata* (Liang et al. 2024) and *Vitis vinifera* (Dwibedi and Saxena 2018), but it has never been isolated from orchids. Therefore, this is the first report of *A. aureus* as an endophyte of *Orchidaceae*.

#### Arcopilus cupreus

Taxon classificationFungiMonhysteridaChaetomiaceae

(Ames) X. Wei Wang & Samson, 2016

8D781ABD-E239-5EB5-A357-04A4F1BDAE71

Fig. 12

##### Synonyms.

*Chaetomium cupreum* L.M. Ames, Mycologia 41 (6): 642 (1949).

**Figure 12.**
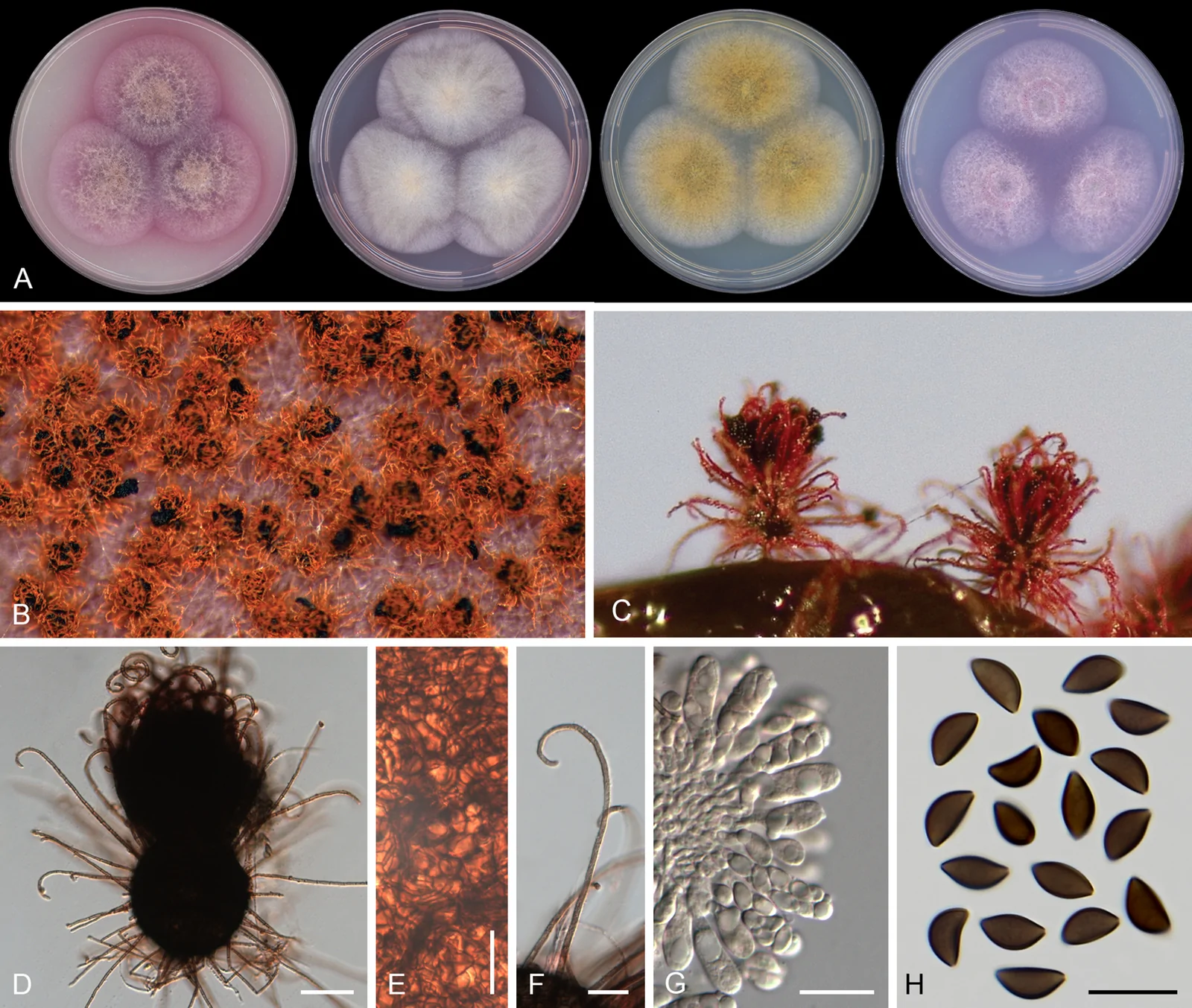
*Arcopilus cupreus*COAD 3722. **A**. Colonies on OA, CMA, MEA and PCA from left to right after 7 days at 25 °C in the dark; **B**. Top view of mature ascomata on OA; **C**. Side view of mature ascomata; **D**. Ascomata; **E**. Surface of ascomatal wall; **F**. Terminal ascomatal hair; **G**. Asci; **H**. Ascospores. Scale bars: 50 μm (**D**); 20 μm (**E–G**); 10 μm (**H**).

*Arcopilus tangerinicapillus* Raza & L. Cai, Fungal Diversity 99: 78 (2019).

*Arcopilus albae* Boonmee, Mapook & Phukhams, Phytotaxa 653 (2): 141 (2024), syn. nov.

##### Typification.

PANAMA • Canal Zone, from vegetable detritus, unknown date, Paul Marsh (***holotype*** BPI 580275). Representative strain (CBS 560.80) designated by Wang et al. (2016a).

##### Description.

See Ames (1949).

##### Specimens examined.

BRAZIL • Minas Gerais: Viçosa, from roots of *Cattleya* sp., 26 September 2012, O.L. Pereira (living culture COAD 3722).

##### Distribution.

Brazil, Canada, China, Panama, USA, Thailand.

##### Notes.

*Arcopilus cupreus* was first described as *Chaetomium cupreum* but was later transferred to *Arcopilus* by Wang et al. (2016a). This species is characterized by superficial, globose to ovoid ascomata, covered with bright, red-colored hairs, asci clavate, and olive-brown ascospores. These morphological characters could be observed in the isolate COAD 3722. In Brazil, *A. cupreus* was reported associated with the plant tissue of *Syagrus coronata*, a palm native to the Brazilian biome Caatinga (Fortes and Vitória 2022). This is the first report of *A. cupreus* as a root endophyte in *Orchidaceae* worldwide.

#### Arcopilus macrostiolatus

Taxon classificationFungiMonhysteridaChaetomiaceae

(Stchigel, K. Rodr. & Guarro) X. Wei Wang & Houbraken, 2022

463F4034-78BA-5623-B999-C130CBBCAFE6

##### Typification.

NIGERIA • Ihenyi Eha-Amufu: Isi-uzo, Enugu state, from forest soil, 12 July 1997, A. M. Stchigel (***holotype*** IMI 382896, ***isotype*** FMR 6780, culture **ex type** CBS 102435).

##### Description.

See Rodríguez et al. (2002).

##### Specimens examined.

BRAZIL • Minas Gerais: Santana do Riacho municipality, from roots of *Eulophia maculata*, 11 March 2024, D.O. Ramos (living cultures COAD 4168 and COAD 4169).

##### Distribution.

Brazil and Nigeria.

##### Notes.

This species was originally described as *Chaetomium macrostiolatum* by Rodríguez et al. (2002) based on a strain isolated from forest soil in Nigeria and reclassified to the genus *Arcopilus* by Wang et al. (2022b). Our isolate was identified through morphological analysis, exhibiting the diagnostic characteristics of the species: subglobose to ovoid ascomata with a remarkably broad ostiole and pale to mid-brown, limoniform, asymmetrical ascospores. This study represents the first global record of the species as a root endophyte in the *Orchidaceae* family, specifically in *E. maculata*.

#### Chaetomium

Taxon classificationFungiMonhysteridaChaetomiaceae

Kunze, 1817

203F3F3C-4F0E-573A-A6C1-25DAACCBCDC0

##### Type species.

*Chaetomium globosum* Kunze, 1817.

#### Chaetomium coarctatum

Taxon classificationFungiMonhysteridaChaetomiaceae

Sergeeva, 1961

68E657FE-31A6-5C8C-84DE-9E70B1FD9BB5

Fig. 13

##### Typification.

RUSSIA • St. Petersburg, from seed of *Campanula medium*, collector and collection date unknown, isolated by K.S. Sergejeva (culture ***ex-type*** CBS 162.62).

**Figure 13.**
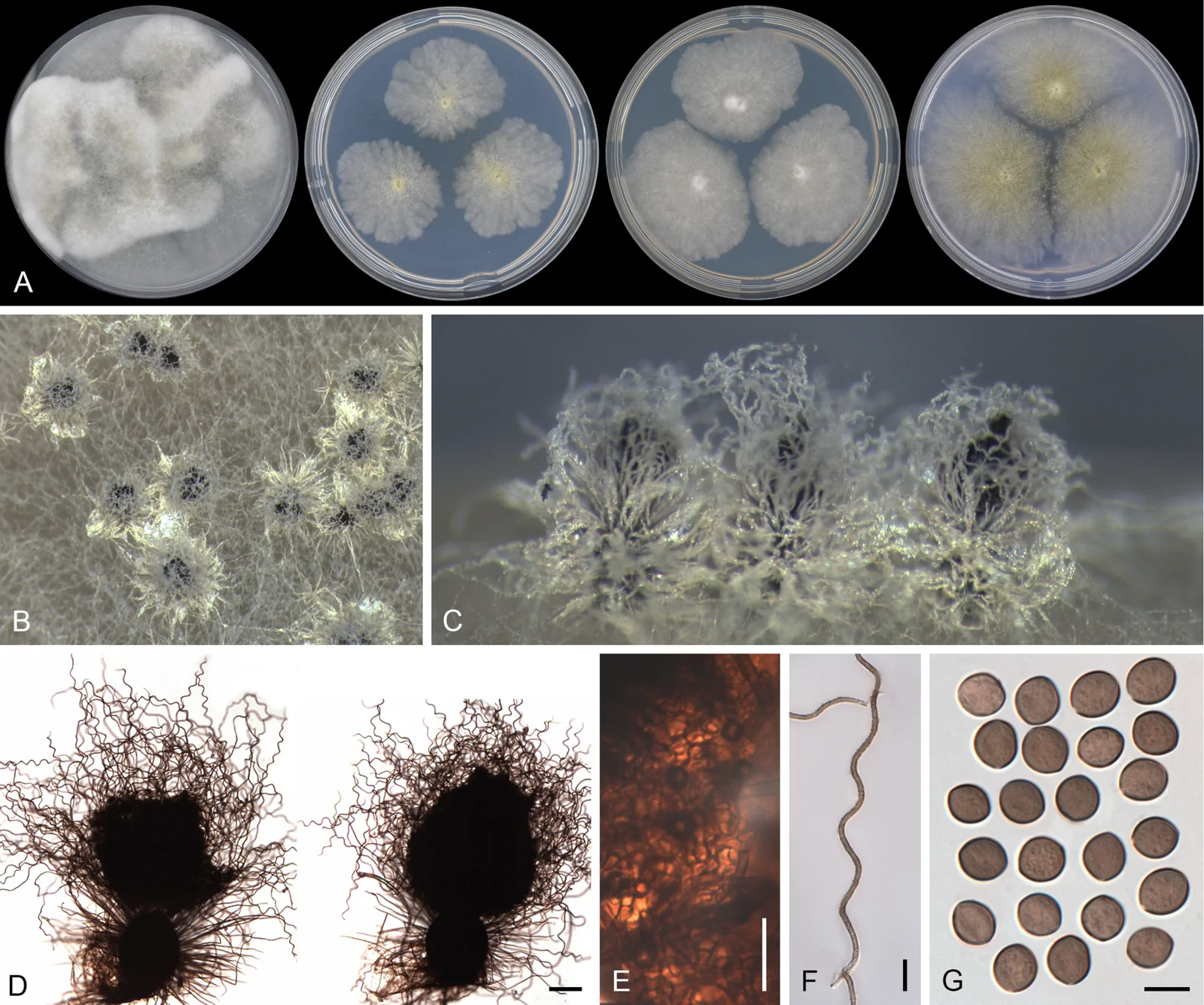
*Chaetomium coarctatum*COAD 3729. **A**. Colonies on OA, CMA, MEA and PCA from left to right after 7 days at 25 °C in the dark; **B**. Top view of mature ascomata on OA; **C**. Side view of mature ascomata; **D**. Ascomata; **E**. Surface of ascomatal wall; **F**. Terminal ascomatal hair; **G**. Ascospores. Scale bars: 100 μm (**D**); 20 μm (**E, F**); 10 μm (**G**).

##### Description.

See Wang et al. (2016b).

##### Specimens examined.

BRAZIL • Rio de Janeiro: Nova Friburgo, Córrego D’Antas, from roots of *Cyclopogon congestus*, 20 July 2023, D.O. Ramos (living culture COAD 3729).

##### Distribution.

Brazil, China, and Russia.

##### Notes.

*Chaetomium coarctatum* was first described on *Campanula medium* seeds. Subsequently, it was considered a synonym of *Chaetomium globosum*, but phylogenetic analyses confirmed it as a legitimate species (Wang et al. 2016b). *Chaetomium coarctatum* was reported as root endophyte in *Avena fatua* (Bouzouina et al. 2021). To the best of our knowledge, this is the first report of an endophytic association between *C. coarctatum* and an *Orchidaceae* plant and the first report of this species in Brazil.

#### Chaetomium cochliodes

Taxon classificationFungiMonhysteridaChaetomiaceae

Palliser, 1910

506E0AEF-A35A-5626-B8CB-48D1099B7048

Fig. 14

##### Synonyms.

*Chaetomium concavisporum* Raza & L. Cai, Fungal Diversity 99: 80 (2019).

**Figure 14.**
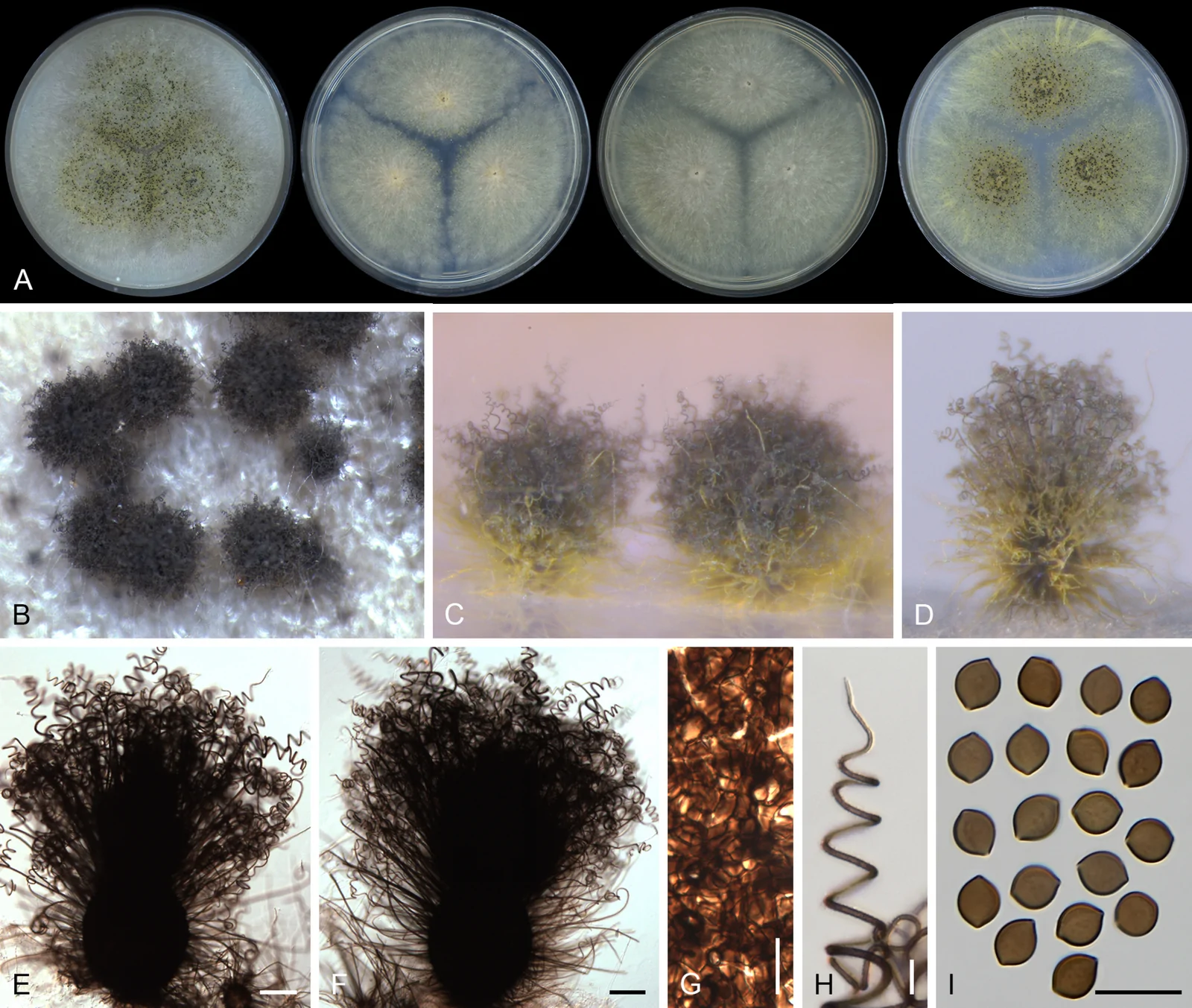
*Chaetomium cochliodes*COAD 3731. **A**. Colonies on OA, CMA, MEA and PCA from left to right after 7 days at 25 °C in the dark; **B**. Top view of mature ascomata on OA; **C, D**. Side view of mature ascomata; **E, F**. Ascomata; **G**. Surface of ascomatal wall; **H**. Terminal ascomatal hair; **I**. Ascospores. Scale bars: 100 μm (**E, F**); 20 μm (**G, H**); 10 μm (**I**).

#### Chaetomium ovatoascomatis

Taxon classificationFungiMonhysteridaChaetomiaceae

Raza & L. Cai, Fungal Diversity 99: 82 (2019)

568D015B-25AD-5678-B9A5-5FD6FD2C26AE

##### Typification.

USA • Newfield: New Jersey, on old paper exposed to the weather, October 1880 (***holotype*** New York Botanical Garden Specimen ID 01050405). **Epitype** HMAS 244354 and culture ***ex-epitype*** CBS 155.52 designated by Wang et al. (2016b) from the same country and locality as the holotype.

##### Description.

See Wang et al. (2016b).

##### Specimens examined.

BRAZIL • Rio de Janeiro: Nova Friburgo, Córrego D’Antas, from roots of *Eulophia maculata*, 01 October 2022, D.O. Ramos (living cultures COAD 3730 and COAD 3731); Minas Gerais: Araponga, from roots of *Zygopetalum mackayi*, 21 November 2021, O.L. Pereira (living cultures COAD 3760 and COAD 3761).

##### Distribution.

Brazil, China, and USA.

##### Notes.

*Chaetomium cochliodes* was introduced by Palliser (1910) but was later treated as a synonym of *C. globosum* by von Arx et al. (1986). *Chaetomium cochliodes* differs from other *Chaetomium* species by its distinctive coiled ascomatal hairs. This species was reintroduced by Wang et al. (2016b) based on phylogenetic analyses. The isolate COAD 3731 produced pure yellow lateral hairs, in contrast to the greenish olivaceous terminal hairs and rosy vinaceous soluble pigment on OA produced by the ex-epitype CBS 155.52 (Wang et al. 2016b). This species can endophytically colonize the root tissues of barley, soybean, wheat, and other plants used in agriculture (Kopylov et al. 2021). Nonetheless, *C. cochliodes* has also been reported in goat dung in Brazil (Melo et al. 2019). To the best of our knowledge, this is the first report of *C. cochliodes* as an endophyte in *Orchidaceae*.

#### Chaetomium globosum

Taxon classificationFungiMonhysteridaChaetomiaceae

Kunze, 1817

6059BFA2-6057-5EC7-9D62-6C5A2EB252F0

Fig. 15

##### Synonyms.

*Chaetomium affine* Corda, Icones fungorum hucusque cognitorum 4: 37, table 8, fig. 101 (1840).

**Figure 15.**
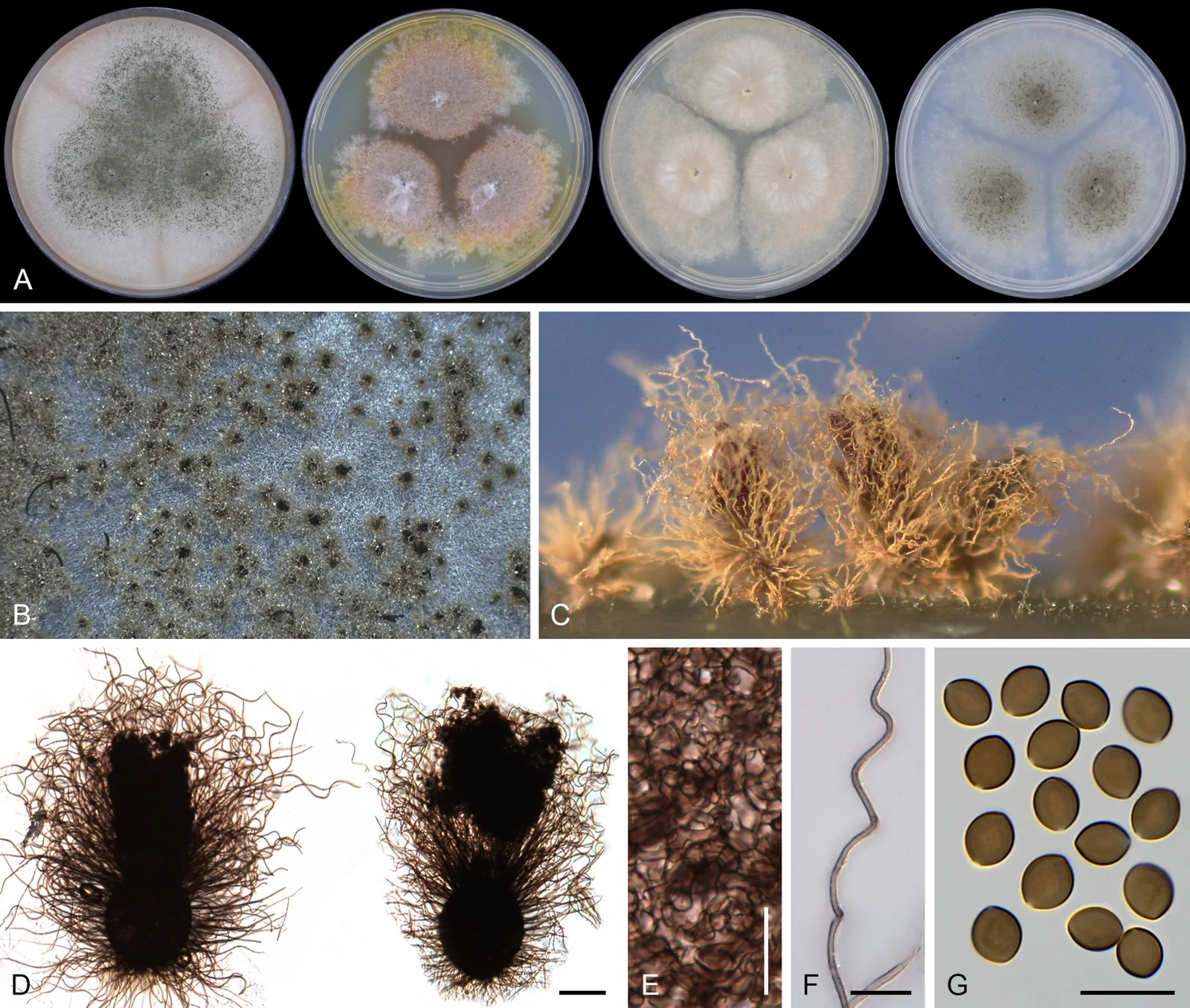
*Chaetomium globosum*COAD 3732. **A**. Colonies on OA, CMA, MEA and PCA from left to right after 7 days at 25 °C in the dark; **B**. Top view of mature ascomata on OA; **C**. Side view of mature ascomata; **D**. Ascomata; **E**. Surface of ascomatal wall; **F**. Terminal ascomatal hair; **G**. Ascospores. Scale bars: 100 μm (**D**); 20 μm (**E, F**); 10 μm (**G**).

*Chaetomium anastomosans* Raza & L. Cai, Fungal Diversity 99: 78 (2019).

*Chaetomidium barbatum* Traaen, Nytt Mag. Naturvidensk. 52: 19 (1914).

*Chaetomium fibripilium* L.M. Ames, Mycologia 42 (5): 642 (1950).

*Chaetomium kunzeanum*Zopf (1881).

*Chaetomium lusitanicum* M.R.M. Gomes, Estudos e Informação: 3 (1953).

*Chaetomium mollipilium* L.M. Ames, Mycologia 42 (5): 644 (1950).

*Chaetomium ochraceum* Tschudy, Amer. J. Bot. 24: 472 (1937).

*Chaetomium olivaceum* Cooke & Ellis, Grevillea 6 (39): 96, Pl. 100, fig. 38 (1878).

*Chaetomium rectum* Sergeeva, Not. syst. Pl. non-vasc.: 143 (1961).

*Chaetomium spirale* Zopf, Nova Acta Acad. Caes. Leop.-Carol. German. Nat. Cur. 42 (5): 275 (1881).

*Chaetomium subglobosum* Sergeeva, Bot. Mater. Otd. Sporov. Rast. Bot. Inst. Komarova Akad. Nauk S.S.S.R. 13: 172 (1960).

*Chaetomium subterraneum* Swift & Povah, 1929.

##### Typification.

GERMANY • Leipzig, from a dry culture that was collected in the same locality as the holotype (**neotype** CBS H-22185, **ex-neotype** CBS 160.62, designated by Wang et al. (2016b)).

##### Description.

See Wang et al. (2016b).

##### Specimens examined.

BRAZIL • Minas Gerais: Viçosa, Recanto das Cigarras-UFV, from roots of *Eulophia maculata*, 29 September 2019, D.O. Ramos (living culture COAD 3732); Araponga, from roots of *Zygopetalum mackayi*, 21 November 2021, O.L. Pereira (living culture COAD 3762); Viçosa, Universidade Federal de Viçosa, from roots of *Polystachya concreta*, 13 May 2022, D.O. Ramos and P.T.S. Nogueira (living culture COAD 3759).

##### Distribution.

Africa, Asia, Europe, North America, and South America.

##### Notes.

*Chaetomium globosum* is the type species of the genus and presents diverse morphological variations in exudate colors and ascomatal hairs among strains isolated from different substrates (Wang et al. 2016b). Isolates COAD 3732 and COAD 3762 were grouped next to the type of *C. globosum*, whereas COAD 3759 was grouped near to the isolates described as *C. anastomosans* but that were synonymized into *C. globosum* by Wang et al. (2022b).

The isolate COAD 3732 produced intense sporulation with salmon to apricot soluble pigment on OA and pale luteous to brick soluble pigment on CMA. In Brazil, *C. globosum* has been isolated from the leaf litter of *Syagrus coronata* and as a root endophyte of *Musa acuminata* (Fortes and Vitória 2022; dos Santos Santana et al. 2026). In addition, this species has already been reported as an endophyte of *Orchidaceae* (Adit et al. 2022). Nonetheless, this is the first report of *C. globosum* as an endophyte of *E. maculata*, *Pol. concreta* and *Zygopetalum mackayi*.

#### Chaetomium pseudocochliodes

Taxon classificationFungiMonhysteridaChaetomiaceae

X. Wei Wang, X.Z. Liu & Crous, 2016

E90C069E-DA6B-59E8-8633-C49B46AB2203

Fig. 16

##### Typification.

CHINA • Yunnan Province: Wenshan County, from fibrous root of *Panax notoginseng*, 10 April 2003, X.-Z Liu (***holotype*** HMAS 244435, ***isotype*** CBS H-22197, culture ***ex-type*** CGMCC 3.9441).

**Figure 16.**
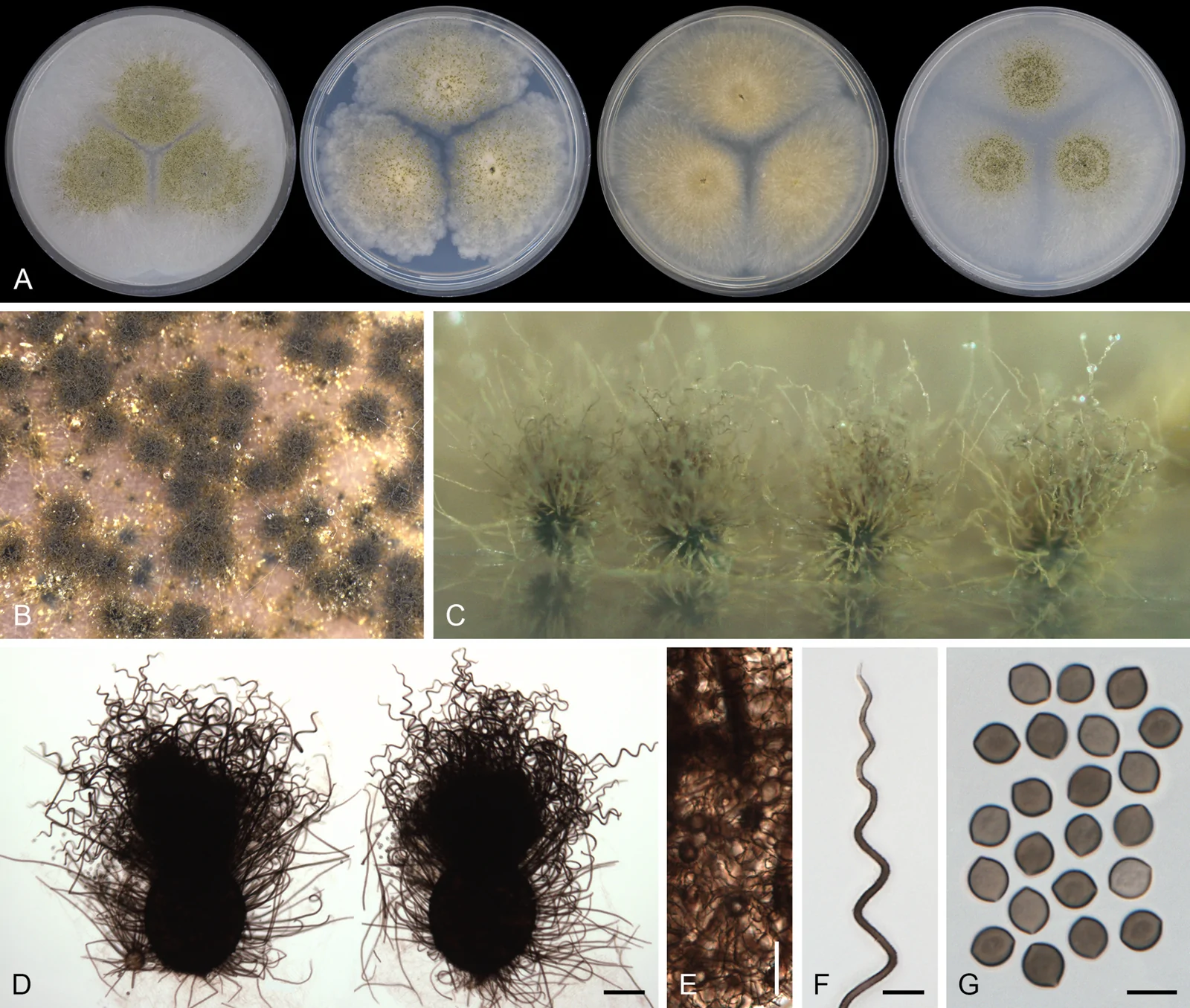
*Chaetomium pseudocochliodes*COAD 3733. **A**. Colonies on OA, CMA, MEA and PCA from left to right after 7 days at 25 °C in the dark; **B**. Top view of mature ascomata on OA; **C**. Side view of mature ascomata; **D**. Ascomata; **E**. Surface of ascomatal wall; **F**. Terminal ascomatal hair; **G**. Ascospores. Scale bars: 100 μm (**D**); 20 μm (**E, F**); 10 μm (**G**).

##### Description.

See Wang et al. (2016b).

##### Specimens examined.

BRAZIL • Minas Gerais: Viçosa, Recanto das Cigarras-UFV, from roots of *Polystachya concreta*, 13 May 2022, D.O. Ramos and P.T.S. Nogueira (living culture COAD 3733); Araponga, isolated from roots of *Gomesa recurva*, 10 August 2022, D.O. Ramos (living cultures COAD 3734 and COAD 3735).

##### Distribution.

Brazil and China.

##### Notes.

*Chaetomium pseudocochliodes* is phylogenetically related to *C. cochliodes* but differs from this species by the irregular ascomatal hairs and shape of ascospores with more protruding ends (Wang et al. 2016b). The isolate COAD 3733 differs from the ex-type culture *C. cochliodes* CGMCC 3.9441 in the absence of exudates, and terminal ascomatal hairs of type I were more abundant than type II. This is the first report of *C. pseudocochliodes* in Brazil and as an endophyte of *Orchidaceae*.

#### Chaetomium tenue

Taxon classificationFungiMonhysteridaChaetomiaceae

X. Wei Wang, Crous & L. Lombard, 2016

176AC334-9EC1-5D8D-A29D-22B313A4F1A0

Fig. 17

##### Typification.

Unknown, no collection information, deposited in CBS by A.L. McAulay, August 1938 (***holotype*** CBS H-22195, culture ***ex-type*** CBS 139.38).

**Figure 17.**
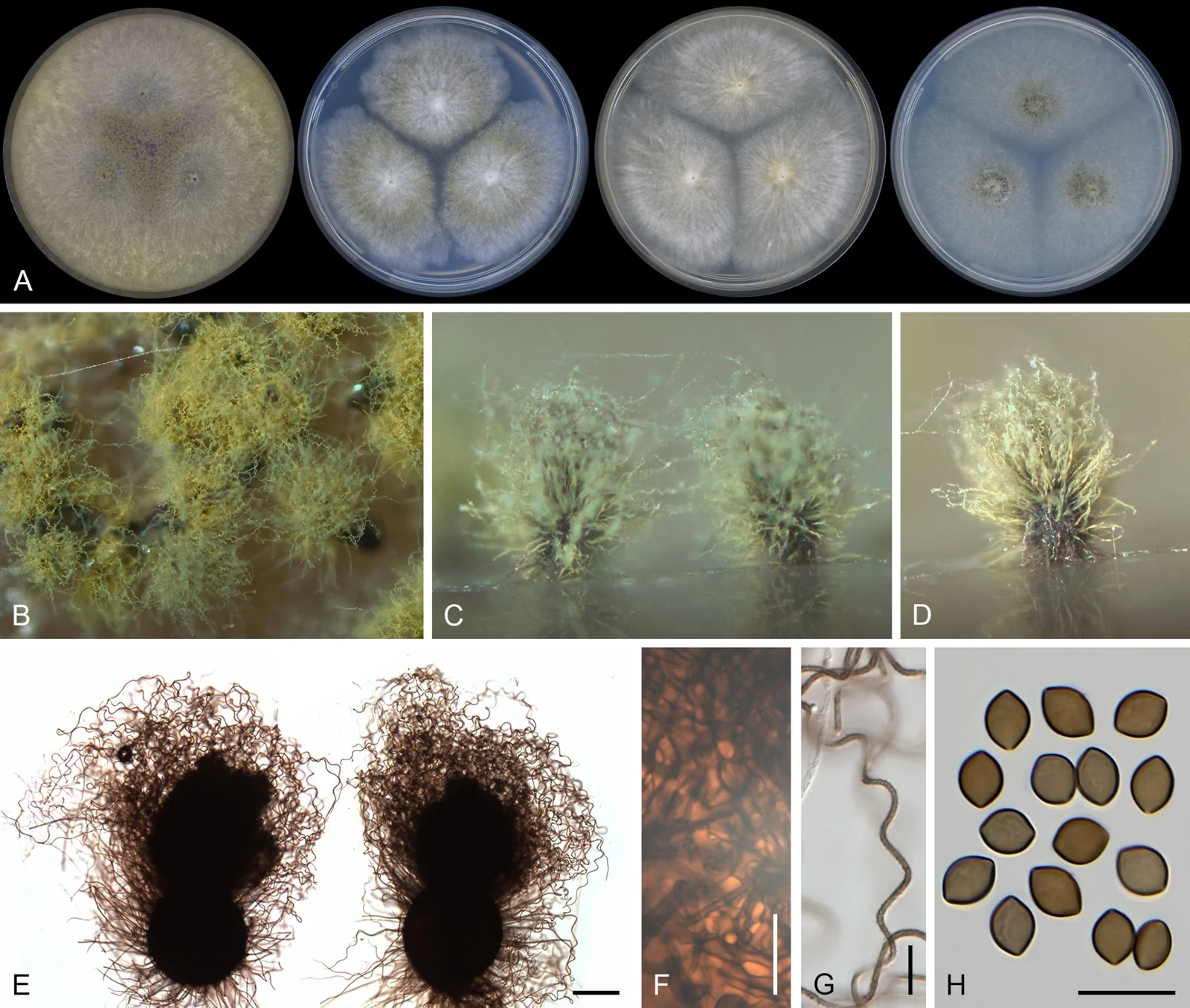
*Chaetomium tenue*COAD 3736. **A**. Colonies on OA, CMA, MEA and PCA from left to right after 7 days at 25 °C in the dark; **B**. Top view of mature ascomata on OA; **C, D**. Side view of mature ascomata; **E**. Ascomata; **F**. Surface of ascomatal wall; **G**. Terminal ascomatal hair; **H**. Ascospores. Scale bars: 100 μm (**E**); 20 μm (**F, G**); 10 μm (**H**).

##### Description.

See Wang et al. (2016b).

##### Specimens examined.

BRAZIL • Minas Gerais: Viçosa, Recanto das Cigarras-UFV, from roots of *Eulophia maculata*, 29 September 2019, D.O. Ramos (living culture COAD 3736).

##### Distribution.

As the location of the type specimen is unknown, the only exact location so far is in Brazil.

##### Notes.

Phylogenetic analyses grouped the isolate COAD 3736 with the ex-type (CBS 139.38) of the species. *Chaetomium tenue* is phylogenetically related to *C. camelliae* and *C. pseudoglobosum*. The isolate obtained in this study differs from the ex-type culture by abundant production of an orange soluble pigment on OA. Other morphological characters, such as ascomata, terminal hairs, asci, and ascospores are similar to those described by Wang et al. (2016b). This the first report of *C. tenue* in Brazil and as an endophyte in *Orchidaceae* worldwide.

#### Collariella

Taxon classificationFungiMonhysteridaChaetomiaceae

X. Wei Wang & Samson, 2016

4C404C56-CE09-582D-82BA-78A1B2DFE993

##### Type species.

*Collariella bostrychodes* (Zopf) X. Wei Wang & Samson, 2016.

#### Collariella fluminensis

Taxon classificationFungiMonhysteridaChaetomiaceae

D.O. Ramos, P.T.S. Nogueira & O.L. Pereira sp. nov.

989C2ED1-F087-5B71-A2A7-4D36E393C4DC

862444

Fig. 18

##### Etymology.

The name *Fluminense* refers to people born in the state of Rio de Janeiro, where the fungus was collected.

**Figure 18.**
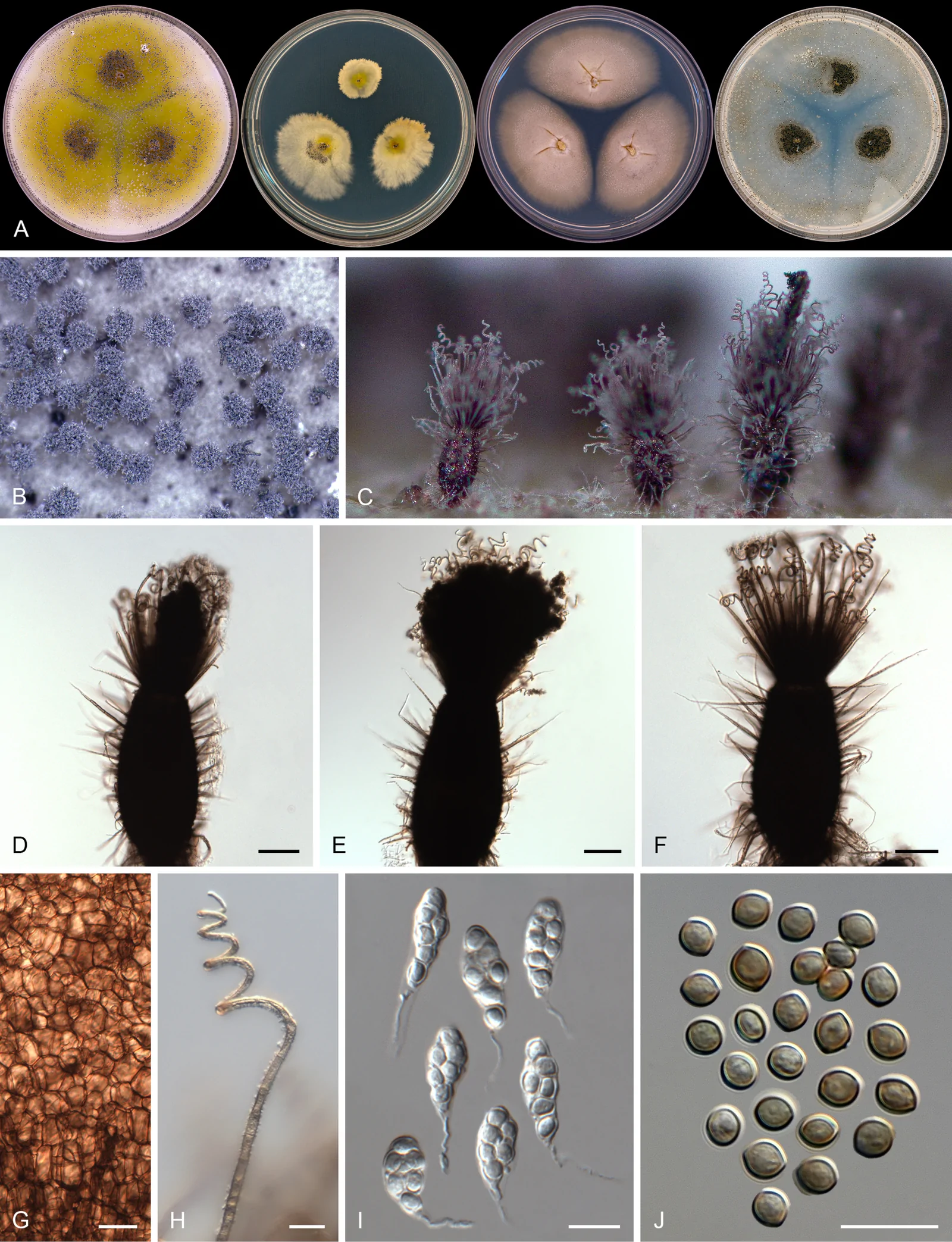
*Collariella fluminensis*COAD 3737, ex-type. **A**. Colonies on OA, CMA, MEA and PCA from left to right after 4 weeks at 25 °C in the dark; **B**. Top view of mature ascomata on OA; **C**. Side view of mature ascomata; **D–F**. Ascomata; **G**. Surface of ascomatal wall; **H**. Terminal ascomatal hair; **I**. Asci; **J**. Ascospores. Scale bars: 100 μm (**D–F**); 20 μm (**G, H**); 10 μm (**I, J**).

##### Typification.

BRAZIL • Rio de Janeiro: Nova Friburgo, Corrego D’antas, from roots of *Cyclopogon congestus*, 17 July 2023, D.O. Ramos (***holotype*** VIC 49500 preserved as dried culture, culture ***ex-type***COAD 3737).

##### Description.

***Ascomata*** superficial, pale olivaceous gray in reflected light owing to the ascomatal hairs, ostiolate, ovoid to ellipsoid, 393–552 μm high, 205–252 μm diam. ***Ascomatal wall*** brown, ***textura angularis*** in surface view. ***Terminal hairs*** arising from the apical collar, conspicuously rough, dark brown, septate, erect in the lower part, spirally coiled in the upper part. ***Lateral hairs*** seta-like, tapering and fading towards the tips. ***Asci*** fasciculate, fusiform or clavate, spore-bearing portion 16.5–26 × 8–11 μm, stalks 8.5–18 μm long, with 8 irregularly-arranged ascospores, evanescent. ***Ascospores*** pale brown when mature, limoniform, bilaterally flattened, (5.5–)6–6.5 × (4.5–)5–5.5(–6) μm in face view, with one apical germ pore. ***Asexual morph*** unknown.

***Culture characteristics***: Colonies on OA attaining 32–36 mm diameter after 7 days at 25 °C, edge entire, aerial mycelium absent, flat, circular shape; reverse luteous due to the intense production of soluble pigment. Colonies on CMA attaining 23–27 mm diameter after 7 days at 25 °C, entire margin, aerial mycelium absent, flat, circular to irregular shape; reverse pale luteous. Colonies on MEA attaining 26–30 mm diameter after 7 days at 25 °C, edge entire, aerial mycelium absent, flat, irregular shape; reverse pale luteous; soluble pigments absent. Colonies on PCA attaining 30–32 mm diameter after 7 days at 25 °C, edge entire, aerial mycelium absent, flat, circular; reverse buff; soluble pigments absent.

##### Additional specimens examined.

BRAZIL • Minas Gerais: Viçosa, Mata do Paraíso, from roots of *Eulophia maculata*, 31 August 2023, D.O. Ramos (living cultures COAD 3738 and COAD 3739).

##### Distribution.

Brazil (Atlantic rainforest in Rio de Janeiro state and Seasonal Semideciduous Forest within the Atlantic Forest domain in Minas Gerais state).

##### Notes.

*Collariella fluminensis* clustered in a well-supported clade phylogenetically related to *Col. capillicompacta*, *Col. carteri*, *Col. pachypodioides*, and *Col. hongheensis* (Fig. 2). Morphologically, *Col. fluminensis* can be distinguished from *Col. carteri* by its larger ascomata (393–552 × 205–252 *vs*. 175–320 × 110–220 μm), terminal hairs (verrucose and spirally coiled *vs*. smooth and seta-like in *Col. carteri*), and smaller ascospores ((5.5–)6–6.5 × (4.5–)5–5.5(–6) *vs*. (6–)6.5–7.5(–8.5) × 5–6(–7.5) μm). Additionally, *Col. fluminensis* shows higher growth on OA (32–36 *vs*.24–30 mm in *Col. carteri*) and slightly reduced growth on MEA (26–30 *vs*.29–33 mm) (Wang et al. 2016a). In comparison to *Col. capillicompacta*, *Col. fluminensis* has larger ascomata (393–552 × 205–252 *vs*.198.9–303.6 × 146.6–230.3 μm), and slightly larger ascospores ((5.5–)6–6.5 × (4.5–)5–5.5(–6) *vs*. 5.2–6.2 × 4.2–5.2 μm) (Aghyl et al. 2021). *Collariella fluminensis* can be differentiated from *Col. pachypodioides* by its larger ascomata (393–552 μm *vs*. 350–460 μm) and spirally coiled and verrucose terminal hairs, whereas in *Col. pachypodioides*, its terminal hairs are smooth to slightly roughened (Ames 1961). Compared to *Col. hongheensis*, *Col. fluminensis* can be distinguished by its slightly larger ascomata (393–552 × 205–252 *vs*. 250–500 × 200–300), smaller asci (16.5–26 × 8–11 *vs*. 30–63.5 × 5–13 μm), and lacks hamathecium inside the ascomata (Manawasinghe et al. 2024).

#### Dichotomopilus

Taxon classificationFungiMonhysteridaChaetomiaceae

X. Wei Wang & Samson, 2016

71784974-DF15-5A9C-914C-3347632F29AE

##### Type species.

*Dichotomopilus indicus* (Corda) X. Wei Wang & Samson, 2016.

#### Dichotomopilus brasiliensis

Taxon classificationFungiMonhysteridaChaetomiaceae

D.O. Ramos, P.T.S. Nogueira & O.L. Pereira sp. nov.

59E49011-9154-578F-862B-AFE5D3D3DC20

862445

Fig. 19

##### Etymology.

Refers to Brazil, the country where the isolates used in the description were collected.

**Figure 19.**
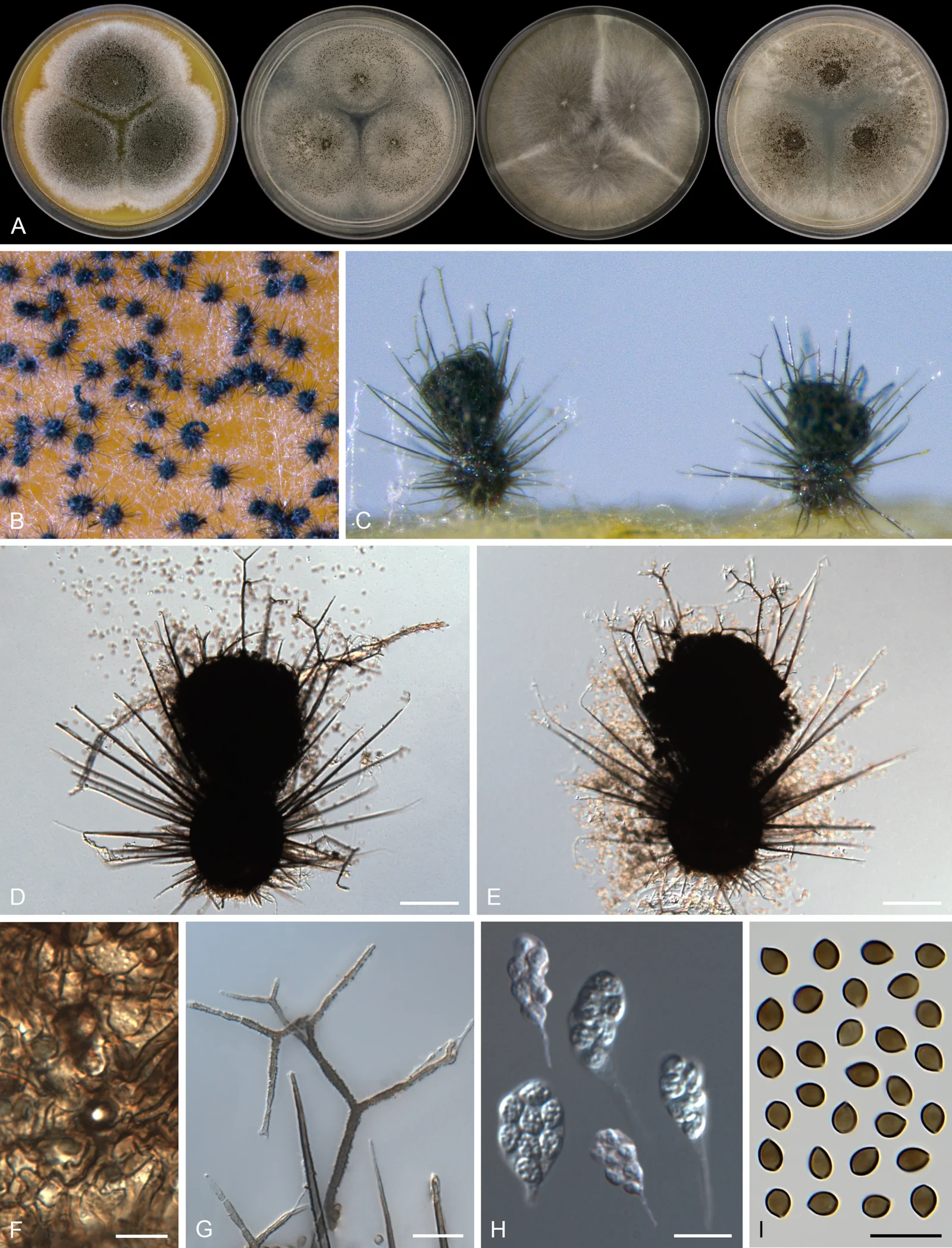
*Dichotomopilus brasiliensis*COAD 3741, ex-type. **A**. Colonies on OA, CMA, MEA and PCA from left to right after 3 weeks at 25 °C in the dark; **B**. Top view of mature ascomata on OA; **C**. Side view of mature ascomata; **D, E**. Ascomata; **F**. Surface of ascomatal wall; **G**. Terminal ascomatal hairs; **H**. Asci; **I**. Ascospores. Scale bars: 100 μm (**D, E**); 20 μm (**G**); 10 μm (**F, H, I**).

##### Typification.

BRAZIL • Minas Gerais: Araponga, from roots of *Zygopetalum mackayi*, 21 November 2021, O.L. Pereira (***holotype*** VIC 49501 preserved as dried culture, culture ***ex-type***COAD 3741).

##### Description.

***Ascomata*** superficial, ostiolate, dark gray in reflected light, subglobose to ellipsoid, 160–220 μm high, 131–201 μm diam. ***Ascomatal wall*** brown, ***textura intricata*** or ***angularis***. ***Terminal hairs*** dark brown, septate, unbranched or dichotomously branched 1–3 times towards the apex. ***Lateral hairs*** seta-like, tapering and fading towards tips. ***Asci*** fasciculate, clavate, with 8 ascospores irregularly arranged, with spore-bearing portion 13–19 × 7–12 μm, stalks 6–15.5 μm long, evanescent. ***Ascospores*** brown, ovoid to slightly elongate-ovoid, bilaterally flattened, (4.5–) 5–6 × 3.5–4.5 (–5.5) μm in face view, with one apical germ. ***Asexual morph*** unknown.

***Culture characteristics***: Colonies on OA attaining 35–37 mm diameter after 7 days at 25 °C, edge entire to undulate, white aerial mycelium sparse, flat, circular; reverse pale luteous to luteous owing to the presence of soluble pigment. Colonies on CMA attaining 47–60 mm diameter after 7 days at 25 °C, edge entire to undulate, white aerial mycelium moderate, flat, circular; reverse buff; soluble pigment absent. Colonies on MEA reaching over 75 mm diameter after 7 days at 25 °C, edge entire, white aerial mycelium dense, flat, circular; reverse pale luteous due to the presence of soluble pigments. Colonies on PCA attaining 59–75 mm diameter after 7 days at 25 °C, edge entire, white aerial mycelium sparse to moderate, flat, circular; reverse buff to pale luteous due to production soluble pigment.

##### Additional specimens examined.

BRAZIL • Maranhão: Itinga do Maranhão municipality, from roots of *Musa* sp., 10 December 2021, F.A. Custódio (living culture COAD 4293); Bahia: Vale do Juliana, from roots of *Theobroma cacao*, 18 July 2025, J.S. Santana and F.A. Custódio (living culture COAD 4294).

##### Distribution.

Brazil (Atlantic rainforest in Bahia state, Amazon rainforest in Maranhão state, and Rupestrian grassland within the Atlantic forest in Minas Gerais state).

##### Notes.

*Dichotomopilus brasiliensis* was clustered in a unique lineage that was phylogenetically sister to *D. subfunicola* (Fig. 4). When compared with the description by Wang et al. (2016a), ascospores are slightly shorter ((4.5–)5–6 *vs*. 5.5–6.5(–7.5) μm) and brown instead of olivaceous. The ascomatal wall of *D. brasiliensis* is composed of ***textura intricata*** or ***angularis***, whereas that of *D. subfunicola* is composed of ***textura intricata*** or ***epidermoidea***. Additionally, *D. brasiliensis* exhibited higher growth rates than *D. subfunicola* on different culture media: OA (35–37 *vs*. 25–28 mm), MEA (over 75 *vs*. 41–47 mm), and PCA (59–75 *vs*. 55–62 mm).

#### Dichotomopilus variostiolatus

Taxon classificationFungiMonhysteridaChaetomiaceae

(A. Carter) X. Wei Wang & Samson, 2016

2C244724-E899-5850-AF72-85B4F47065DB

Fig. 20

##### Synonym.

*Chaetomium variostiolatum* A. Carter, Canad. J. Bot. 61 (10): 2603 (1983).

**Figure 20.**
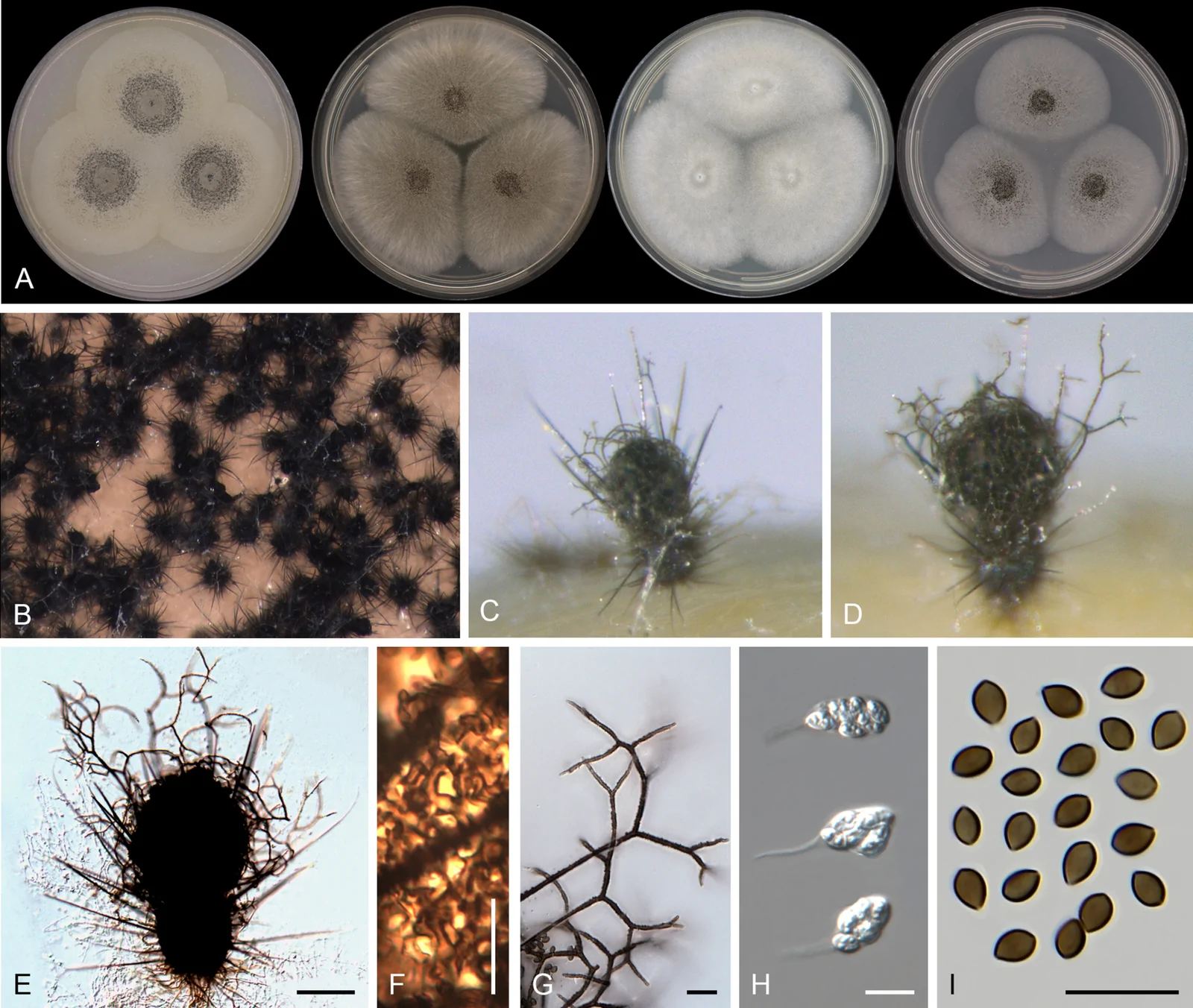
*Dichotomopilus variostiolatus*COAD 3740. **A**. Colonies on OA, CMA, MEA and PCA from left to right after 4 weeks at 25 °C in the dark; **B**. Top view of mature ascomata on OA; **C, D**. Side view of mature ascomata; **E**. Ascomata; **F**. Surface of ascomatal wall; **G**. Terminal ascomatal hairs; **H**. Asci; **I**. Ascospores. Scale bars: 100 μm (**E**); 20 μm (**F, G**); 10 μm (**H, I**).

##### Typification.

NEW GUINEA • from tarpaulin, 14 September 1944, E.T. Reese (***holotype*** QM 36d) deposited as dried culture in the University of Toronto Cryptogamic Herbarium (TRTC). Wang et al. (2016a) designated the ***ex-type*** culture CBS 179.84 for molecular analysis.

##### Description.

See Carter (1983).

##### Specimens examined.

BRAZIL • Goiás: Goiânia, from roots of *Cattleya nobilior*, 05 January 2023, P.T.S. Nogueira (living culture COAD 3740); Minas Gerais: Viçosa, isolated from roots of *Cattleya* sp., 2012, O.L. Pereira (living culture COAD 3763).

##### Distribution.

Brazil, New Guinea, USA, Thailand.

##### Notes.

This species was first introduced as *Chaetomium variostiolatum* by Carter (1983) and then combined with *Dichotomopilus variostiolatus* by Wang et al. (2016a) when the genus was established. This species produces unbranched seta-like or dichotomously branched terminal hairs. Both isolates in this study produced two types of terminal hairs, similar to those observed by Wang et al. (2016a). Recently, dos Santos Santana et al. (2026) isolated *D. variostiolatus* as a root endophyte of *Musa acuminata* in Brazil. In the present study, *D. variostiolatus* was found endophytically on roots of *Cattleya nobilior* and *Cattleya* sp., suggesting that this species may be a common endophyte in the roots of Brazilian plants. Moreover, this is the first report of *D. variostiolatus* as an endophyte in *Orchidaceae*.

#### Humicola

Taxon classificationFungiMonhysteridaChaetomiaceae

Traaen, 1914

33BAD679-2D33-5710-B16E-7F2D0334A717

##### Type species.

*Humicola fuscoatra* Traaen, 1914.

#### Humicola capillata

Taxon classificationFungiMonhysteridaChaetomiaceae

D.O. Ramos, P.T.S. Nogueira & O.L. Pereira sp. nov.

99788E48-1375-58C8-94B1-AD2D8AB0D1D9

862446

Fig. 21

##### Etymology.

From the Latin *capillatus*, which means covered with hair, due to the abundance of ascomatal hairs.

**Figure 21.**
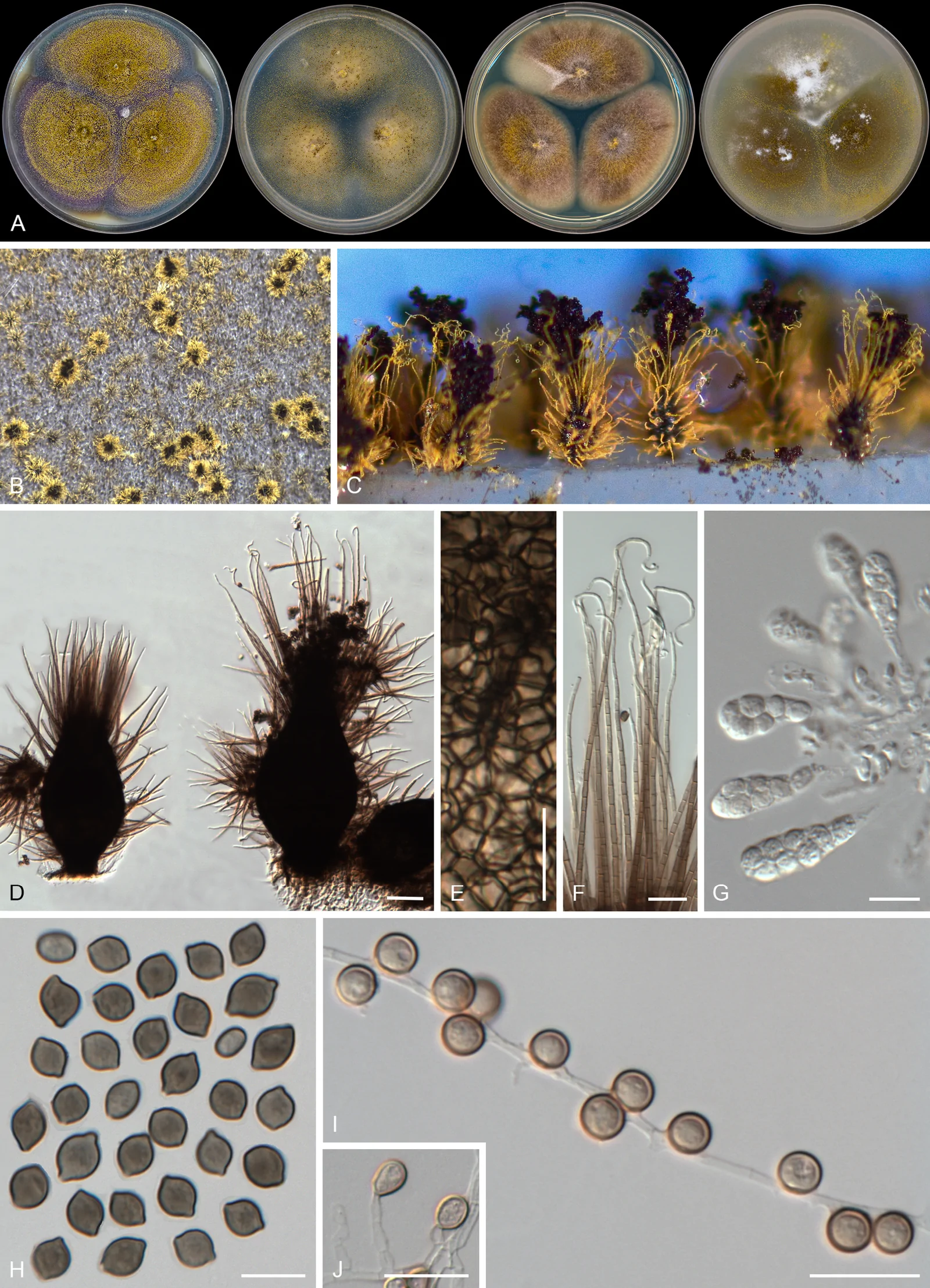
*Humicola capillata*COAD 3744, ex-type. **A**. Colonies on OA, CMA, MEA and PCA from left to right after 4 weeks at 25 °C in the dark; **B**. Top view of mature ascomata on OA; **C**. Side view of mature ascomata; **D**. Ascomata; **E**. Surface of ascomatal wall; **F**. Terminal ascomatal hairs; **G**. Asci; **H**. Ascospores; **I, J**. Aleurioconidia-like and hyphae. Scale bars: 50 μm (**D**); 20 μm (**E, F, I, J**); 10 μm (**G, H**).

##### Type.

BRAZIL • Distrito Federal: Brasília, isolated as root endophyte on *Eulophia maculata*, 08 August 2023, D.O. Ramos and P.T.S. Nogueira (***holotype*** VIC 49507 preserved as dried culture, culture ***ex-type***COAD 3744).

##### Description.

***Ascomata*** superficial, ostiolate, luteous to amber in reflected light owing to ascomatal hairs, ovoid to elongate-obpyriform, apically attenuated to an elongate conical or short cylindrical neck, 273–339.5 μm high, 131.5–173 μm diam. at the widest part. ***Ascomatal wall*** brown, composed of ***textura angularis***. ***Terminal hairs*** arising from the extension of the adjacent ostiolar cells, seta-like with delicate hamete tip, smooth. ***Lateral hairs*** are similar to terminal ones, tapering and fading towards the tips. ***Asci*** clavate, with spore-bearing portion 17–25.5 × 6.5–10.5 μm, stalks 8–12.5 μm long, with 8 biseriate ascospores, evanescent. ***Ascospores*** olivaceous, limoniform, umbonate at both ends, bilaterally flattened, (7.5–)8–8.5(–10) × (5.5–)6–6.5(–7) μm in face view, with one apical germ pore. ***Somatic hyphae*** hyaline. ***Aleurioconidia-like conidia*** produced laterally on hyphae or on branches, solitary or in small clusters of two or more, subglobose or occasionally pyriform, aseptate, brown to pale brown, thick-walled when mature, 7–8 × (6.5–)7–8(–8.5) μm.

***Culture characteristics***: Colonies on OA attaining 32–34 mm diameter after 7 days at 25 °C, edge entire, aerial mycelium absent, flat, circular; reverse ochreous at the center of the colony and pale vinaceous to livid vinaceous due to pigment production. Colonies on CMA attaining 28–32 mm diameter after 7 days at 25 °C, entire margin, white aerial mycelium sparse, flat, irregular shape; reverse buff to pale luteous; soluble pigment absent. Colonies on MEA attaining 33–37 mm diameter after 7 days at 25 °C, edge entire, white aerial mycelium with hypha tuffs, flat, circular; reverse cinnamon at the center and buff at the margins; soluble pigments absent. Colonies on PCA attaining 30–33 mm diameter after 7 days at 25 °C, edge entire, white aerial mycelium moderate with mycelial strands, flat, circular; reverse buff to honey; soluble pigments absent.

##### Distribution.

Brazil (Gallery Forest within the Cerrado biome in the Distrito Federal).

##### Notes.

*Humicola capillata* and *H. paraisoensis* clustered in a basal clade sister to a larger group comprising *H. christenseniae*, *H. fuscoatra*, *H. fuscogrisea*, *H. homopilata*, *H. pinnata*, *H. purpurea*, *H. sphaeralis*, and *H. wallefii* (Fig. 5). Morphologically, *H. capillata* and *H. paraisoensis* can be differentiated by ascomatal size (273–339.5 × 131.5–173 μm *vs*. 135.5–290 × 63–159 μm) and ascospores dimensions ((7.5–)8–8.5(–10) × (5.5–)6–6.5(–7) μm *vs*. (6.5–)7.5–8(–9) × 6.5–7(–8) μm). Additionally, *H. capillata* produces olivaceous ascospores and terminal hairs with a hamate tip, whereas *H. paraisoensis* produces brown ascospores and terminal hairs with undulate tips. Moreover, *H. capillata* exhibited a higher growth rate across culture media, including OA (32–34 *vs*.25–28 mm), CMA (28–32 *vs*.25–28 mm), MEA (33–37 *vs*.27–30 mm), and PCA (30–33 *vs*.25–28 mm). *Humicola capillata* produces a sexual morph similar to other species of the genus *Humicola* but differs from its close relatives (Wang et al. 2019b) by producing aseptate, subglobose, or occasionally pyriform aleurioconidia-like conidia. In contrast, while *H. christenseniae* produces subglobose to oblate conidia, sometimes with two cells in chains; *Humicola fuscoatra* produces aseptate, subglobose to oblate conidia, sometimes in chains and in small clusters. *Humicola fuscogrisea* produces subglobose to oblate conidia, solitary or forming chains of 2–3 or many clusters; *Humicola homopilata* produces globose to subglobose conidia, occasionally obovoid or obpyriform, solitary or forming chains of two or small clusters; *Humicola pinnata* produces globose to oblate conidia, sometimes ovoid, without conidiophore differentiation; *Humicola sphaeralis* produces solitary globose to oblate conidia, sometimes ovoid, in hyphae without conidiophore differentiation; *Humicola wallefii* produces solitary conidia, sometimes in pairs in chains or forming small clusters, usually slightly tuberculate, globose to subglobose. Finally, *H. purpurea* differs by producing aseptate, subglobose to ovoid aleurioconidia-like conidia, solitary or in pairs.

#### Humicola paraisoensis

Taxon classificationFungiMonhysteridaChaetomiaceae

D.O. Ramos, P.T.S. Nogueira & O.L. Pereira sp. nov.

54B293CC-52C0-520D-A495-10E3D4F3C813

862448

Fig. 22

##### Etymology.

The name refers to a forest fragment named Mata do Paraíso, from which this fungus was isolated.

**Figure 22.**
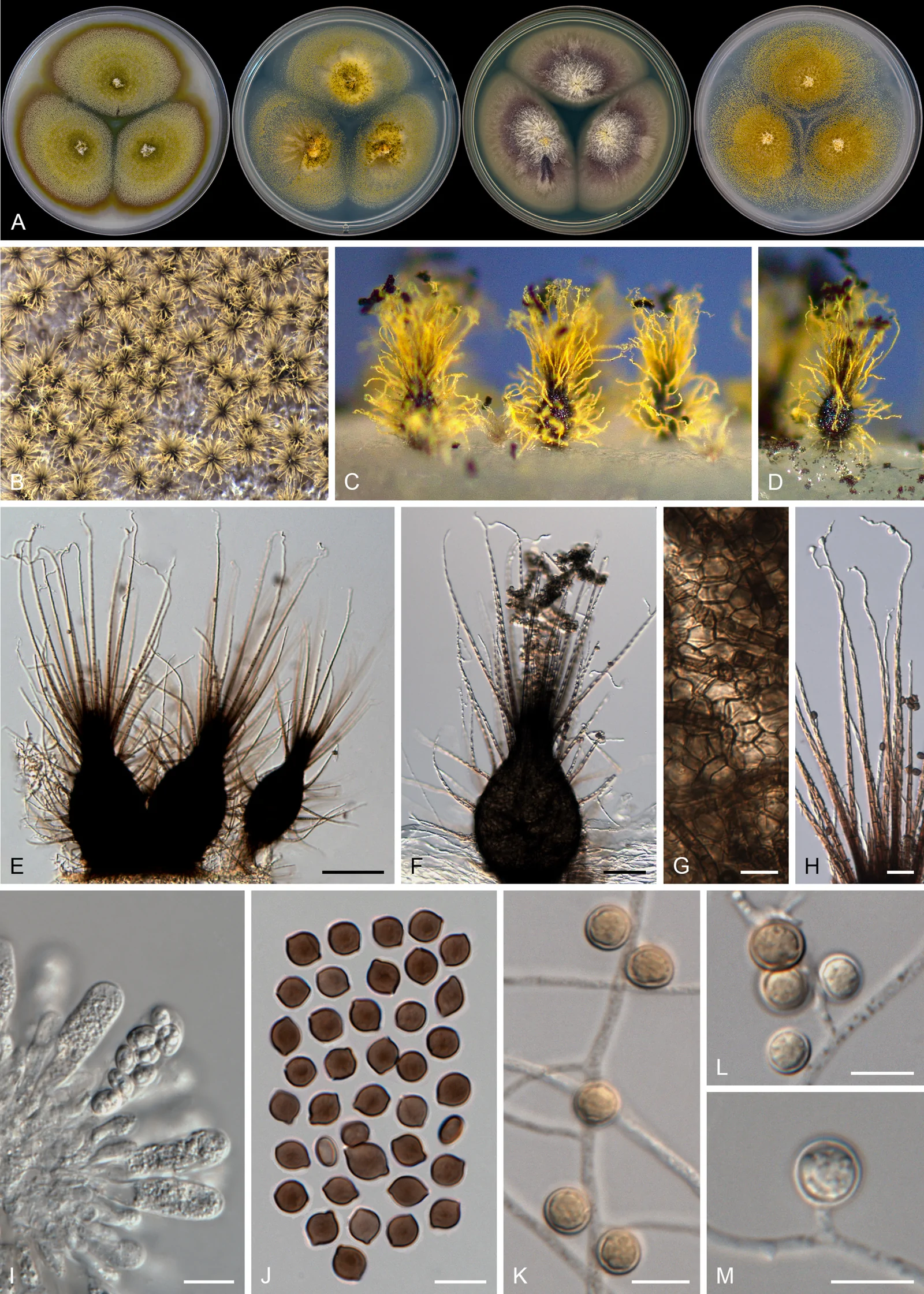
*Humicola paraisoensis*COAD 3743, ex-type. **A**. Colonies on OA, CMA, MEA and PCA from left to right after 4 weeks at 25 °C in the dark; **B**. Top view of mature ascomata on OA; **C, D**. Side view of mature ascomata; **E, F**. Ascomata; **G**. Surface of ascomatal wall; **H**. Terminal ascomatal hairs; **I**. Asci; **J**. Ascospores; **K–M**. Aleurioconidia-like and hyphae. Scale bars: 100 μm (**E**); 50 μm (**F**); 20 μm (**H**); 10 μm (**G, I–M**).

##### Typification.

BRAZIL • Minas Gerais: Viçosa, Mata do Paraíso, from roots of *Catasetum hookeri*, 02 June 2023, D.O. Ramos and P.T.S. Nogueira (***holotype*** VIC 49502 preserved as dried culture, culture ***ex-type***COAD 3743).

##### Description.

***Ascomata*** superficial, ostiolate, luteous to amber because of the ascomatal hairs in reflected light, elongate obpyriform or ampulliform bellow, apically attenuated to an elongate conical or short cylindrical neck, 135.5–290 μm high, 63–159 μm diam. at the widest part. ***Ascomatal wall*** brown, composed of ***textura angularis***. ***Terminal hairs*** arising from the extension of the adjacent ostiolar cells, seta-like with a delicate undulate tip, smooth. ***Lateral hairs*** similar to terminal ones, tapering and fading towards the tips. ***Asci*** clavate to cylindrical, spore-bearing portion 18–27.5 × 6–11 μm, stalks 7–13.5 μm long, 8-spored, biseriate, evanescent. ***Ascospores*** brown, limoniform, umbonate at both ends, bilaterally flattened, (6.5–)7.5–8(–9) × 6.5–7(–8) μm in face view, with one apical germ pore. ***Somatic hyphae*** hyaline. ***Aleurioconidia-like conidia*** produced laterally on hyphae, rarely on branches, solitary, globose, subglobose or occasionally pyriform or ellipsoid, aseptate, subhyaline to pale brown, thick-walled when mature, 7–8.5(–10) × (6–)7–9 μm.

***Culture characteristics***: Colonies on OA attaining 25–28 mm diameter after 7 days at 25 °C, edge entire, aerial mycelium absent, flat, circular; reverse pale vinaceous owing to the presence of pigments. Colonies on CMA attaining 25–28 mm diameter after 7 days at 25 °C, edge entire to fimbriate, white aerial mycelium sparse with little exudate at the center of the colony, flat, circular shape; reverse buff to pale luteous; soluble pigment absent. Colonies on MEA attaining 27–30 mm diameter after 7 days at 25 °C, entire margin, white aerial mycelium thick, texture floccose, flat, circular; reverse dark vinaceous due to production of soluble pigments. Colonies on PCA attaining 25–28 mm diameter after 7 days at 25 °C, edge entire, aerial mycelium absent, flat, circular; reverse buff to saffron; soluble pigments absent.

##### Additional specimens examined.

BRAZIL • Minas Gerais: Viçosa, Mata do Paraíso, from roots of *Catasetum hookeri*, 02 June 2023, D.O. Ramos and P.T.S. Nogueira (living culture COAD 3742).

##### Distribution.

Brazil (Seasonal Semideciduous Forest within the Atlantic Forest domain in Minas Gerais state).

##### Notes.

*Humicola paraisoensis* is phylogenetically related to *H. capillata* (Fig. 5). These two species have different hosts and geographical distributions, *H. paraisoensis* was isolated from the orchid *Catasetum hookeri* in a forest fragment of the Atlantic Forest biome in Minas Gerais state, whereas *H. capillata* was isolated from *Eulophia maculata* in the Cerrado biome in Distrito Federal. *Humicola paraisoensis* produces sexual structures similar to other members of the genus but differs from its close relatives (Wang et al. 2019b) by producing aseptate, solitary, globose, subglobose or occasionally pyriform or ellipsoid aleurioconidia-like conidia. In contrast, *H. christenseniae* produces subglobose to oblate conidia, sometimes occurring in 2-celled chains; *Humicola fuscoatra* produces 1-celled, subglobose to oblate conidia, sometimes in chains and in a few clusters; *Humicola fuscogrisea* produces subglobose to oblate conidia, solitary or forming chains of 2-3 or many clusters; and *Humicola homopilata* produces globose to subglobose conidia, occasionally obovoid or obpyriform, solitary or forming chains of two or small clusters. *Humicola pinnata* produces globose to oblate conidia, sometimes ovoid, without conidiophore differentiation. *Humicola sphaeralis* produces solitary globose to oblate conidia, sometimes ovoid, in hyphae without conidiophore differentiation. *Humicola wallefii* produces solitary conidia, sometimes in pairs in chains or forming small clusters, usually slightly tuberculate, globose to subglobose (Wang et al. 2019b). Finally, *H. purpurea* differs by producing 1-celled, subglobose to ovoid aleurioconidia-like conidia, solitary or in pairs.

#### Humicola purpurea

Taxon classificationFungiMonhysteridaChaetomiaceae

D.O. Ramos, P.T.S. Nogueira & O.L. Pereira sp. nov.

E9887A18-5336-5E4D-B792-4D651CFDF5AB

862447

Fig. 23

##### Etymology.

Refers to the purple pigment produced by this fungus when cultivated on OA and MEA.

**Figure 23.**
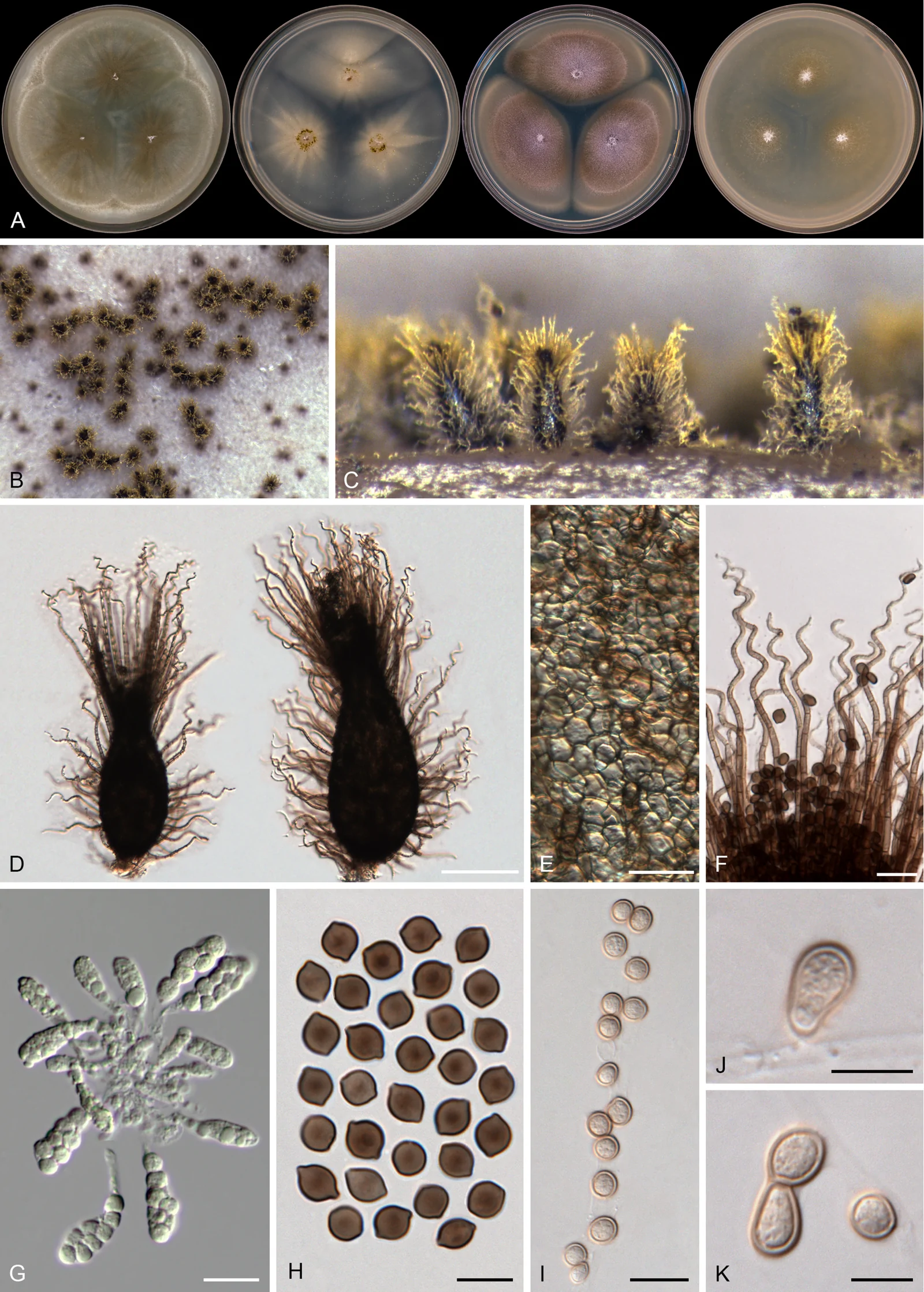
*Humicola purpurea*COAD 3745, ex-type. **A**. Colonies on OA, CMA, MEA and PCA from left to right after 4 weeks at 25 °C in the dark; **B**. Top view of mature ascomata on OA; **C**. Side view of mature ascomata; **D**. Ascomata; **E**. Surface of ascomatal wall; **F**. Terminal ascomatal hairs; **G**. Asci; **H**. Ascospores; **I–K**. Aleurioconidia-like and hyphae. Scale bars: 50 μm (**D**); 20 μm (**E, F, I**); 10 μm (**G, H, J, K**).

##### Typification.

BRAZIL • Minas Gerais: Viçosa, Mata do Paraíso, from roots of *Eulophia maculata*, 02 July 2023, D.O. Ramos (***holotype*** VIC 49503 preserved as dried culture, culture ***ex-type***COAD 3745).

##### Description.

***Ascomata*** superficial, ostiolate, amber in reflected light owing to ascomatal hairs, ovoid to obpyriform, apically attenuated to an elongate conical or short cylindrical neck, 208–280 μm high, 90.5–136 μm diam. at the widest part. ***Ascomatal wall*** brown, composed of ***textura angularis***. ***Terminal hairs*** arising from the extension of the adjacent ostiolar cells, straight, smooth, flexuous to undulate towards the apex. ***Lateral hairs*** similar to terminal ones, tapering and fading towards the tips. ***Asci*** clavate to cylindrical, spore-bearing portion 21.5–29 × 7.5–12 μm, stalks 7.5–14 μm long, 8-spored, biseriate, evanescent. ***Ascospores*** brown, limoniform, umbonate at both ends, bilaterally flattened, (7–)8–8.5(–9.5) × (5.5–)6.5–7(–7.5) μm in face view, with one apical germ pore. ***Somatic hyphae*** hyaline. ***Aleurioconidia-like conidia*** produced laterally on hyphae, solitary or in pairs, subglobose to ovoid, aseptate, pale brown and thick-walled when mature, (8–)8.5–9.5(–11) × (7–)7.5–9(–9.5) μm.

***Culture characteristics***: Colonies on OA attaining 28–30 mm diameter after 7 days at 25 °C, edge entire, aerial mycelium absent, flat, circular; reverse dark vinaceous owing to soluble pigment. Colonies on CMA attaining 30–33 mm diameter after 7 days at 25 °C, entire margin, white aerial mycelium sparse, flat, circular; reverse buff; soluble pigment absent. Colonies on MEA attaining 30–34 mm diameter after 7 days at 25 °C, edge entire, white aerial mycelium sparse, flat, circular, reverse dark vinaceous at the center and pale vinaceous at the margins of colonies due to the presence of pigments. Colonies on PCA attaining 28–32 mm diameter after 7 days at 25 °C, edge entire, white aerial mycelium sparse at the center of the colony and absent at the margins, flat, circular; reverse buff; soluble pigments absent.

##### Additional specimens examined.

BRAZIL • Minas Gerais: Viçosa, Mata do Paraíso, from roots of *Eulophia maculata*, 02 July 2023, D.O. Ramos (living culture COAD 3746).

##### Distribution.

Brazil (Seasonal Semideciduous Forest within the Atlantic Forest domain in Minas Gerais state).

##### Notes.

*Humicola purpurea* clustered into a well-supported clade sister to *H. fuscoatra* (Fig. 5). Morphologically, *H. purpurea* produces a sexual morph in culture, which is not observed for *H. fuscoatra* (Wang et al. 2019b). Additionally, *H. purpurea* showed higher growth rates than *H. fuscoatra* on CMA (30–33 *vs*. 24–30 mm), MEA (30–34 *vs*. 18–24 mm), and PCA (28–32 *vs*. 17–23 mm). *Humicola purpurea* differs from other closely related species (Wang et al. 2019b) by producing aleurioconidia-like conidia that are solitary or in clusters of two, subglobose to ovoid, aseptate, while *H. christenseniae* produces subglobose to oblate conidia, sometimes with two cells in chains. *Humicola fuscogrisea* produces subglobose to oblate conidia, solitary or in chains of 2–3 or in larger clusters. *Humicola homopilata* produces globose to subglobose conidia, occasionally obovoid or obpyriform, solitary or in chains of two or small clusters. *Humicola pinnata* produces globose to oblate conidia, sometimes ovoid, without conidiophore differentiation. *Humicola sphaeralis* produces globose to oblate conidia, sometimes ovoid, solitary in hyphae without conidiophore differentiation. *Humicola wallefii* produces solitary conidia, sometimes in pairs, in chains or forming small clusters, usually slightly tuberculate, globose to subglobose.

#### Pseudohumicola

Taxon classificationFungiMonhysteridaChaetomiaceae

X. Wei Wang, P.J. Han, F.Y. Bai Houbraken, 2022

F226EBB6-107A-539E-97CC-73ACAC8F8169

##### Type species.

*Pseudohumicola subspiralis* (Chivers) X.Wei Wang, P.J. Han, F.Y. Bai & Houbraken, 2022.

#### Pseudohumicola crispata

Taxon classificationFungiMonhysteridaChaetomiaceae

D.O. Ramos & O.L. Pereira sp. nov.

23353E3E-8D61-58F4-B95B-69EE1DBA22DF

862637

Fig. 24

##### Etymology.

From the Latin *crispatus*, due to the presence of undulate to coiled ascomatal hairs.

**Figure 24.**
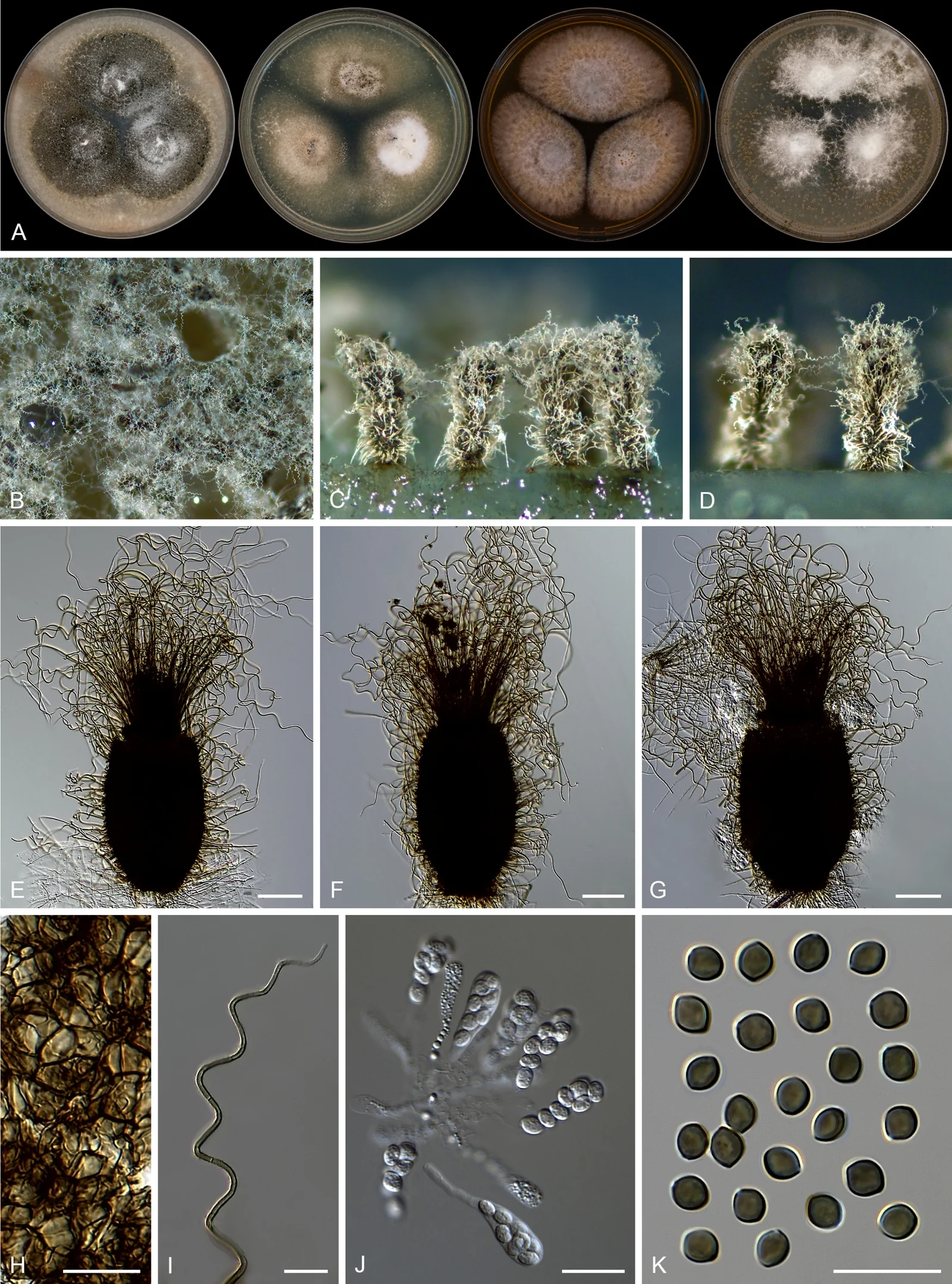
*Pseudohumicola crispata*COAD 4166, ex-type. **A**. Colonies on OA, CMA, MEA and PCA from left to right after 4 weeks at 25 °C in the dark; **B**. Top view of mature ascomata on OA; **C, D**. Side view of mature ascomata; **E–G**. Ascomata; **H**. Surface of ascomatal wall; **I**. Terminal ascomatal hair; **J**. Asci; **K**. Ascospores. Scale bars: 100 μm (**E–G**); 10 μm (**H–K**).

##### Typification.

BRAZIL • Minas Gerais: Santana do Riacho municipality, from roots of *Cattleya* sp. (*Parviflorae*), 13 March 2024, D. O. Ramos (***holotype*** VIC 49703 preserved as dried culture, culture ***ex-type***COAD 4166).

##### Description.

***Ascomata*** superficial, ostiolate, olivaceous buff to greenish olivaceous because of the ascomatal hairs, ovoid to ellipsoid, 158–283.5 μm high, 90.5–163 μm diam. at the widest part. ***Ascomatal wall*** brown, composed of angular to irregular cells. ***Terminal hairs*** straight to flexuous near the base, undulate to coiled and attenuated towards the apex, septate. ***Lateral hairs*** shorter, otherwise similar to the terminal ones. ***Asci*** clavate, spore-bearing portion 12.5–18 × 4.5–8 μm and stalks 6.5–14(–17) μm long, 8 biseriate ascospores, evanescent. ***Ascospores*** gray-olivaceous, limoniform, umbonate at both ends, bilaterally flattened, 3.5–4(–4.5) × 3–3.5(–4) μm in face view, with one apical germ pore. ***Asexual morph*** unknown.

***Culture characteristics***: Colonies on OA attaining 38–43 mm diameter after 7 days at 25 °C, edge entire, white aerial mycelium moderate, flat, circular; reverse vinaceous to pale vinaceous at the center of colonies due to the production of soluble pigment and buff at the margins. Colonies on CMA attaining 38–40 mm diameter after 7 days at 25 °C, undulate margin, white aerial mycelium moderate, flat; reverse pale luteous at the center due to the production of soluble pigment and buff at the margins of colonies. Colonies on MEA attaining 18–20 mm diameter after 7 days at 25 °C, undulate to entire margin, white aerial mycelium moderate, raised, circular; reverse orange to luteous due to the production of soluble pigment. Colonies on PCA attaining 36–40 mm diameter after 7 days at 25 °C, entire edge, with profuse white aerial mycelium, umbonate, circular; reverse buff to pale luteous; soluble pigments absent.

##### Additional specimens examined.

BRAZIL • Minas Gerais: Santana do Riacho municipality, from roots of *Cattleya* sp. (*Parviflorae*), 13 March 2024, D. O. Ramos (living culture COAD 4167).

##### Distribution.

Brazil (Rupestrian Grasslands within the Cerrado domain in Minas Gerais state).

##### Notes.

*Pseudohumicola crispata* is phylogenetically related to *Ps. subspiralis* and morphologically differs from it by the production of smaller ascomata (158–283.5 × 90.5–163 μm *vs*. 240–320 × 185–260 μm), asci (12.5–18 × 4.5–8 μm *vs*. 18–25 × 6–9 μm) and ascospores (3.5–4(–4.5) × 3–3.5(–4) μm *vs*. 5–6.5(–7) × 4.5–5.5(–6). In addition, the acremonium-like synanamorph was not observed in *Ps. crispata* as it was observed in the related species *Ps. subspiralis* by Wang et al. (2019b). Cultures of *Ps. crispata* showed similar growth rates to *Ps. subspiralis* on OA, CMA, and PCA, but were smaller on MEA (18–20 mm *vs*. 30–36 mm diam). The other related species, *Ps. atrobrunnea*, is known for producing only the aleurioconidia-like synanamorph. This is the first *Pseudohumicola* species described as an endophyte worldwide.

#### Pseudohumicola cinnamomeobrunnea

Taxon classificationFungiMonhysteridaChaetomiaceae

Sastoque, Cano & Stchigel, 2025

84DD70C4-3819-5B08-98F3-DF55E2D1BED0

Fig. 25

##### Typification.

SPAIN • Canary Islands: La Palma, Fuencaliente (Los Canarios), isolated from soil of Teneguía volcano, 15 July 2008, M. Calduch and A.M. Stchigel, isolated by A.P. Sastoque (***holotype*** CBS H-25248, culture ***ex-type*** CBS 150900).

**Figure 25.**
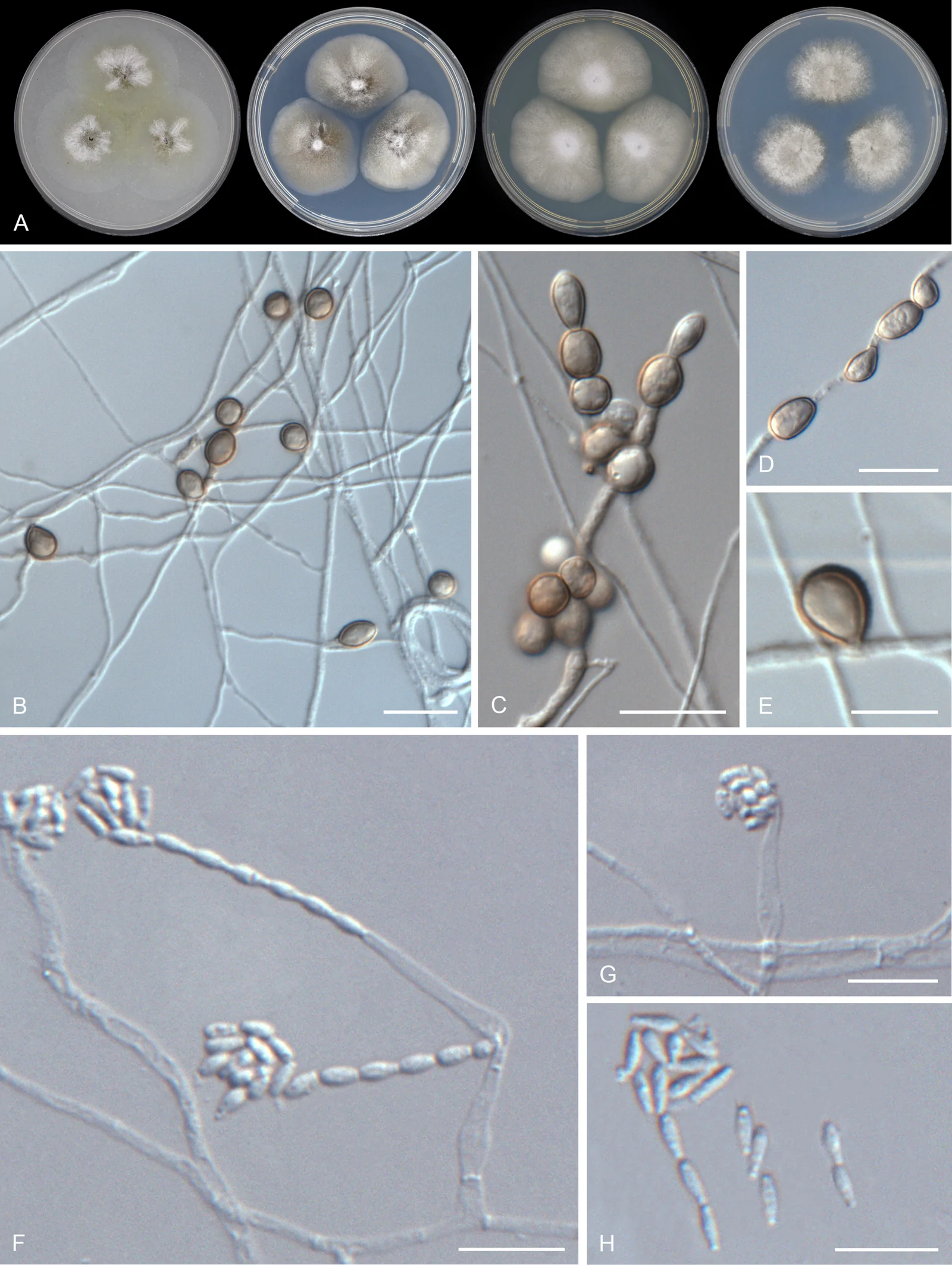
*Pseudohumicola cinnamomeobrunnea*COAD 3747. **A**. Colonies on OA, CMA, MEA, and PCA from left to right after 7 days at 25 °C in the dark; **B–E**. Aleurioconidia-like conidia and hyphae; **F, G**. Acremonium-like condiophores and conidia; **H**. Hyaline conidia. Scale bars: 50 μm (**E, F, G**); 10 μm (**H, I, J, K**).

##### Emended description.

***Somatic hyphae*** hyaline. ***Aleurioconidia-like conidia*** produced laterally on hyphae, but more commonly continuous with the hyphae, solitary or in clusters of three or more, subglobose, occasionally cylindrical or pyriform, 1-celled, with a pale brown to brown, thick wall when mature, (7–)9–10(–15) × (7–)8–8.5(–9.5) μm. ***Acremonium-like conidiophores*** arising laterally from hyphae, branched, sometimes unbranched, aseptate, or sometimes septate, 23.5–42 μm long. ***Conidiogenous cells*** phialidic, 5–21 μm long, 1.5–3.5 μm wide at the base. ***Conidia*** hyaline, borne in basipetal chains or held in false heads, aseptate, clavate, occasionally cylindrical, (3–) 3.5–4.5 (–5) × (1–) 1.5–2 μm. ***Sexual morph*** unknown.

***Culture characteristics***: Colonies on OA attaining 42–46 mm diameter after 7 days at 25 °C, edge entire, aerial mycelium sparse, raised, circular, moderate production of conidia; reverse buff with little production of pale luteous pigment. Colonies on CMA attaining 38–43 mm diameter after 7 days at 25 °C, entire margin, white aerial mycelium sparse, flat, intense production of conidia at the center of colonies; reverse olivaceous at the center and buff at the margins of colonies; soluble pigment absent. Colonies on MEA attaining 45–48 mm diameter after 7 days at 25 °C, edge slightly undulate, white aerial mycelium abundant, raised, circular, conidia production was not observed; reverse pale luteous; soluble pigments absent. Colonies on PCA attaining 34–37 mm diameter after 7 days at 25 °C, edge erose or dentate, white aerial mycelium moderate at the center and sparse at the edges, concave with raised margins, circular, intense production of conidia; reverse olivaceous buff; soluble pigments absent.

##### Specimen examined.

BRAZIL • Minas Gerais, Viçosa, from coconut shell used as substrate for *Cattleya aclandiae*, 25 June 2022, P.T.S Nogueira (herbarium voucher VIC 49504 preserved as dried culture, living culture COAD 3747).

##### Distribution.

Brazil and Spain (Canary Islands).

##### Notes.

Phylogenetic analysis revealed that the isolate COAD 3747 clustered with *Ps. cinnamomeobrunnea* CBS 150900. *Pseudohumicola cinnamomeobrunnea* was described by Sastoque et al. (2025) as producing only aleurioconidia-like synanamorph. However, the isolate COAD 3747 produces an acremonium-like synanamorph, and here we provide its description, which was not observed in the related species, *Ps. semispiralis* and *Ps. glauca*. In addition, colonies of the isolate COAD 3747 showed similar growth rates on MEA and CMA but showed higher growth rates on OA (42–46 mm *vs*. 37–40 mm) and reduced growth on PCA (34–37 mm *vs*. 46–17 mm) when compared to the type CBS 150900. This is the first report of *Ps. cinnamomeobrunnea* outside the Canary Islands, where this species was originally described.

#### Pseudohumicola pulvericola

Taxon classificationFungiMonhysteridaChaetomiaceae

(X. Wei Wang, Houbraken & Seifert) X. Wei Wang, P.J. Han, F.Y. Bai & Houbraken, 2022

1183ACDB-E704-5404-84B1-170FA6E84DB8

Fig. 26

##### Synonym.

*Humicola pulvericola* X.Wei Wang, Houbraken & Seifert, Stud. Mycol. 93: 96 (2019).

**Figure 26.**
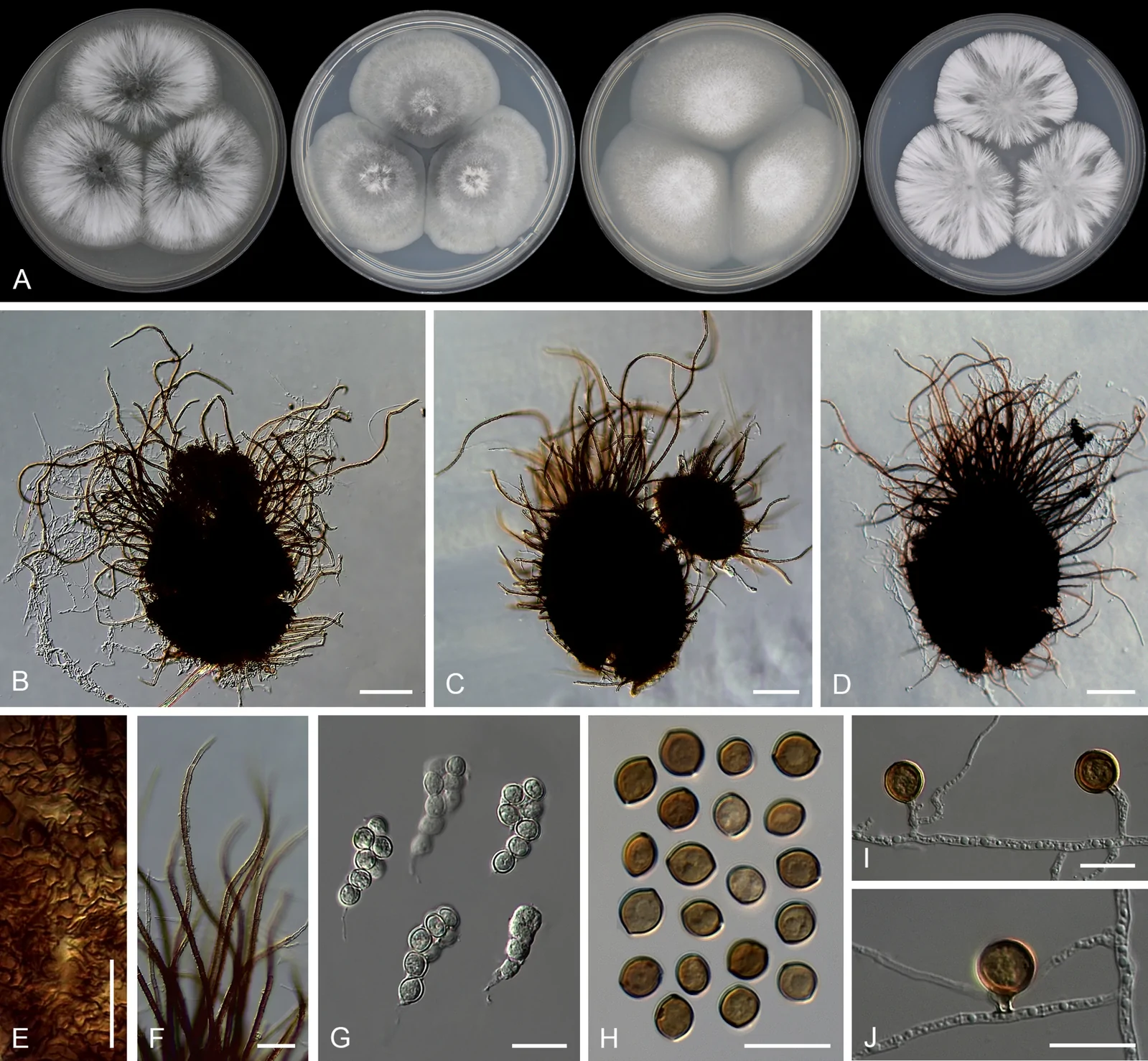
*Pseudohumicola pulvericola*COAD 3748. **A**. Colonies on OA, CMA, MEA and PCA from left to right after 7 days at 25 °C in the dark; **B–D**. Ascomata; **E**. Surface of ascomatal wall; **F**. Terminal ascomatal hairs; **G**. Asci; **H**. Ascospores; **I, J**. Aleurioconidia-like conidia and hyphae. Scale bars: 50 μm (**B–D**); 10 μm (**E, G–J**); 20 μm (**F**).

##### Typification.

MEXICO • From house dust, K. Mwange and E. Whitfield (***holotype*** CBS H-23486, culture ***ex-type*** CBS 144165).

##### Emended description.

***Ascomata*** superficial, ostiolate, ellipsoid to ovoid, 260–408 μm high, 210–308 μm diam., growing under the aerial mycelium. ***Ascomatal wall*** dark brown, composed of ***textura angularis***. ***Terminal hairs*** flexuous, septate, verrucose near the base. ***Lateral hairs*** straight or flexuous. ***Asci*** clavate, with spore-bearing portion 20–25 × 6–8.5 μm, with 8 biseriate ascospores, evanescent. ***Ascospores*** pale brown, limoniform, bilaterally flattened, (9–)10–10.5(–11.5) × (6.5–)8–8.5(–9.5) μm in face view, with one apical germ pore. ***Asexual morph*** described by Wang et al. (2019b).

##### Specimens examined.

BRAZIL • Minas Gerais: Viçosa, from the substrate of *Cattleya aclandiae*, 25 June 2022, P.T.S. Nogueira (living cultures COAD 3748 and COAD 3749).

##### Distribution.

Brazil, Mexico, and South Africa.

##### Notes.

*Pseudohumicola pulvericola* was known only for its asexual morph. In the present study, the sexual morph was observed and described. To induce the production of reproductive structures, isolate COAD 3748 was cultivated on OA using a sterile paper filter, indicating that this is a homothallic species; however, the conditions required to produce the sexual morph are not entirely clear. Ascomata production began after three weeks under the paper filter. This is the first report of this species in Brazil.

## Discussion

### Taxonomy of *Chaetomiaceae*

The taxonomy of the *Chaetomiaceae* family has been thoroughly explored in recent years with a total of 58 genera and approximately 320 species described (Wang et al. 2022b; Hyde et al. 2024; Sastoque et al. 2025, 2026; dos Santos Santana et al. 2026). This rapid increase in taxonomic breadth has been largely driven by the exploration of unconventional hosts and substrates, including volcanic soils, cave environments, and various plant groups. Through a polyphasic taxonomic approach, 22 species were identified in the analyses, almost half of which were new to science. This high proportion of novel lineages underscores the poorly documented fungal diversity associated with wild orchid roots in the Atlantic Forest and Cerrado biomes.

*Arcopilus* was the most abundant genus in the *Chaetomiaceae* found in this study, comprising 21 isolates and eight species. The genus *Arcopilus* has received little attention, with most of its species described in older works and later transferred to the genus in taxonomic revisions (Wang et al. 2016a, 2022b). Our analysis expanded the knowledge of the genus, which now encompasses 16 species, four of which were described in the present study. The new species, *A. endophyticus*, formed a unique outer clade within the genus, near *A. singularis* and three other species that produce sexual morphs. *Arcopilus endophyticus* produces sterile colonies with aggregates of coiled and melanized hyphae, which have not been observed in the genus. In the genus *Chaetomium*, species such as *C. capillare*, *C. cervicicola*, and *C. cucumericola* were considered sterile due to the absence of reproductive structures (Wang et al. 2016b). Another species with atypical characteristics for the genus is *A. singularis*, which produces an acremonium-like synanamorph and lacks ascomata, both of which are unprecedented in the genus. Moreover, the species *A. albae* is here synonymized under *A. cupreus* based on sequence and morphological data. Similarly, Wang et al. (2022b) synonymized *A. tangerinicapillus* with *A. cupreus* and *A. globulus* with *A. aureus* in the most recent revision of the family.

The genus *Chaetomium* was introduced by Kunze and Schmidt (1817) and has since undergone constant changes. Over time, many studies on the genus *Chaetomium* have been conducted, and as a result, many fungi known as chaetomium-like have been revised in terms of their morphology and phylogeny. Consequently, numerous species have been synonymized, proposed within the genus, or reclassified into other genera within *Chaetomiaceae* (Wang et al. 2016a, 2016b; Manawasinghe et al. 2021; Sastoque et al. 2026). Owing to these changes and updates, *Chaetomium* is arguably the most speciose genus in *Chaetomiaceae*, with 46 species. In this study, 12 isolates were placed in five known species of *Chaetomium*: *C. coarctatum*, *C. cochliodes*, *C. globosum*, *C. pseudocochliodes*, and *C. tenue*. *Chaetomium albiziae*, previously considered a distinct species, was synonymized with *C. cucumericola* in this study. Some inconsistencies still appear in the genus when phylogenies are made, as in the case of *C. microthecium*, which in some phylogenies from regions such as ITS and *rpb2* is grouped within *C. globosum* species complex. Further studies are needed to reassess the phylogenetic placement of *C. microthecium* within the genus.

The genus *Collariella* encompasses 13 species, characterized by superficial ascomata with truncated apices and a darkened collar around the ostiolar pore (Wang et al. 2016a). The genus was initially divided into two subclades, but subclade two was subsequently elevated to genus status as *Achaetomiella* (Wang et al. 2022b). Multi-locus phylogenetic analyses based on the *tub2* and *rpb2* loci, as well as the concatenated dataset, revealed that *Col. fluminensis* forms a well-supported clade sister to *Col. capillicompacta*, *Col. carteri*, *Col. pachypodioides*, and *Col. hongheensis*. The consistent topology observed across these individual gene trees, coupled with significant molecular divergence, provides robust evidence for its recognition as a distinct species. Furthermore, the novel species differs from its closest relatives by producing larger ascomata with fusiform to ovoid shape and slightly larger ascospores.

The *Dichotomopilus* isolates obtained in this study were grouped within the *D. funicola* species complex. This species complex, defined by Wang et al. (2016a), comprises *D. finlandicus*, *D. funicola*, *D. pseudofunicola*, *D. subfunicola*, and *D. variostiolatus*. In this study, we introduce *D. brasiliensis* to this complex. This species is closely related to *D. subfunicola* but was grouped separately from it in the individual phylogenies of *tub2* and *rpb2*. Although ITS sequences showed no differences between these two species, this was expected because these sequences cannot be separated at the species level (Hambleton et al. 2005). *Dichotomopilus brasiliensis* is morphologically similar to other species in the *D. funicola* complex, with ovate to elongate-ovate ascospores, shorter terminal ascomatal hairs, which sometimes form a reticulate network to retain the ascospore mass. The other isolates clustered with the *D. variostiolatus* clade and were near the type strain of this species.

The genus *Humicola* was traditionally defined as an asexual lineage within *Chaetomiaceae*, but recent phylogenetic revisions have clarified its taxonomic position, currently encompassing 23 recognized species (Wang et al. 2019b; Nogueira et al. 2025; Kim et al. 2026; Sastoque et al. 2026). In this study, the identification of *H. capillata*, *H. paraisoensis*, and *H. purpurea* was supported by multi-locus phylogenetic analysis and by morphological characters. Although phylogenetic analysis revealed a close relationship between *H. capillata* and *H. paraisoensis*, grouping them as sister clades, the comparative analysis of four gene loci confirms their status as distinct species. This molecular divergence is further supported by clear diagnostic morphological differences; the two taxa exhibit significant variation in the morphology of the terminal hairs, ascomatal size, ascospore color, and colony morphology. Consequently, the integration of multi-locus phylogeny and micromorphological evidence validates *H. capillata* and *H. paraisoensis* as independent species within the genus *Humicola*.

Wang et al. (2022b) reclassified a few *Humicola* species into the new genus *Pseudohumicola*. Currently, this genus comprises 18 species, three of which have been described in Brazilian caves: *Ps. cecavii*, *Ps. alba*, and *Ps. lutea* (Alves et al. 2022; Condé et al. 2023; Sastoque et al. 2025, 2026; Kim et al. 2026). The taxonomy of *Pseudohumicola* has undergone several additions with description of new species and reclassification of strains previously assigned to *Humicola* (Sastoque et al. 2025, 2026). While most *Pseudohumicola* species are known to produce only an asexual morph with aleurioconidia-like conidia or acremonium-like conidiophores, *Ps. crispata* is the only species of the genus characterized by the production of only a sexual morph. Furthermore, this study provides supplementary morphological data for two previously described species. *Pseudohumicola cinnamomeobrunnea*, which is phylogenetically related to *Ps. glauca* and *Ps. semispiralis*, was originally described based on its aleurioconidia-like morphology; here, we provide the first description of its acremonium-like synanamorph. Similarly, *Ps. pulvericola*, initially described by Wang et al. (2019b) only in its asexual state, is here reported and described with its corresponding sexual morph.

### Endophytic association of *Chaetomiaceae* in Brazil

Brazilian orchids harbor a great diversity of root endophytic fungi, which are still poorly explored by taxonomists. Recently, new fungal species have been described from roots of native orchids, such as *Colletotrichum serranegrense* in *Cattleya jongheana* (Silva et al. 2018), *Trichoderma orchidacearum* in *Anacheilium allemanoides* (Morgan et al. 2022), and *Cladophialophora chlamydospora*, *Cl. fuscoatra*, *Purimyces orchidacearum*, *Pu. endophyticus*, *Penicillium endophyticum*, *Talaromyces cattleyae*, and *Toxicocladosporium atratum* in *Cattleya locatellii* (Crous et al. 2024, 2025, 2026; Condé et al. 2025; Silva et al. 2026). However, fungi in *Chaetomiaceae* have not been studied or reported as endophytes in Brazilian *Orchidaceae* plants to date.

In the present study, *Arcopilus* was found in six native orchids with epiphytic, lithophytic, and terrestrial lifestyles. Other studies have reported new endophytic *Arcopilus* species, such as *A. amazonicus*, *A. eremanthi*, and *A. hongheensis*, the first two of which were discovered in native Brazilian plants (Sousa et al. 2020; Tavares et al. 2022; Yang et al. 2023). The biotechnological potential of endophytic *Arcopilus* isolates has been demonstrated in previous studies, including antifungal, antibacterial, and antioxidant activities (Dwibedi and Saxena 2018, 2020; Tavares et al. 2022) as well as pigment production (Silva et al. 2022). The production of soluble pigments in culture media by *A. abbreviatus*, *A. endophyticus*, and *A. singularis* requires further investigation to verify the putative biotechnological potential of these isolates.

The genus *Chaetomium* is widely distributed in various environments, including air, dung, soil, plants, and marine ecosystems (Wang et al. 2016a, 2016b). Species of this genus are well known for their ability to degrade cellulose-rich substrates and produce secondary metabolites with potential use in agricultural, pharmaceutical, and industrial applications (Wang et al. 2016a; Abdel-Azeem et al. 2021; Dwibedi et al. 2023). *Chaetomium* species identified in this study were found in five orchids with epiphytic and terrestrial lifestyles, suggesting the wide distribution of this genus in Brazilian orchids. Among the *Chaetomium* species identified, *C. globosum* was the only one found as a root endophyte on three different orchids: *Eulophia maculata*, *Polystachya concreta*, and *Zygopetalum mackayi*, which is consistent with previous studies that frequently reported this species as an endophyte in plants from different groups, including orchids (Adit et al. 2022; dos Santos Santana et al. 2026).

To date, the only *Collariella* species reported in Brazil is *Col. bostrychodes*, found in animal dung and in leaf litter from a cave (Melo et al. 2012; Condé et al. 2023). However, the genus *Collariella* was found to be endophytically associated with *Melocactus ernestii*, a cactus from the Brazilian Caatinga biome (Ferreira-Silva et al. 2021). Recently, *Col. hongheensis* was described as a leaf endophyte of *Mangifera indica* in China (Manawasinghe et al. 2024). *Collariella fluminensis* was isolated from the roots of two terrestrial orchids, *Cyclopogon congestus* and *Eulophia maculata*, in two Brazilian states. This is the only new species found in this study across two distinct plant species.

*Dichotomopilus brasiliensis* is introduced here as a root endophyte of the orchid *Zygopetalum mackayi*, as well as *Musa* sp. (banana) and *Theobroma cacao* (cocoa). Root-endophytic species of *Dichotomopilus* have been identified on different plants in Brazil, including the new endophytic species *D. endophyticus* from the roots of *Musa acuminata* (Oliveira et al. 2024; dos Santos Santana et al. 2026). In this study, two endophytic isolates were identified as *D. variostiolatus* in two *Cattleya* species. This species was also identified by dos Santos Santana et al. (2026) as a root endophyte of *Musa acuminata* and might be a common endophyte of plants in Brazil.

Members of the genus *Humicola* are frequently recovered from soil environments; for instance, *H. leptodermospora*, *H. quadranculata*, and *H. cerradensis* were all originally described from various Brazilian soil types (Wang et al. 2019b; Nogueira et al. 2025). In contrast, the novel species identified in this study were isolated as endophytes from orchids inhabiting distinct biomes and forest fragments. Specifically, *H. capillata* was obtained from the Cerrado (dry forest), whereas *H. paraisoensis* and *H. purpurea* were isolated from orchids in wet forest environments. The endophytic nature of *Humicola* has been increasingly recognized and the potential applicability of these fungi has been explored. Hosseyni Moghaddam et al. (2020) reported *H. fuscoatra* from halophytic plants, noting its capacity to produce metabolites with significant antiproliferative, antifungal, and antibacterial properties against diverse agricultural pathogens. Similarly, *H. wallefii* was recorded as an endophyte from the leaves of *Mangifera indica* (Yang et al. 2023). These findings reinforce that the ecological range of *Humicola* extends significantly beyond soil saprophytism, encompassing complex symbiotic associations within diverse plant tissues.

The species *Pseudohumicola crispata*, introduced here as an endophyte of a lithophytic *Cattleya* sp., is the first species of the genus reported in an endophytic association worldwide. *Pseudohumicola cinnamomeobrunnea* was originally described from volcanic soil (Sastoque et al. 2025), and *Ps. pulvericola* was described from house dust (Wang et al. 2019b). Both species are reported here growing near the roots of the same plant, *Cattleya aclandiae*, on the substrate. Unlike *Ps. crispata*, these isolates were not recovered from within the plant tissues, but rather as decomposers on the substrate. This saprophytic habit aligns with previous reports for most species in the genus, which have been documented in diverse environments such as air, soil, dust, paper, and leaf litter (Wang et al. 2019b, 2022b; Condé et al. 2023).

## Conclusion

This study examined 51 specimens of *Chaetomiaceae*, of which 48 were endophytic and three were from coconut substrates used for orchid cultivation. Here, we present ten new taxonomic findings: four in the genus *Arcopilus*, one new species in *Collariella*, one new species in *Dichotomopilus*, three new species in *Humicola*, and one new species in *Pseudohumicola*. Two new synonyms are also proposed: one in *Arcopilus* and one in *Chaetomium*. In total, 32 isolates represented 12 known species in *Chaetomiaceae*, of which six had not yet been recorded in Brazil, and here we contribute to the geographic expansion of these species. This study contributes to knowledge of geographic distribution, hosts, and substrates on which these fungi occur.

Despite the potential for diverse functions attributed to *Chaetomiaceae*, the ecological role of the association between *Chaetomiaceae* species and orchids or other plants remains unclear. Furthermore, Brazil still has a remarkably low number of described fungal species relative to its estimated diversity. This study exemplifies that even with a limited sampling of orchid species and specimens, significant taxonomic novelties can be uncovered, highlighting the vast unexplored fungal diversity in the country.

Furthermore, the taxonomic study of fungi associated with orchids may be the first step in understanding the fungus-host relationship, and future studies aimed at investigating biotechnological applications associated with these isolates are crucial, given that fungi with the potential to act as biological control agents against plant diseases and promote plant growth have already been reported in *Chaetomiaceae*.

## Supplementary Material

XML Treatment for Arcopilus

XML Treatment for Arcopilus abbreviatus

XML Treatment for Arcopilus endophyticus

XML Treatment for Arcopilus kasuyae

XML Treatment for Arcopilus singularis

XML Treatment for Arcopilus amazonicus

XML Treatment for Arcopilus aureus

XML Treatment for Arcopilus cupreus

XML Treatment for Arcopilus macrostiolatus

XML Treatment for Chaetomium

XML Treatment for Chaetomium coarctatum

XML Treatment for Chaetomium cochliodes

XML Treatment for Chaetomium ovatoascomatis

XML Treatment for Chaetomium globosum

XML Treatment for Chaetomium pseudocochliodes

XML Treatment for Chaetomium tenue

XML Treatment for Collariella

XML Treatment for Collariella fluminensis

XML Treatment for Dichotomopilus

XML Treatment for Dichotomopilus brasiliensis

XML Treatment for Dichotomopilus variostiolatus

XML Treatment for Humicola

XML Treatment for Humicola capillata

XML Treatment for Humicola paraisoensis

XML Treatment for Humicola purpurea

XML Treatment for Pseudohumicola

XML Treatment for Pseudohumicola crispata

XML Treatment for Pseudohumicola cinnamomeobrunnea

XML Treatment for Pseudohumicola pulvericola

## Additional information

### Artificial Intelligence (AI) use

The authors accept full responsibility for the content of the manuscript, including the disclosure of any use of AI.

Regarding the use of AI in the preparation of this manuscript, the authors declare the following: Paperpal.

**Used for**: Language, style and writing.

The authors used Paperpal (an AI-based writing assistant) to improve the language and grammar of this manuscript. After using this tool, the authors reviewed and edited the content as needed and take full responsibility for the final version of the manuscript.

### Adherence to national and international regulations

All the fungal strains used in this study have been legally obtained, respecting the Convention on Biological Diversity (Rio Convention).

### Author ORCIDs

D.O. Ramos https://orcid.org/0000-0003-1868-0350

P.T.S. Nogueira https://orcid.org/0009-0004-4139-8630

T.O. Condé https://orcid.org/0000-0001-8614-6649

J. dos Santos Santana https://orcid.org/0009-0007-6270-4512

O.L. Pereira https://orcid.org/0000-0002-0274-4623

### Data availability

Fungal isolates are deposited in public culture collections in Brazil, DNA sequences are available in GenBank, and alignments were deposited in Figshare.

## Supplementary materials

Supplementary material 1 GenBank accession numbers of the *Chaetomiaceae* used in the phylogenetic analyses Data type docx https://binary.pensoft.net/file/1778101This dataset is made available under the Open Database License (http://opendatacommons.org/licenses/odbl/1.0/). The Open Database License (ODbL) is a license agreement intended to allow users to freely share, modify, and use this Dataset while maintaining this same freedom for others, provided that the original source and author(s) are credited. Danilo Oliveira Ramos, Pedro Thiago Santos Nogueira, Thiago Oliveira Condé, Jaciara dos Santos Santana, Olinto Liparini Pereira

Supplementary material 2 Supplementary images Data type zip Explanation note **figure S1**. Bayesian phylogenetic tree of *Arcopilus*, *Collariella* and related genera based on dataset of ITS sequences. The isolates obtained in this study are highlighted in bold. Ex-type isolates are marked with ‘‘^T^’’. Only values above 80%, for bootstrap (bs), and above 0.80 for posterior probabilities (pp), are shown near nodes. The tree is rooted with *Stellatospora terricola* CBS 811.95. **figure S2**. Bayesian phylogenetic tree of *Arcopilus*, *Collariella* and related genera based on dataset of LSU sequences. The isolates obtained in this study are highlighted in bold. Ex-type isolates are marked with ‘‘^T^’’. Only values above 80%, for bootstrap (bs), and above 0.80 for posterior probabilities (pp), are shown near nodes. The tree is rooted with *Stellatospora terricola* CBS 811.95. **figure S3**. Bayesian phylogenetic tree of *Arcopilus*, *Collariella* and related genera based on dataset of *rpb2* sequences. The isolates obtained in this study are highlighted in bold. Ex-type isolates are marked with ‘‘^T^’’. Only values above 80%, for bootstrap (bs), and above 0.80 for posterior probabilities (pp), are shown near nodes. The tree is rooted with *Stellatospora terricola* CBS 811.95. **figure S4**. Bayesian phylogenetic tree of *Arcopilus*, *Collariella* and related genera based on dataset of *tub2* sequences. The isolates obtained in this study are highlighted in bold. Ex-type isolates are marked with ‘‘^T^’’. Only values above 80%, for bootstrap (bs), and above 0.80 for posterior probabilities (pp), are shown near nodes. The tree is rooted with *Stellatospora terricola* CBS 811.95. **figure S5**. Bayesian phylogenetic tree of *Chaetomium* based on dataset of ITS sequences. The isolates obtained in this study are highlighted in bold. Ex-type isolates are marked with ‘‘^T^’’. Only values above 80%, for bootstrap (bs), and above 0.80 for posterior probabilities (pp), are shown near nodes. The tree is rooted with *Xanthiomyces spinosus* CBS 789.71 and CBS 796.83. **figure S6**. Bayesian phylogenetic tree of *Chaetomium* based on dataset of LSU sequences. The isolates obtained in this study are highlighted in bold. Ex-type isolates are marked with ‘‘^T^’’. Only values above 80%, for bootstrap (bs), and above 0.80 for posterior probabilities (pp), are shown near nodes. The tree is rooted with *Xanthiomyces spinosus* CBS 789.71 and CBS 796.83. **figure S7**. Bayesian phylogenetic tree of *Chaetomium* based on dataset of *rpb2* sequences. The isolates obtained in this study are highlighted in bold. Ex-type isolates are marked with ‘‘^T^’’. Only values above 80%, for bootstrap (bs), and above 0.80 for posterior probabilities (pp), are shown near nodes. The tree is rooted with *Xanthiomyces spinosus* CBS 789.71 and CBS 796.83. **figure S8**. Bayesian phylogenetic tree of *Chaetomium* based on dataset of *tub2* sequences. The isolates obtained in this study are highlighted in bold. Ex-type isolates are marked with ‘‘^T^’’. Only values above 80%, for bootstrap (bs), and above 0.80 for posterior probabilities (pp), are shown near nodes. The tree is rooted with *Xanthiomyces spinosus* CBS 789.71 and CBS 796.83. **figure S9**. Bayesian phylogenetic tree of *Dichotomopilus* based on dataset of ITS sequences. The isolates obtained in this study are highlighted in bold. Ex-type isolates are marked with ‘‘^T^’’. Only values above 80%, for bootstrap (bs), and above 0.80 for posterior probabilities (pp), are shown near nodes. The tree is rooted with *Xanthiomyces spinosus* CBS 789.71 and CBS 796.83. **figure S10**. Bayesian phylogenetic tree of *Dichotomopilus* based on dataset of LSU sequences. The isolates obtained in this study are highlighted in bold. Ex-type isolates are marked with ‘‘^T^’’. Only values above 80%, for bootstrap (bs), and above 0.80 for posterior probabilities (pp), are shown near nodes. The tree is rooted with *Xanthiomyces spinosus* CBS 789.71 and CBS 796.83. **figure S11**. Bayesian phylogenetic tree of *Dichotomopilus* based on dataset of *rpb2* sequences. The isolates obtained in this study are highlighted in bold. Ex-type isolates are marked with ‘‘^T^’’. Only values above 80%, for bootstrap (bs), and above 0.80 for posterior probabilities (pp), are shown near nodes. The tree is rooted with *Xanthiomyces spinosus* CBS 789.71 and CBS 796.83. **figure S12**. Bayesian phylogenetic tree of *Dichotomopilus* based on dataset of *tub2* sequences. The isolates obtained in this study are highlighted in bold. Ex-type isolates are marked with ‘‘^T^’’. Only values above 80%, for bootstrap (bs), and above 0.80 for posterior probabilities (pp), are shown near nodes. The tree is rooted with *Xanthiomyces spinosus* CBS 789.71 and CBS 796.83. **figure S13**. Bayesian phylogenetic tree of *Humicola* and *Pseudohumicola* based on dataset of ITS sequences. The isolates obtained in this study are highlighted in bold. Ex-type isolates are marked with ‘‘^T^’’. Only values above 80%, for bootstrap (bs), and above 0.80 for posterior probabilities (pp), are shown near nodes. The tree is rooted with *Allobotryotrichum blastosporum* CGMCC 3.1943. **figure S14**. Bayesian phylogenetic tree of *Humicola* and *Pseudohumicola* based on dataset of LSU sequences. The isolates obtained in this study are highlighted in bold. Ex-type isolates are marked with ‘‘^T^’’. Only values above 80%, for bootstrap (bs), and above 0.80 for posterior probabilities (pp), are shown near nodes. The tree is rooted with *Allobotryotrichum blastosporum* CGMCC 3.1943. **figure S15**. Bayesian phylogenetic tree of *Humicola* and *Pseudohumicola* based on dataset of *rpb2* sequences. The isolates obtained in this study are highlighted in bold. Ex-type isolates are marked with ‘‘^T^’’. Only values above 80%, for bootstrap (bs), and above 0.80 for posterior probabilities (pp), are shown near nodes. The tree is rooted with *Allobotryotrichum blastosporum* CGMCC 3.1943. **figure S16**. Bayesian phylogenetic tree of *Humicola* and *Pseudohumicola* based on dataset of *tub2* sequences. The isolates obtained in this study are highlighted in bold. Ex-type isolates are marked with ‘‘^T^’’. Only values above 80%, for bootstrap (bs), and above 0.80 for posterior probabilities (pp), are shown near nodes. The tree is rooted with *Allobotryotrichum blastosporum* CGMCC 3.1943. https://binary.pensoft.net/file/1778102This dataset is made available under the Open Database License (http://opendatacommons.org/licenses/odbl/1.0/). The Open Database License (ODbL) is a license agreement intended to allow users to freely share, modify, and use this Dataset while maintaining this same freedom for others, provided that the original source and author(s) are credited. Danilo Oliveira Ramos, Pedro Thiago Santos Nogueira, Thiago Oliveira Condé, Jaciara dos Santos Santana, Olinto Liparini Pereira

Supplementary material 3 Concatenated alignment of *Arcopilus* and *Collariella*, *Chaetomium*, *Dichotomopilus*, *Humicola* and *Pseudohumicola* Data type zip https://binary.pensoft.net/file/1778103This dataset is made available under the Open Database License (http://opendatacommons.org/licenses/odbl/1.0/). The Open Database License (ODbL) is a license agreement intended to allow users to freely share, modify, and use this Dataset while maintaining this same freedom for others, provided that the original source and author(s) are credited. Danilo Oliveira Ramos, Pedro Thiago Santos Nogueira, Thiago Oliveira Condé, Jaciara dos Santos Santana, Olinto Liparini Pereira
