# Supplementary material for: Diversity and taxonomy of endophytic Chaetomiaceae (Sordariales) associated with roots of wild Orchidaceae in Brazil
Source: IMA Fungus. 2026 Sep 18;17:e192047. doi: 10.3897/imafungus.17.192047 (PMC13615399; doi:10.3897/imafungus.17.192047)
Supplement: Supplementary file 1 [file imafungus-17-e192047-s001.docx]

**Table S1:** GenBank accession numbers of the *Chaetomiaceae* used in the phylogenetic analyses.

| **Species** | **Culture accession** | **Genbank accession number** | | | | **Reference** |
| --- | --- | --- | --- | --- | --- | --- |
|  |  | **ITS** | **LSU** | ***TUB*** | ***RPB2*** |  |
| *Achaetomiella gracilis* | CBS 146.60^T^ | KX976648 | KX976743 | KX976990 | KX976842 | Wang et al. (2022) |
| *Achaetomiella virescens* | CBS 148.68^T^ | KX976654 | KX976749 | KX976996 | KX976848 | Wang et al. (2022) |
| *Achaetomium globosum* | CBS 332.67^T^ | KX976570 | KX976695 | KX976911 | KX976793 | Wang et al. (2016a) |
| *Achaetomium lentum* | CBS 618.68 | KX976571 | KX976696 | KX976912 | KX976794 | Wang et al. (2022) |
| *Achaetomium macrosporum* | CBS 152.97^T^ | KX976573 | KX976698 | KX976914 | KX976796 | Wang et al. (2016a) |
| *Achaetomium strumarium* | CBS 333.67^T^ | AY681204 | AY681170 | AY681238 | KC503254 | Wang et al. (2016a) |
| *Allobotryotrichum blastosporum* | CGMCC 3.19343^T^ | MN215716 | MN215554 | MN329887 | MN255397 | Raza et al. (2019) |
| *Arcopilus albae* | MFLUCC 24–0064^T^ | PP766582 | PP766589 | PP801102 | – | Xu et al. (2024) |
| *Arcopilus amazonicus* | AM2410^T^ | MH777083 | MH780043 | MH784466 | MH784457 | Souza et al. (2020) |
| *Arcopilus amazonicus* | AM2411 | MH777084 | MH780044 | MH784467 | MH784458 | Souza et al. (2020) |
| *Arcopilus aureus* | CBS 153.52 | KX976582 | KX976707 | KX976924 | KX976806 | Wang et al. (2016a) |
| *Arcopilus aureus* | CBS 538.73 | KX976583 | KX976708 | KX976925 | KX976807 | Wang et al. (2016a) |
| *Arcopilus cupreus* | CBS 560.80 | KX976584 | KX976709 | KX976926 | KX976808 | Wang et al. (2016a) |
| *Arcopilus cupreus* | LC11938 | MN215723 | MN215561 | MN329894 | MN255404 | Raza et al. (2019) |
| *Arcopilus eremanthi* | CML 3766^T^ | MN539886 | MN539910 | – | MN551186 | Tavares et al. (2021) |
| *Arcopilus flavigenus* | CBS 337.67^T^ | KX976587 | KX976712 | KX976811 | KX976929 | Wang et al. (2016a) |
| *Arcopilus fusiformis* | CBS 484.85 | KX976585 | KX976710 | KX976809 | KX976927 | Wang et al. (2016a) |
| *Arcopilus fusiformis* | CBS 485.85 | KX976586 | KX976711 | KX976810 | KX976928 | Wang et al. (2016a) |
| *Arcopilus hongheensis* | KUNCC22-10767^T^ | OR117280 | OR117285 | OR119776 | OR119771 | Yang et al. (2023) |
| *Arcopilus hongheensis* | KUMCC 23-13346 | OR117279 | OR117284 | OR119775 | OR119770 | Yang et al. (2023) |
| *Arcopilus macrostiolatus* | CBS 102435^T^ | MZ334722 | MZ351418 | MZ342965 | MZ343006 | Wang et al. (2022) |
| *Arcopilus megasporum* | CBS 127650 | – | MH876091 | MZ343010 | MZ342971 | Wang et al. (2022) |
| *Arcopilus navicularis* | CCF 3252^T^ | MW798185 | MW798181 | MW816124 | MW816124 | Crous et al. (2021) |
| *Arcopilus purpurascens* | CBS 287.83^T^ | – | – | MZ342982 | MZ343021 | Wang et al. (2022) |
| *Arcopilus turgidopilosus* | CBS 169.52^T^ | KX976588 | KX976713 | KX976930 | KX976812 | Wang et al. (2016a) |
| *Chaetomium afropilosum* | CBS 145.38^T^ | KT214574 | KT214605 | KT214751 | KT214675 | Wang et al. (2016b) |
| *Chaetomium albiziae* | IRAN 4137C^T^ | OL415259 | – | OK032443 | OK032445 | Mehrabi-Koushki et al. (2023) |
| *Chaetomium albiziae* | SCUA-Saf-B10-2 | OL415260 | – | OK032444 | OK032446 | Mehrabi-Koushki et al. (2023) |
| *Chaetomium angustispirale* | CBS 137.58^T^ | JN209862 | JN209862 | JN256141 | KF001824 | Wang et al. (2016b) |
| *Chaetomium ascotrichoides* | CBS 113.83^T^ | KC109752 | KC109752 | KC109770 | KF001832 | Wang et al. (2016b) |
| *Chaetomium ascotrichoides* | CBS 110.83 | KC109753 | KC109753 | KC109771 | KF001833 | Wang et al. (2016b) |
| *Chaetomium camelliae* | JZB3340001^T^ | MT535751 | MT535749 | MT535533 | MT535537 | Manawasinghe et al. (2021) |
| *Chaetomium camelliae* | JZB3340002 | MT535752 | MT535750 | MT535534 | MT535538 | Manawasinghe et al. (2021) |
| *Chaetomium capillare* | CBS 128489^T^ | KT214583 | KT214614 | KT214760 | KT214686 | Wang et al. (2016b) |
| *Chaetomium cervicicola* | CBS 128492^T^ | KT214558 | KT214592 | KT214735 | KT214662 | Wang et al. (2016b) |
| *Chaetomium chirrhatum* | CGMCC 3.17540^T^ | KP336792 | KP336841 | KP336890 | KT149508 | Zhang et al. (2017a) |
| *Chaetomium chirrhatum* | CGMCC 3.17541 | KP336793 | KP336842 | KP336891 | KT149509 | Zhang et al. (2017a) |
| *Chaetomium citrinum* | CBS 693.82^T^ | KT214587 | KT214617 | KT214764 | KT214691 | Wang et al. (2016b) |
| *Chaetomium coarctatum* | CBS 162.62^T^ | JN209863 | JN209863 | JN256142 | KF001802 | Wang et al. (2016b) |
| *Chaetomium coarctatum* | CGMCC 3.14299 | JN209924 | JN209924 | JN256194 | KF001804 | Wang et al. (2016b) |
| *Chaetomium cochliodes* | CBS 155.52^T^ | KC109754 | KC109754 | KC109772 | KF001811 | Wang et al. (2016b) |
| *Chaetomium cochliodes* | CGMCC 3.9440 | JN209866 | JN209866 | JN256145 | KF001814 | Wang et al. (2016b) |
| *Chaetomium contagiosum* | CBS 128494^T^ | KT214555 | KT214589 | KT214732 | KT214659 | Wang et al. (2016b) |
| *Chaetomium cruentum* | CBS 371.66 | JN209871 | JN209871 | JN256148 | KF001795 | Wang et al. (2016b) |
| *Chaetomium cucumericola* | CBS 378.71^T^ | KT214579 | KT214610 | KT214756 | KT214680 | Wang et al. (2016b) |
| *Chaetomium cucumericola* | CBS 126777 | HM365247 | HM365247 | KT214757 | KT214681 | Wang et al. (2016b) |
| *Chaetomium elatum* | CBS 142034^T^ | KX976612 | KX976733 | KX976954 | KX976832 | Wang et al. (2016a) |
| *Chaetomium elatum* | CBS 374.66 | KC109758 | KC109758 | KC109776 | KF001820 | Wang et al. (2016a) |
| *Chaetomium fimeti* | CBS 139034^T^ | KT214559 | KT214593 | KT214736 | KT214663 | Wang et al. (2016b) |
| *Chaetomium fimeti* | CBS 153.77 | KT214561 | KT214594 | KT214738 | KT214664 | Wang et al. (2016b) |
| *Chaetomium globosum* | CBS 160.62^T^ | KT214565 | KT214596 | KT214742 | KT214666 | Wang et al. (2016b) |
| *Chaetomium globosum* | CBS 105.40 | KT214566 | KT214597 | KT214743 | KT214667 | Wang et al. (2016b) |
| *Chaetomium globosum* | CBS 147.60 | JN209909 | JN209909 | JN256179 | KF001793 | Wang et al. (2016b) |
| *Chaetomium globosum* | CBS 132.30 | KC109755 | KC109755 | KC109773 | KF001792 | Wang et al. (2016b) |
| *Chaetomium globosum* | CBS 164.62 | JN209920 | JN209920 | JN256190 | KF001796 | Wang et al. (2016b) |
| *Chaetomium globosum* | CGMCC 3.19350 | MN215745 | MN215583 | MN337028 | MN255426 | Raza et al. (2019) |
| *Chaetomium globosum* | LC13503 | MN215746 | MN215584 | MN337029 | MN255427 | Raza et al. (2019) |
| *Chaetomium globosum* | CGMCC 3.9994 | JN209894 | JN209894 | JN256168 | KF001798 | Wang et al. (2016b) |
| *Chaetomium graminiforme* | CBS 506.84^T^ | KT214584 | KT214615 | KT214761 | KT214687 | Wang et al. (2016b) |
| *Chaetomium grande* | CBS 126780^T^ | HM365253 | HM365253 | HM365273 | KT214657 | Wang et al. (2016b) |
| *Chaetomium grande* | CBS 126781 | KT214554 | KT214588 | KT214731 | KT214658 | Wang et al. (2016b) |
| *Chaetomium hongheensis* | KUNCC22-10750^T^ | OR129668 | OR129659 | OR125092 | OR125096 | Yang et al. (2024) |
| *Chaetomium hongheensis* | KUMCC22-10764 | OR129667 | OR129658 | OR125091 | OR125095 | Yang et al. (2024) |
| *Chaetomium interruptum* | CBS 126660^T^ | HM365246 | HM365246 | KT214741 | KT214665 | Wang et al. (2016b) |
| *Chaetomium iranicum* | IRAN 3379C^T^ | – | – | MN520421 | MT273944 | Aghyl et al. (2020) |
| *Chaetomium madrasense* | CBS 315.74^T^ | KC109751 | KC109751 | KC109769 | KF001831 | Wang et al. (2016b) |
| *Chaetomium megalocarpum* | CBS 149.59^T^ | KC109744 | KC109744 | KC109762 | KF001828 | Wang et al. (2016b) |
| *Chaetomium megalocarpum* | CBS 778.71 | KC109747 | KC109747 | KC109765 | KF001827 | Wang et al. (2016b) |
| *Chaetomium meridionalense* | COAD 3451^T^ | ON989660 | ON979679 | ON988187 | OP131292 | Condé et al. (2023) |
| *Chaetomium meridionalense* | COAD 3122 | ON989661 | ON979680 | ON988188 | OP131291 | Condé et al. (2023) |
| *Chaetomium microthecium* | CGMCC 3.17556^T^ | KP336785 | KP336834 | KP336883 | KT149505 | Zhang et al. (2017a) |
| *Chaetomium microthecium* | CGMCC 3.17558 | KP336787 | KP336836 | KP336885 | KT149507 | Zhang et al. (2017a) |
| *Chaetomium neoglobosporum* | CBS 108.83^T^ | KC109750 | KC109750 | KC109768 | KF001825 | Wang et al. (2016b) |
| *Chaetomium nepalense* | CBS 288.83 | MH861591 | MH873316 | – | MZ342983 | Wang et al. (2022) |
| *Chaetomium novozelandicum* | CBS 124555^T^ | KT214576 | KT214607 | KT214753 | KT214677 | Wang et al. (2016b) |
| *Chaetomium novozelandicum* | CBS 128484 | KT214578 | KT214609 | KT214755 | KT214679 | Wang et al. (2016b) |
| *Chaetomium nozdrenkoae* | CBS 163.62^T^ | KT214556 | KT214590 | KT214733 | KT214660 | Wang et al. (2016b) |
| *Chaetomium nozdrenkoae* | CBS 809.68 | KT214557 | KT214591 | KT214734 | KT214661 | Wang et al. (2016b) |
| *Chaetomium olivaceum* | CBS 418.80A | JN209914 | JN209914 | JN256184 | KF001806 | Wang et al. (2016b) |
| *Chaetomium olivaceum* | CGMCC 3.12883 | JN209911 | JN209911 | JN256181 | KF001807 | Wang et al. (2016b) |
| *Chaetomium pilosum* | CBS 335.67^T^ | KT214586 | FJ666356 | KT214763 | FJ666387 | Wang et al. (2016b) |
| *Chaetomium pseudocochliodes* | CGMCC 3.9441^T^ | JN209925 | JN209925 | JN256195 | KF001816 | Wang et al. (2016b) |
| *Chaetomium pseudocochliodes* | CGMCC 3.9469 | JN209926 | JN209926 | JN256196 | KF001815 | Wang et al. (2016b) |
| *Chaetomium pseudoglobosum* | CBS 574.71^T^ | KT214573 | KT214604 | KT214750 | KT214674 | Wang et al. (2016b) |
| *Chaetomium rectangulare* | CBS 126778^T^ | HM365239 | HM365239 | HM365285 | KT214688 | Wang et al. (2016b) |
| *Chaetomium rectangulare* | CBS 126658 | HM365240 | HM365240 | HM365286 | KT214689 | Wang et al. (2016b) |
| *Chaetomium spiculipilium* | CBS 373.66^T^ | KC109756 | KC109756 | KC109774 | KF001809 | Wang et al. (2016b) |
| *Chaetomium spirochaete* | CBS 730.84^T^ | JN209921 | JN209921 | JN256191 | KF001819 | Wang et al. (2016b) |
| *Chaetomium spirochaete* | CBS 165.52 | KT214585 | KT214616 | KT214762 | KT214690 | Wang et al. (2016b) |
| *Chaetomium subaffine* | CBS 637.91^T^ | JN209929 | JN209929 | JN256199 | KF001817 | Wang et al. (2016b) |
| *Chaetomium subaffine* | CGMCC 3.14297 | JN209928 | JN209928 | JN256198 | KF001818 | Wang et al. (2016b) |
| *Chaetomium subfimeti* | CBS 370.66^T^ | KT214562 | FJ666354 | KT214739 | FJ666385 | Wang et al. (2016b) |
| *Chaetomium subfimeti* | CBS 169.71 | KT214563 | FJ666357 | KT214740 | FJ666388 | Wang et al. (2016b) |
| *Chaetomium subglobosum* | CBS 149.60^T^ | JN209930 | JN209930 | JN256200 | KF001808 | Wang et al. (2016b) |
| *Chaetomium subglobosum* | CBS 483.73 | KT214581 | KT214612 | KT214758 | KT214684 | Wang et al. (2016b) |
| *Chaetomium tarraconense* | CBS 101882^T^ | – | – | MZ343005 | MZ342964 | Wang et al. (2022) |
| *Chaetomium tectifimeti* | CBS 142032^T^ | KX976640 | KX976737 | KX976982 | KX976836 | Wang et al. (2016a) |
| *Chaetomium telluricola* | CBS 151.59^T^ | KT214582 | KT214613 | KT214759 | KT214685 | Wang et al. (2016b) |
| *Chaetomium tenue* | CBS 139.38^T^ | KT214568 | KT214599 | KT214745 | KT214669 | Wang et al. (2016b) |
| *Chaetomium tenue* | CBS 143.38 | KT214572 | KT214603 | KT214749 | KT214673 | Wang et al. (2016b) |
| *Chaetomium umbonatum* | CBS 293.83^T^ | KT214575 | KT214606 | KT214752 | KT214676 | Wang et al. (2016b) |
| *Chaetomium undulatulum* | CBS 126775^T^ | HM365251 | HM365251 | HM365279 | KT214682 | Wang et al. (2016b) |
| *Chaetomium undulatulum* | CBS 126776 | HM365250 | HM365250 | HM365278 | KT214683 | Wang et al. (2016b) |
| *Chaetomium unguicola* | CBS 128446^T^ | KT214567 | KT214598 | KT214744 | KT214668 | Wang et al. (2016b) |
| *Chrysanthotrichum allolentum* | CBS 644.83^T^ | MK926808 | MK926808 | MK926908 | MK876768 | Wang et al. (2019a) |
| *Chrysanthotrichum lentum* | CBS 339.67^T^ | MK926809 | MK926809 | MK926909 | MK876769 | Wang et al. (2019a) |
| *Collariella anguipilia* | CBS 632.83^T^ | MZ334721 | MZ351424 | MZ343028 | MZ342989 | Wang et al. (2022) |
| *Collariella bostrychodes* | CBS 163.73 | KX976641 | KX976738 | KX976983 | KX976837 | Wang et al. (2016a) |
| *Collariella bostrychodes* | CBS 586.83 | KX976642 | KX976739 | KX976984 | KX976838 | Wang et al. (2016a) |
| *Collariella capillicompacta* | IRAN 3496C^T^ | – | – | MN520423 | MN520427 | Mehrabi-Koushki et al. (2020) |
| *Collariella carteri* | CBS 128.85^T^ | KX976647 | KX976742 | KX976989 | KX976841 | Wang et al. (2016a) |
| *Collariella causiiformis* | CBS 792.83 | KX976646 | KX976741 | KX976988 | KX976840 | Wang et al. (2016a) |
| *Collariella hexagonospora* | CBS 171.84 | MH861717 | – | MZ343016 | MZ342977 | Wang et al. (2022) |
| *Collariella hilkhuijsenii* | CBS 143305 | MG432011 | MG432012 | MF716586 | MF716587 | Wang (2017) |
| *Collariella hongheensis* | KUNCC 22-10749^T^ | PQ014644 | PQ014642 | PP766710 | PP766708 | Manawasinghe et al. (2024) |
| *Collariella hongheensis* | KUNCC 22-10757 | PQ014645 | PQ014643 | PP766711 | PP766709 | Manawasinghe et al. (2024) |
| *Collariella pachypodioides* | CBS 164.52 | MH856980 | MH868500 | MZ343014 | MZ342975 | Wang et al. (2022) |
| *Collariella quadrangulata* | CBS 142.58 | KX976650 | KX976745 | KX976992 | KX976844 | Wang et al. (2016a) |
| *Collariella quadrangulata* | CBS 152.59 | KX976651 | KX976746 | KX976993 | KX976845 | Wang et al. (2016a) |
| *Collariella quadrum* | CGMCC 3.17917 | KU746675 | KU746721 | KU746767 | KY575870 | Zhang et al. (2017a) |
| *Collariella robusta* | CBS 551.83^T^ | KX976652 | KX976747 | KX976994 | KX976846 | Wang et al. (2016a) |
| *Collariella robusta* | CBS 508.84 | KX976653 | KX976748 | KX976995 | KX976847 | Wang et al. (2016a) |
| *Dichotomopilus dolichotrichus* | CBS 162.48T | HM449049 | JF772462 | HM449063 | KX976852 | Wang et al. (2016a) |
| *Dichotomopilus dolichotrichus* | CGMCC 3.14189 | HM449048 | JF772455 | HM449062 | KX976853 | Wang et al. (2016a) |
| *Dichotomopilus endophyticus* | COAD 3645 | – | – | OR614381 | OR611982 | dos Santos Santana et al. (2026) |
| *Dichotomopilus endophyticus* | COAD 3646 | OR626088 |  | OR614382 | OR611983 | dos Santos Santana et al. (2026) |
| *Dichotomopilus endophyticus* | COAD 3647^T^ | OR626089 |  | OR614383 | OR611981 | dos Santos Santana et al. (20265) |
| *Dichotomopilus erectus* | CBS 140.56^T^ | HM449044 | HM449058 | JF772458 | KX976854 | Wang et al. (2016) |
| *Dichotomopilus erectus* | CGMCC 3.12900 | KC109760 | KC109760 | KC109778 | KX976855 | Wang et al. (2016) |
| *Dichotomopilus finlandicus* | SZMC 26529^T^ | MW541926 | – | MZ665529 | MZ665531 | Kedves et al. (2021) |
| *Dichotomopilus funicola* | CBS 159.52^T^ | GU563369 | GU563354 | JF772461 | KX976856 | Wang et al. (2016) |
| *Dichotomopilus funicola* | CBS 136.38 | HM449046 | HM449060 | JF772457 | KX976857 | Wang et al. (2016) |
| *Dichotomopilus fusus* | CBS 372.66^T^ | KX976660 | KX976754 | KX977002 | KX976859 | Wang et al. (2016) |
| *Dichotomopilus fusus* | CBS 114.83 | KX976661 | KX976755 | KX977003 | KX976860 | Wang et al. (2016) |
| *Dichotomopilus indicus* | CGMCC 3.14184^T^ | GU563367 | GU563360 | JF772453 | KX976861 | Wang et al. (2016) |
| *Dichotomopilus indicus* | CGMCC 3.14182 | GU563366 | GU563358 | JF772451 | KX976862 | Wang et al. (2016) |
| *Dichotomopilus pratensis* | CBS 133396^T^ | GU563372 | GU563357 | JF772450 | KX976866 | Wang et al. (2016) |
| *Dichotomopilus pratensis* | CBS 804.83 | KX976665 | KX976759 | KX977007 | KX976867 | Wang et al. (2016) |
| *Dichotomopilus pseudoerectus* | CBS 252.75^T^ | KX97666 | KX976761 | KX977009 | KX976869 | Wang et al. (2016) |
| *Dichotomopilus pseudofunicola* | CBS 142033^T^ | KX976668 | KX976762 | KX977010 | KX976870 | Wang et al. (2016) |
| *Dichotomopilus ramosissimus* | CGMCC 3.14183^T^ | GU563371 | GU563361 | JF772452 | KX976871 | Wang et al. (2016) |
| *Dichotomopilus ramosissimus* | CGMCC 3.12930 | HM449045 | HM449059 | JF772449 | KX976872 | Wang et al. (2016) |
| *Dichotomopilus reflexus* | CBS 157.49^T^ | HM449051 | HM449055 | JF772460 | KX976873 | Wang et al. (2016) |
| *Dichotomopilus reflexus* | CBS 141.56 | KX976669 | KX976763 | KX977011 | KX976874 | Wang et al. (2016) |
| *Dichotomopilus subfunicola* | CGMCC 3.12892^T^ | JX867125 | JX867125 | JX867122 | KX976875 | Wang et al. (2016) |
| *Dichotomopilus subfunicola* | CGMCC 3. 9466 | GU563368 | GU563353 | JF772446 | KX976876 | Wang et al. (2016) |
| *Dichotomopilus subfunicola* | CBS 812.73 | KX976670 | KX977012 | KX976764 | KX976877 | Wang et al. (2016a) |
| *Dichotomopilus subfunicola* | CBS 794.83 | KX976671 | KX977013 | KX976765 | KX976878 | Wang et al. (2016a) |
| *Dichotomopilus variostiolatus* | CBS 179.84^T^ | KX976672 | KX976766 | KX977014 | KX976879 | Wang et al. (2016) |
| *Dichotomopilus variostiolatus* | DTO 319-A2 | KX976673 | KX977015 | KX976767 | KX976880 | Wang et al. (2016a) |
| *Floropilus chiversii* | CBS 558.80^T^ | MK926818 | MK926818 | MK926918 | MK876778 | Wang et al. (2019a) |
| *Humicola ampulliella* | CBS 116735^T^ | LT993568 | LT993568 | LT993649 | LT993487 | Wang et al. (2019b) |
| *Humicola ampulliella* | CBS 116736 | LT993569 | LT993569 | LT993650 | LT993488 | Wang et al. (2019b) |
| *Humicola cerradensis* | URM 9157^T^ | PQ268215 | PQ249846 | PQ268491 | PQ268495 | Nogueira et al. (2025) |
| *Humicola cerradensis* | FCCUFG85 | PQ268216 | PQ249847 | PQ268492 | PQ268496 | Nogueira et al. (2025) |
| *Humicola christenseniae* | CBS 127760^T^ | LT993571 | LT993571 | LT993652 | LT993490 | Wang et al. (2019b) |
| *Humicola christenseniae* | CGMCC 3.08497 | LT993572 | LT993572 | LT993653 | LT993491 | Wang et al. (2019b) |
| *Humicola cuyabenoensis* | CBS 398.97^T^ | LT993573 | LT993573 | LT993654 | LT993492 | Wang et al. (2019b) |
| *Humicola degenerans* | CBS 232.65^T^ | LT993574 | LT993574 | LT993655 | LT993493 | Wang et al. (2019b) |
| *Humicola degenerans* | CBS 780.71 | LT993575 | LT993575 | LT993656 | LT993494 | Wang et al. (2019b) |
| *Humicola distorta* | CBS 417.66^T^ | LT993577 | LT993577 | LT993658 | LT993496 | Wang et al. (2019b) |
| *Humicola floriformis* | CBS 815.97^T^ | LT993578 | LT993578 | LT993659 | LT993497 | Wang et al. (2019b) |
| *Humicola fuscoatra* | CBS 118.14^T^ | LT993579 | LT993579 | LT993660 | LT993498 | Wang et al. (2019b) |
| *Humicola fuscoatra* | CGMCC 3.13428 | LT993580 | LT993580 | LT993661 | LT993499 | Wang et al. (2019b) |
| *Humicola fuscogrisea* | CGMCC 3.13790^T^ | LT993581 | LT993581 | LT993662 | LT993500 | Wang et al. (2019b) |
| *Humicola hirsuta* | CBS 144492^T^ | MZ334726 | MZ351425 | MZ343013 | MZ342974 | Wang et al. (2022) |
| *Humicola homopilata* | CBS 157.55^T^ | LT993582 | LT993582 | LT993663 | LT993501 | Wang et al. (2019b) |
| *Humicola homopilata* | CBS 338.68 | LT993583 | LT993583 | LT993664 | LT993502 | Wang et al. (2019b) |
| *Humicola leptodermospora* | CBS 120095^T^ | LT993584 | LT993584 | LT993665 | LT993503 | Wang et al. (2019b) |
| *Humicola malaysiensis* | CBS 399.97^T^ | LT993586 | LT993586 | LT993667 | LT993505 | Wang et al. (2019b) |
| *Humicola malaysiensis* | CBS 760.83 | LT993587 | LT993587 | LT993668 | LT993506 | Wang et al. (2019b) |
| *Humicola mutabilis* | CBS 779.71^T^ | LT993588 | LT993588 | LT993669 | LT993507 | Wang et al. (2019b) |
| *Humicola olivacea* | CBS 142031^T^ | LT993589 | LT993589 | LT993670 | LT993508 | Wang et al. (2019b) |
| *Humicola pinnata* | CBS 467.66^T^ | LT993590 | LT993590 | LT993671 | LT993509 | Wang et al. (2019b) |
| *Humicola quadrangulata* | CBS 111771^T^ | LT993593 | LT993593 | LT993674 | LT993512 | Wang et al. (2019b) |
| *Humicola seminuda* | CBS 368.84^T^ | LT993594 | LT993594 | LT993675 | LT993513 | Wang et al. (2019b) |
| *Humicola seminuda* | CBS 549.69 | LT993596 | LT993596 | LT993677 | LT993515 | Wang et al. (2019b) |
| *Humicola sphaeralis* | CBS 985.87^T^ | LT993598 | LT993598 | LT993679 | LT993517 | Wang et al. (2019b) |
| *Humicola udagawae* | CBS 337.68^T^ | LT993601 | LT993601 | LT993682 | LT993520 | Wang et al. (2019b) |
| *Humicola wallefii* | CBS 147.67^T^ | LT993602 | LT993602 | LT993683 | LT993521 | Wang et al. (2019b) |
| *Intraradix grisea* | COAD 3910^T^ | PQ898842 | PQ898841 | PQ898405 | PQ898404 | dos Santos Santana et al. (2026) |
| *Intraradix grisea* | URM 9211 | PQ898843 | – | PQ898406 | PQ898403 | dos Santos Santana et al. (2026) |
| *Parahumicola guana* | COAD 3110^T^ | ON989659 | OP108407 | OP131290 | ON995381 | Condé et al. (2023) |
| *Pseudohumicola alba* | COAD 3126^T^ | ON989662 | ON979681 | ON988189 | ON995382 | Condé et al. (2023) |
| *Pseudohumicola atrobrunnea* | CBS 114167^T^ | LT993570 | LT993570 | LT993651 | LT993489 | Wang et al. (2019b) |
| *Pseudohumicola cecavii* | URM 8444^T^ | ON862932 | – | OP672391 | OP722570 | Alves et al. (2022) |
| *Pseudohumicola cecavii* | R35 | – | – | OP672392 | OP722571 | Alves et al. (2022) |
| *Pseudohumicola cinnamomeobrunnea* | CBS 150900^T^ | OZ016267 | OZ016268 | OZ016231 | OZ016230 | Sastoque et al. (2025) |
| *Pseudohumicola debertoldii* | CBS 460.76^T^ | MH860994 | MH872765 | MH444288 | OZ016246 | Sastoque et al. (2025) |
| *Pseudohumicola glauca* | CBS 462.76^T^ | MH444273 | OZ016244 | MH444286 | OZ016245 | Sastoque et al. (2025) |
| *Pseudohumicola intercalarispora* | CBS 149962^T^ | OZ001682 | OZ001683 | OZ001724 | OZ001723 | Sastoque et al. (2025) |
| *Pseudohumicola lutea* | COAD 3127^T^ | ON989663 | ON979682 | ON988190 | ON995383 | Condé et al. (2023) |
| *Pseudohumicola pulvericola* | CBS 144165^T^ | LT993591 | LT993591 | LT993672 | LT993510 | Wang et al. (2019b) |
| *Pseudohumicola pulvericola* | CBS 144166 | LT993592 | LT993592 | LT993673 | LT993511 | Wang et al. (2019b) |
| *Pseudohumicola semispiralis* | CBS 723.97^T^ | LT993597 | LT993597 | LT993678 | LT993516 | Wang et al. (2019b) |
| *Pseudohumicola subspiralis* | CBS 148.58 | LT993599 | LT993599 | LT993680 | LT993518 | Wang et al. (2019b) |
| *Pseudohumicola subspiralis* | CBS 119768 | LT993600 | LT993600 | LT993681 | LT993519 | Wang et al. (2019b) |
| *Stellatospora terricola* | CBS 811.95^T^ | MK926835 | MK926835 | MK926935 | MK876797 | Wang et al. (2019a) |
| *Thermothielavioides terrestris* | CBS 117535^T^ | MK926837 | MK926837 | MK926937 | MK876799 | Wang et al. (2022) |
| *Trichocladium acropullum* | CBS 114580^T^ | LT993626 | LT993626 | LT993707 | LT993545 | Wang et al. (2019b) |
| *Trichocladium asperum* | CBS 903.85^T^ | LT993632 | LT993632 | LT993713 | LT993551 | Wang et al. (2019b) |
| *Xanthiomyces spinosus* | CBS 789.71 | MH860357 | MZ351429 | MZ343034 | MZ342995 | Wang et al. (2022) |
| *Xanthiomyces spinosus* | CBS 796.83 | MZ334724 | MZ351430 | MZ343035 | MZ342996 | Wang et al. (2022) |

“T” = ex-type strains
